# Genomic incompatibilities are persistent barriers when speciation happens with gene flow in *Formica* ants

**DOI:** 10.1093/molbev/msag063

**Published:** 2026-03-11

**Authors:** Patrick Heidbreder, Noora Poikela, Pierre Nouhaud, Tuomas Puukko, Konrad Lohse, Jonna Kulmuni

**Affiliations:** Organismal and Evolutionary Biology Research Programme, University of Helsinki, Helsinki 00560, Finland; Tvärminne Zoological Station, University of Helsinki, Hanko, Finland; Organismal and Evolutionary Biology Research Programme, University of Helsinki, Helsinki 00560, Finland; CBGP, INRAE, CIRAD, Institut Agro, IRD, Univ Montpellier, Montpellier, France; Organismal and Evolutionary Biology Research Programme, University of Helsinki, Helsinki 00560, Finland; Institute of Ecology and Evolution, University of Edinburgh, Edinburgh EH9 3JR, UK; Organismal and Evolutionary Biology Research Programme, University of Helsinki, Helsinki 00560, Finland; Tvärminne Zoological Station, University of Helsinki, Hanko, Finland; Institute for Biodiversity and Ecosystem Dynamics, Department of Evolutionary and Population Biology, University of Amsterdam, Amsterdam 1098 XH, Netherlands

**Keywords:** barrier loci, Bateson-Dobzhansky-Muller incompatibility (BDMI), network, demographic modeling, speciation, evolutionary biology, evolutionary genetics and genomics

## Abstract

A current goal of speciation research is identifying loci underlying reproductive barriers between species. Locating barrier loci in population genomic data is difficult due to the often-complex demographic history of diverged taxa and heterogeneity in evolutionary forces across the genome. We take advantage of natural hybridization between 2 wood ant species (*Formica aquilonia* and *Formica polyctena*) to identify regions of reduced long-term gene flow using demographically explicit scans of nonadmixed genomes. In addition, we identify candidate Bateson-Dobzhansky-Muller incompatibilities (BDMIs) through an imbalanced recombinant haplotype frequency analysis using a large sample of natural *F. aquilonia* × *F. polyctena* hybrid genomes. These approaches find barriers and BDMIs scattered across the genome. Furthermore, BDMIs significantly overlap with long-term barriers, indicating that some BDMIs have persisted despite divergence with gene flow. Intriguingly, the number of pairwise interactions a BDMI has correlates with its long-term barrier strength: hub-like BDMIs with many interactions reduce gene flow more effectively. Finally, we find that long-term barriers are depleted for both coding sequences (CDS) and transposable elements (TEs), while candidate BDMIs are associated with snRNAs and LTR transposons, specifically *Ty1-copia*. In contrast, regions where long-term barriers and BDMIs co-locate are significantly associated with introns but not CDS or TEs, implying a potential role of alternative splicing or gene regulation in long-term incompatibilities. Our results highlight the underappreciated impact of BDMI connectivity on the persistence of reproductive barriers over time.

## Introduction

A current goal of speciation genomics is identifying loci or genomic regions that underlie reproductive barriers between diverging lineages (Lindtke and Buerkle 2015; Ravinet et al. 2017; Kulmuni et al. 2020). However, how reproductive barriers and their genomic architecture evolve and lead to reproductive isolation (RI) during speciation with gene flow remains an open question. Broadly defined, a barrier locus is any locus that reduces the flow of neutral alleles between taxa (Barton 1983; Westram et al. 2022) by reducing hybrid fitness (Ravinet et al. 2017). Such reduction may occur because the barrier locus is involved in habitat choice, local adaptation, assortative mating, or through negative epistasis with other loci.

Detecting such barrier loci from genomic data is challenging. A primary method has been to use genome scans of summary statistics to identify outlier genomic windows of divergence between diverging lineages. As barrier loci locally reduce gene flow, they should be associated with locally elevated genetic divergence (*d*_xy_) and differentiation (*F*_ST_) in the genome (Cruickshank and Hahn 2014; Ravinet et al. 2017). However, a fundamental problem with simple genome scans is that summary statistics are affected not only by migration, but also by the stochasticity of drift, heterogeneity in selective forces, as well as the genetic properties (ie mutation, recombination) of the populations being compared (Meirmans and Hedrick 2011). Consequently, summary statistics do not allow us to distinguish regions of reduced migration from elevated divergence or differentiation caused by other population genetic processes. Recognition of the limitations of genome scans has driven the development of new methods to infer barrier regions via a signal of reduced effective migration while controlling for confounding demographic factors (Fraïsse et al. 2021; Laetsch et al. 2023; Burban et al. 2024). While such model-based genome scans identify long-term barriers (those acting since the onset of species divergence) that may encompass various pre- and postzygotic mechanisms which contribute to RI, they are agnostic about the nature of the barrier loci involved and/or whether barrier loci involve epistatic interactions.

For epistatic interactions, a fundamental model in speciation is the Bateson-Dobzhansky-Muller incompatibility (BDMI) model (Bateson 1909; Dobzhansky 1937; Muller 1942). In this model, alleles that arise in 2 separate populations have the potential to reduce the fitness of hybrid individuals when combined in a hybrid genome, as these alleles have not evolved and coexisted in the same genetic background. BDMIs can be purely intrinsic, ie they reduce hybrid fitness independent of the environment (Coyne and Orr 2004), or be impacted by ecological selection as highlighted in recent work (Kulmuni and Westram 2017; Thompson et al. 2024). Theoretical work on the basic BDMI model has focused on rates of BDMI accumulation, the number required for speciation, and the persistence of BDMIs in populations which exchange migrants (Orr 1995; Orr and Turelli 2001; Welch 2004; Bank et al. 2012; Blanckaert and Hermisson 2018; Blanckaert et al. 2020). These studies find that 2-locus BDMIs are not persistent barriers under secondary contact and tend to be selected against and removed (Barton and Bengtson 1986; Xiong and Mallet 2022). In contrast, BDMIs that are also under ecological selection (Bank et al. 2012; Kulmuni and Westram 2017) or involve multiple epistatically interacting loci (Blanckaert et al. 2020), can form more persistent, long-term barriers to gene flow. Biologically more realistic incompatibilities are also considered, where genes interact in biological pathways and epistasis involves multiple loci (Dagilis et al. 2019; Satokangas et al. 2020; Bank 2022; Schiffman and Ralph 2022). However, no empirical studies have investigated whether more connected incompatibilities are more persistent barriers to gene flow over evolutionary time.

Detecting BDMIs can be achieved by investigating ancestry patterns in hybrids, either in laboratory crosses or in natural hybrid populations. Early mapping studies have successfully identified numerous BDMIs in various species, including *Drosophila* (Cabot et al. 1994; Presgraves 2003), *Solanum* (Moyle and Graham 2005; Moyle and Nakazato 2010), and house mice (Larson et al. 2018). However, laboratory crosses are only feasible in suitable model systems. Recently, a statistical method has been developed to identify BDMIs from whole-genome data from natural hybrid populations by detecting an underrepresentation of one of the recombinant genotype combinations between 2 loci (Li et al. 2022; Li and Lee 2023), that and builds and improves upon work with ancestry disequilibrium methods (Schumer et al. 2014). Yet the extent to which BDMIs contribute to persistent, long-term barriers to gene flow remains an open question, and to date, there are few empirical studies examining genomic barriers across time scales (Larson et al. 2018; Ebdon et al. 2025).

We still have little information about the general genomic architecture of species barriers. The null model of a polygenic barrier (Barton and Bengtson 1986) predicts that barrier loci and BDMIs should accumulate in regions of reduced recombination (such as within chromosomal inversions or centromeric regions) (Felsenstein 1981; Butlin 2005), as observed in certain butterfly species (Martin et al. 2019; Laetsch et al. 2023; Mackintosh et al. 2023; Ebdon et al. 2025), and in gene rich regions (Schumer et al. 2018; Ebdon et al. 2025). While the BDMI model is agnostic about the mechanism causing incompatibilities between loci, it is generally expected that BDMIs will arise in functional regions (ie coding regions of genes) or transposable element (TE) insertion sites (Presgraves 2010; Oppold et al. 2017; Campbell et al. 2018). It has also been increasingly recognized that introns and intergenic sequences may play crucial roles in RI, particularly through regulatory elements that influence gene expression (Rieseberg and Blackman 2010; Campbell et al. 2018). For example, aberrant splicing may contribute to RI by reducing hybrid fitness, with spliceosome genes acting as BDMIs (Smith et al. 2021).

Red wood ants of the *Formica rufa* (Hymenoptera: Formicidae) group serve as an excellent model to investigate the nature of barriers to gene flow for several reasons. First, this group contains multiple recently diverged lineages that still hybridize in nature. Despite high rates of hybridization, the species remain distinct in sympatry (Satokangas et al. 2023). Second, ants are arrhenotokous haplodiploids (Mable and Otto 1998), meaning that fertilized eggs develop into diploid females and unfertilized eggs develop into haploid males. While haplodiploidy lowers the effective recombination rate due to haploid males not recombining, ants, as other social insects, have high recombination rates relative to other species (Wilfert et al. 2007; Stapley et al. 2017; DeLory et al. 2024). Haplodiploidy also offers benefits for analyses: Hybrid males can be used to map both recessive and dominant incompatibilities genome-wide without the need for phasing (Nouhaud et al. 2020). Here, we focus on 2 species, *F. aquilonia* and *F. polyctena*. Both are polygynous, with hundreds of reproductive queens within a nest, resulting in low relatedness among nestmates. Polygyny in *Formica* is controlled by a set of 3 inversions on chromosome 3, called the social chromosome (Brelsford et al. 2020), and even though both species are polygynous, they have different forms of the supergene haplotype, generating the potential of barrier enrichment on the social chromosome (Sigeman et al. 2025). Previous reconstructions of the speciation history suggests that the 2 species diverged around 500 kya (Goropashnaya et al. 2012; Portinha et al. 2022), with unidirectional gene flow from *F. aquilonia* into *F. polyctena* (Portinha et al. 2022). In southern Finland, stable natural hybrid populations exist dispersed among populations of *F. aquilonia* (Beresford et al. 2017; Satokangas et al. 2023). One of these hybrid populations has been studied for 20 years (Kulmuni et al. 2010; Nouhaud et al. 2022b). The exact age of this hybrid population is uncertain, but it does not represent early generation hybrids. The interpretation of parameter estimates obtained from demographic modeling is complicated by overlapping generations and an unknown rate of queen turnover. However, based on samples from 2019, the population is estimated to be 20 to 36 generations old and unlikely to be older than 50 generations (Nouhaud et al. 2022b). Despite this, hybrid incompatibilities are still segregating in this hybrid population (Kulmuni et al. 2010; Kulmuni and Pamilo 2014), potentially maintained by environment-dependent selection at barrier loci (Kulmuni et al. 2020).

**Figure 1:**
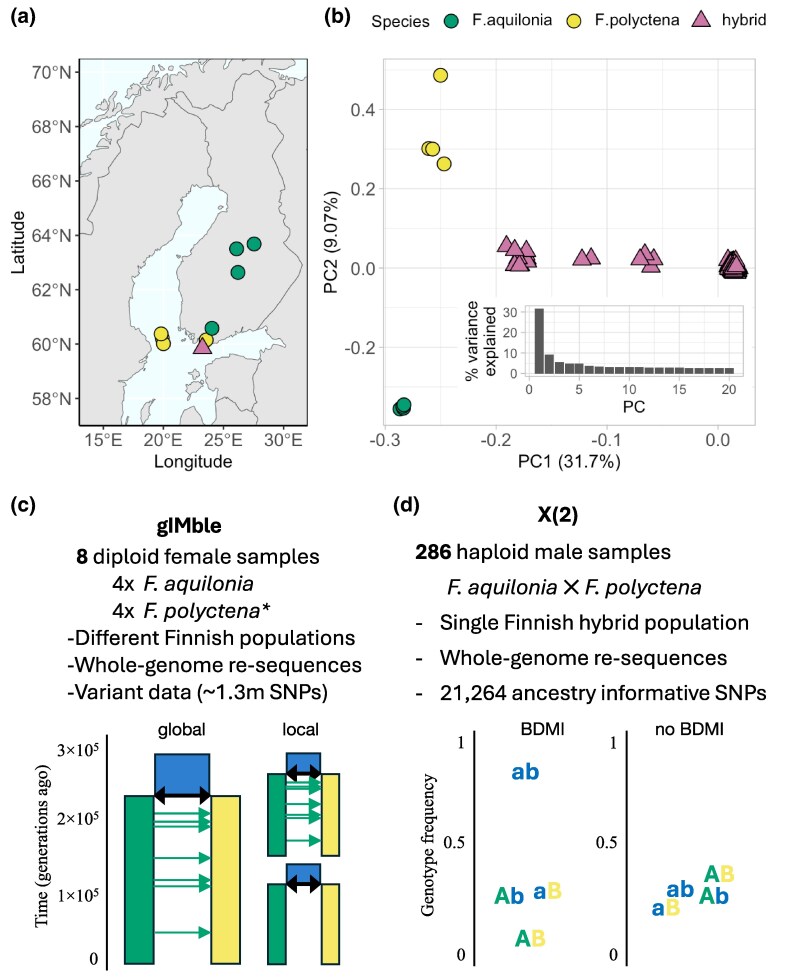
Sampling data and methods used for detecting barrier and incompatibility loci. a) Sampling locations of *Formica aquilonia*, *F. polyctena*, and their hybrids. Green and yellow circles respectively indicate *F. aquilonia* and *F. polyctena* individuals sampled from their sympatric range (8 diploid individuals altogether), purple triangles indicate the Långholmen hybrid population where 286 haploid hybrid individuals were sampled. b) PCA of samples used to detect barrier regions (*F. aquilonia* and *F. polyctena*) in the genome (symbols as in a) and BDMIs (hybrids). Inset barchart is the variance explained by principal components 1 to 20. c) Data used for identifying long-term barriers with gIMble (Laetsch et al. 2023) by comparing a local demographic model of divergence with gene flow (green arrows) in a global genome-wide demographic model (c, global) against the same model from local data in genomic windows. Local genomic windows can have best-fit model parameters that match the global model (c, local top). Or a local genomic window can have significantly less gene flow than the global mode (c, local bottom), indicating a barrier. d) Data used for identifying BDMIs within a natural hybrid population using *X*(2). A BDMI is inferred when one of the recombinant genotypes (AB) is underrepresented in contrast to parental genotypes (Ab, aB) and the alternative recombinant (ab) (c, BDMI). No BDMI is detected for all other genotype frequency combinations, for example equal frequencies (b, no BDMI). Uppercase A and B represent derived alleles in the green and yellow lineages, while blue lowercase a and b represent the ancestral alleles.

Here, we use the *Formica* ant system to identify long-term barriers to gene flow from parental genomes and segregating BDMIs from a young hybrid population (Fig. 1) and ask the following questions. First, how are long-term barriers to gene flow and BDMIs distributed across the genome? In the face of gene flow, both may be expected to become clustered into a few genomic regions, which are easier to maintain by selection than scattered barrier architectures. Second, is there an overlap between the long-term barriers and BDMIs, suggesting that BDMIs can evolve as intrinsic barriers even in the presence of gene flow? Given the different time scales and the fact that long-term barriers may encompass both pre- and postzygotic barriers, whereas BDMIs are solely associated with postzygotic isolation and may easily collapse under gene flow, we expect some but not complete overlap between the different types of barriers. Third, are BDMIs that involve a large number of interacting loci associated with a stronger reduction in long-term gene flow than pairwise BDMIs? We hypothesize that multilocus incompatibilities with more epistatic interactions across the genome are harder to resolve and thus expected to resist gene flow better than simple incompatibilities. Finally, do long-term barriers and BDMIs concentrate in regions of low recombination, and are they associated with genomic features known to be involved in incompatibilities (CDS, introns, or TEs)?

## Results

### Divergence with gene flow has resulted in barriers that are scattered genome-wide

We used 2 complementary and independent methods on 2 distinct datasets to map long-term barriers to gene flow and BDMIs. First, we identified long-term barriers between 4 *F. aquilonia* and 4 *F. polyctena* samples (Fig. 1a and b) using whole-genome resequencing (WGS) data with a demographically explicit scan, gIMble (Laetsch et al. 2023) (v1.0.3): we inferred a genome-wide model of unidirectional postdivergence gene flow from *F. aquilonia* into *F. polyctena*, estimating a divergence time around ∼600 kya and a scaled effective migration rate *M* = 4*N*_e_*m*_e_ = 1.5 migrants per generation (Figure S1, Table S1), which are both consistent with previous findings (Portinha et al. 2022). Then, we identified long-term barrier loci as sliding windows (median length 41.5 kb; Figure S2) that have more support for a strict divergence model (ie *m*_e_ = 0) than the genome-wide background model of effective migration, i.e. Δ_B0_ > 0 (see Materials and methods).

**Figure 2:**
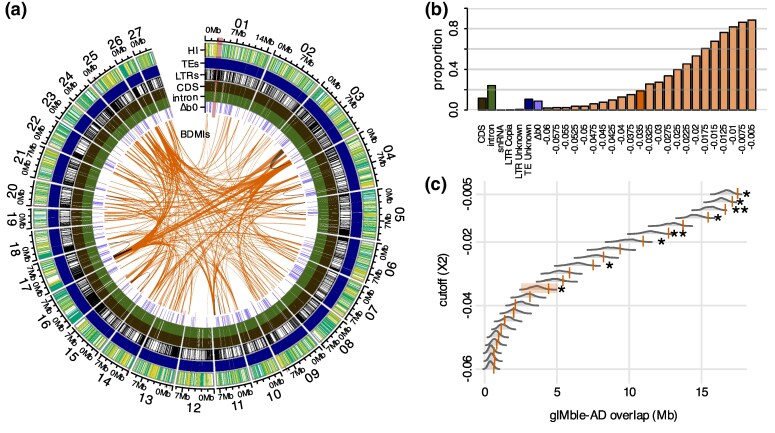
Candidate barriers are genome-wide despite speciation with gene flow. a) Circular plot showing the locations of genetic incompatibilities (BDMIs), long-term barriers to gene flow (gIMble; Δ_B0_ > 0 and FPR ≤ 0.05), introns and coding sequences (CDS), long terminal repeat retrotransposons (LTRs), all transposable element annotations (TEs), and the hybrid index (HI) of genomic regions in 10 kb windows. BDMIs are indicated as links between genomic regions for the *X*(2) = −0.035 cutoff (highlighted in b and c). Intrachromosomal BDMI links are colored orange and intrachromosomal BDMIs are colored black. The inner ring shows long-term barriers in lavender. Moving outwards, the rings indicate introns and CDS, LTRs, all TEs, and HI. The outermost values represent the 27 scaffolds with centromeric regions highlighted across tracks in red. b) Percent coverage of the reference genome for each dataset and *X*(2) cutoff. c) Overlap between BDMIs and long-term barriers. Observed values for each *X*(2) cutoff are plotted as orange lines, bootstrapped distribution values are visualized behind the observed values as gray density plots. Overlaps are marked as significant as following: *P < 0.05, **P < 0.01, ***P < 0.001.

Overall, we found that 5.3% (1,110 out of 21,042) of genomic windows showed Δ_B0_ > 0, indicating potential long-term barriers to gene flow (Figure S3). However, after accounting for a FPR of 5%, 31.4% of these windows (348 out of 1,110) were excluded as potential false positives (Figure S3). By merging overlapping windows, we identified 290 distinct candidates for long-term barrier regions, spanning a total length of 18.8 Mb, or 8.8% of the genome (Figs. 2a and b, Figure S3). Long-term barrier regions varied in length from 33.5 to 215.1 kb (mean 64.7 kb, median 531.74 kb,) and were distributed across all chromosomes, each containing approximately the same fraction of barriers (Fig. 2a, Figure S3, Table S2). Notably, the strongest long-term barriers to gene flow only partially overlapped with *F*_ST_ and *d*_xy_ outliers (Figure S4), demonstrating the importance of identifying candidate barrier regions by explicitly modeling variation in *m*_e_ and accounting for variation in *N*_e_. Mean and window-wise heterozygosity (*H*), *d*_xy_ and *F*_ST_ are shown in Table S3 and Figure S5.

In addition to mapping long-term barrier loci using the demographically explicit scan, we identified BDMIs in a second independent dataset of 286 *F. aquilonia* × *F. polyctena* hybrid haploid male genomes from a contemporary hybrid population in Långholmen, Finland (Fig. 1a, Table S4). We used the *X*(2) statistic (Li et al. 2022) (see Materials and methods) to identify genomic regions housing candidate BDMIs. BDMIs distort the 4 possible 2-locus genotype combinations into a predictable order as the BDMI genotype is selected against over time after a hybridization event. This nonrandom association of alleles, in this case indicative of negative epistasis (and discriminated from population structure [Li and Lee 2023]), is distinct from both nonrandom associations as defined by linkage disequilibrium, and the nonepistatic signals detected by gIMble. We detected candidate BDMIs as pairs of regions showing a signal of negative epistasis defined by a series of increasingly strict *X*(2) cutoffs (see Materials and methods). The number of candidate BDMI pairs identified varied across cutoffs, ranging from 87 to 11,486 (Fig. 2, Table S5). We have used the *X*(2) < −0.035 dataset throughout the manuscript for visualization with all cutoffs available in the Information. The sizes of BDMI loci ranged from 3.0 to 1.7Mb (mean 154 kb, median 116 kb, Table S5) and covered a total span from 4.53 to 188.5Mb (1.9% to 88.5% of the genome) (Fig. 2b, Figure S6). Like the long-term barriers identified by gIMble, candidate BDMIs were found on all chromosomes across the genome, with chromosome 17 having more BDMIs than the genome-wide average (Table S6). We primarily detected interchromosomal BDMIs across all cutoffs (mean interchromosomal proportion 0.96; Table S5).

To understand the interplay between recombination rate variation and long-term barriers to gene flow, we used 2 proxies for low recombination regions. First, we tested if long-term barriers and BDMIs were disproportionately located on the social chromosome (chromosome 3) characterized by known large inversions (Brelsford et al. 2020) but found no significant enrichment of either on the social chromosome (Tables S2 and S6). Second, we calculated the mean distance of long-term barriers and BDMIs from centromeres, finding that both are closer to centromeres than expected by chance (Table S7).

### Incompatibilities act as persistent long-term barriers and are characterized by complex interactions

To evaluate the role of BDMIs as persistent long-term barriers to gene flow, we investigated (i) whether long-term barrier regions and candidate BDMIs co-localize, and (ii) whether the number of interactions a BDMI locus has impacts how strongly they reduce the effective migration rate (*m*_e_). We found an overlap between long-term barriers and BDMIs detected in hybrids. This overlap was significant for the most comprehensive sets of candidate BDMIs (ie including both BDMIs with a weak and strong signal) (Fig. 2c).

**Figure 3:**
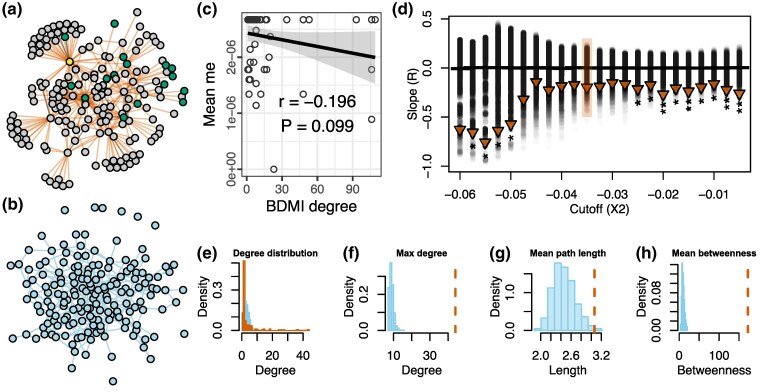
There is connectivity between candidate BDMI loci which impacts how effectively the locus reduces gene flow. Panels a-c and e-h are all visualized for data based on candidate BDMIs at *X*(2) cutoff = −0.035. a) Candidate BDMI regions form a network with many hub-like interactions. Node color indicates the average genomic background of samples in the genomic region the node represents. Yellow nodes have an average hybrid index < − 0.2 indicating a majority *F. polyctena* background, green nodes have an average hybrid index ≥0.8 indicating a majority *F. aquilonia* ancestry, gray nodes have a hybrid index >0.2 or <0.8 indicating admixed ancestry. Observed networks for all *X*(2) cutoffs can be found in Figure S8. b) Visualization of a BDMI network generated by Orr's (1995) model with the same average node degree as the observed BDMI network. c) Long-term barrier strength (Δ_B0_) negatively correlates with the degree (number of inferred BDMIs) of BDMI regions at *X*(2) = −0.035. d) There is negative correlation between Δ_B0_ and degree for all *X*(2) values, and with a significant negative correlation for 13 out of 23 cutoffs. Observed correlation coefficients are plotted as triangles above the bootstrapped distribution of correlation coefficients in black circles. Black horizontal bars represent mean bootstrap correlation coefficients. Observed correlation coefficients are marked as significant as follows: *P < 0.05, **P < 0.01, ***P < 0.001. *X*(2) = −0.035 is highlighted. e) Histogram of the degree distribution of the observed BDMI network in orange, against the histogram of the degree distribution generated by the Orr model in blue. For f to h) the observed BDMI network values are vertical orange dashed lines and the blue histograms are the network value calculated for 1,000 bootstrapped Orr networks. f) Max degree is the highest number of edges that are connected to the same node. g) Mean path length is the average number of edges to travel between 2 random nodes. h) Mean betweenness is the average number of network paths through each node. Observed and bootstrapped Orr model network statistics for all *X*(2) cutoffs can be found in Figure S9.

We quantified the effect of BDMI connectivity on barrier strength for each BDMI region by correlating its number of interactions with other BDMI regions to its impact on effective migration rate (*m*_e_) (Fig. 3a and c). We found that for all X(2) cutoffs, the more connected a BDMI locus, the stronger its reduction in long-term gene flow, with a significant reduction for 13 out of 23 cutoffs (Fig. 3d, Figure S7). Randomization of *m*_e_ values confirmed that the negative correlation between BDMI degree and *m*_e_ is not an artifact of the varying sample sizes (number of loci) across different BDMIs degrees (Fig. 3d).

Analyzing BDMI network statistics, we found that the degree distributions (ie the distribution of the number of connections per node over the whole network) of the observed BDMI networks were much broader than the expected degree distribution of the classic BDMI model as extended by Orr (1995) (Fig. 3b and e). In other words, there were more hub-like BDMIs in the observed incompatibility network that interacted with large numbers of other BDMI loci, compared with a network simulated under Orr's model. Such hubs are predicted when BDMIs follow known protein interactions (Brud and Guerrero 2025). Bootstrapping 1,000 networks revealed further differences across multiple aspects of network topology, including maximum observed node degree, mean path length between nodes, and the average number of neighbors a node has (Fig. 3f to h, Figures S8 and S9). We found that mean betweenness (ie how many network paths pass through a node) in the observed BDMI networks was higher than expected, which also supports hub-like interactions. Together, these results suggest that multi-locus BDMIs can be persistent barriers to gene flow across evolutionary time in natural populations, and that network topology may play a role in their maintenance in the face of continuous gene flow between these *Formica* species.

### Long-term barriers, BDMIs, and their overlap show contrasted association with functional elements and ancestry sorting

**Figure 4:**
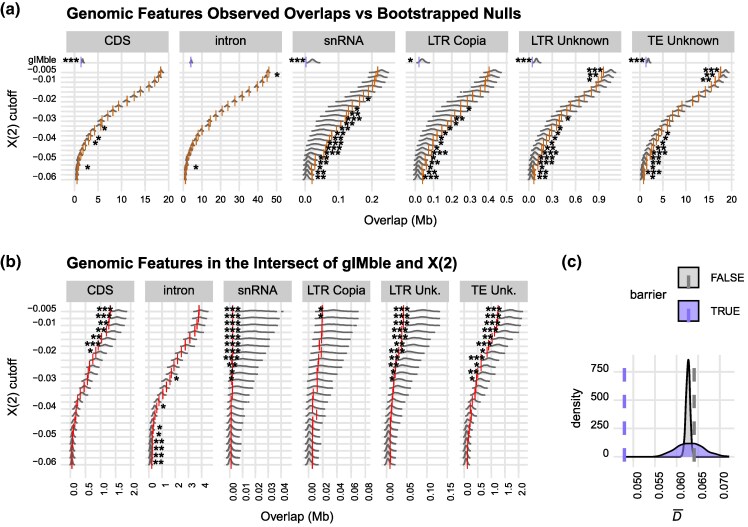
Functional features associated with barriers. a) Overlap between long-term barriers (gIMble, top row) or BDMIs (with all *X*(2) cutoffs) and coding sequences (CDS), introns, snRNA, LTR-Copia elements, unknown LTR elements, and unknown TEs. Observed values for gIMble and each *X*(2) cutoff are plotted as lavender and orange vertical lines, respectively. Bootstrapped distribution values are displayed as gray density plots in the background. Overlaps are marked as significant as follows: *P < 0.05, **P < 0.01, ***P < 0.001. b) Genomic features (as in a) in the regions where long-term gIMble barriers and BDMIs intersect, and CDS, introns, snRNA, LTR-Copia elements, unknown LTR elements, and unknown TEs. Bootstrapped distribution values as in a). Observed overlaps are marked as red vertical lines. c) Observed $\bar{D}$ in barrier windows (purple vertical dashed line) is significantly less than in nonbarrier windows (gray vertical dashed line and gray density distribution; circular resampling, *P* < 0.001) and lower than expected by chance if barriers were randomly distributed (purple density distribution; circular resampling, *P* < 0.001).

We investigated the functional composition of barrier loci in terms of coding sequences (CDS), introns and repetitive elements including TEs, snRNAs, and microsatellites (Figures S16 and S17). We tested if barriers overlap significantly with 3 TE superfamilies: DNA transposons, LINEs, and LTR retrotransposons as well as with Helitrons. Since both DNA transposons and LTRs showed significant associations with candidate BDMIs (Figure S16), we further tested for associations at the subfamily level for these 2 superfamilies (Fig. 4a and b, Figures S18 to S21). Long-term barriers detected by gIMble overlapped significantly less with CDS, snRNAs, and TEs than expected by chance (Fig. 4a, Figures S17, S18, and S20). Candidate BDMIs showed no significant enrichment or depletion of CDS or introns (Fig. 4a). Candidate BDMIs were, however, significantly associated with Ty1-*copia* and unknown LTR retrotransposons, as well as other unidentified TEs (Fig. 4a). Finally, persistent BDMIs—regions where candidate BDMIs overlapped with long-term barriers—were depleted for both CDS and TEs for the least strict cutoffs but showed significant enrichment for introns for the most strict cutoffs (Fig. 4b). The enrichment of introns in the intersection of long-term barriers and BDMIs suggests an important role of regulatory evolution, specifically alternative splicing, in early species divergence.

Finally, we identified a total of 9 genes in persistent BDMIs where there is no gene flow between species (Table S9). Most of these are involved in core cell functions that have been found disrupted by BDMIs identified in other taxa. Two of the genes within persistent BDMI regions are implicated in the development of cancer, aligning with the findings in swordtail fish, where one of the mapped incompatibilities causes melanoma in hybrids (Powell et al. 2020). Furthermore, 2 genes were ribosomal, and 1 involved in maintaining mitochondrial function, all candidate functions that have been associated with incompatibilities in other studies (Barreto and Burton 2012; Lobov et al. 2019; Kitano and Okude 2024; Moran et al. 2024) (Table S9).

Hybrid incompatibilities are expected to be a driver of ancestry sorting in hybrid genomes (Langdon et al. 2024). To understand how ancestry has sorted over time in long-term barrier regions in hybrid genomes, we calculated $\bar{D}$ as the variance in hybrid index (HI) in long-term barrier and nonbarrier regions. $\bar{D}$ close to 0 indicates that ancestry is completely sorted. We found that $\bar{D}$ in the hybrid population is significantly lower in long-term barrier regions (Δ_B0_ > 0) than in nonbarrier regions (2-sided circular resampling, *P* < 0.001, Fig. 4c). Additionally, the observed $\bar{D}$ in barrier regions is lower than expected if the long-term barriers were randomly distributed (2-sided circular resampling, *P* < 0.001, Fig. 4c). This result suggests that multiple generations into a single hybrid population selection drives ancestry sorting toward 1 ancestry component faster in long-term barrier regions than other regions of the genome.

## Discussion

One of the central goals in evolutionary biology is to identify the genetic basis of RI. Theoretical models of speciation predict that intrinsic 2-locus BDMIs are unlikely to persist in the presence of gene flow (Bank et al. 2012). However, little is known about their persistence over longer evolutionary timescales in empirical systems. We find a significant overlap between BDMI regions and long-term barriers between *F. aquilonia* and *F. polyctena*, suggesting that BDMIs have effectively reduced long-term gene flow between these ant species. Intriguingly, we find that the number of pairwise interactions a BDMI has correlates with its long-term barrier strength: BDMIs with many pairwise interactions to other BDMIs reduce gene flow more effectively than those which have fewer interactions. These results highlight the underappreciated impact of multilocus BDMIs as barriers to gene flow.

The 2 wood ant species *F. aquilonia* and *F. polyctena* diverged approximately half a million years ago, exchanging on average 1.5 migrants per generation after their divergence (consistent with previous findings in these species [Portinha et al. 2022]). We found that about 8% of the genome has acted as persistent long-term barriers to gene flow in sympatry. Instead of being localized to a few regions of the genome, as for example in *Heliconius* butterflies (Laetsch et al. 2023), these long-term barriers are scattered throughout the genome, supporting a growing body of evidence of polygenic barriers to gene flow (Barton and Gale 1993; Larson et al. 2018; Lobov et al. 2019; Kitano and Okude 2024). We also found a similar genome-wide distribution pattern for candidate BDMIs. Finding a genome-wide barrier is somewhat surprising considering that the focal species have diverged with gene flow. While co-expressed genes often cluster on the same chromosome, and most physical protein interactions are intrachromosomal (Hurst et al. 2004; Szabo et al. 2019), we did not find any evidence for an enrichment of intrachromosomal BDMIs, in agreement with a previous BDMI scan in swordtail fish (Li et al. 2022). However, unlike our definition of long-term barriers, the screen for BDMIs relies on arbitrary cutoffs, which complicates direct comparisons with the number of BDMIs identified in other systems or theoretical expectations. Despite these methodological differences, we find overlap between candidate BDMIs and long-term barriers between 2 wood ant species who diverged with gene flow. This indicates that some of the candidate BDMIs persist in the face of gene flow over long evolutionary timescales.

The BDMIs that were found to most effectively reduce long-term gene flow had 2 distinct and surprising features: (i) they were multilocus, ie involving more pairwise interactions than expected by chance for a range of *X*(2) cutoffs and (ii) they were significantly associated with introns but not with CDS. While the simplest BDMI model involves 2 loci and 2 alleles (Muller 1942; Welch 2004), such pairwise incompatibilities are not expected to be able to contribute to long-term barriers in the face of gene flow, as they can be easily purged following introgression (Bank et al. 2012; Blanckaert and Hermisson 2018). In contrast, the complexity of a BDMI increases with the number of alleles (Lindtke and Buerkle 2015) or loci involved (Blanckaert et al. 2020; Ayala-López and Bank 2025), and BDMIs involving epistasis between 3 or more loci can act as strong barriers even with gene flow (Blanckaert et al. 2020). However, these theoretical expectations have not been tested in empirical data. Here we use the number of pairwise interactions that each candidate BDMI locus is involved in (ie the BDMI degree) as a proxy for the complexity of incompatibilities. Thus, our finding that the more pairwise interactions a BDMI has, the more effectively it reduces gene flow, suggests that BDMI complexity determines the long-term maintenance of a BDMI. We note that in the limit, a locus that is involved in a large number of negative epistatic interactions, becomes incompatible with its entire genetic background and it becomes nonsensical to attempt to identify individual incompatibilities. Furthermore, the network of BDMIs we observe is much more connected than predicted by classic null models of incompatibility accumulation (Orr 1995). Our results, together with other recent studies (Livingstone et al. 2012; Fraïsse et al. 2014; Satokangas et al. 2020) highlight the need to consider more realistic network models in future BDMI work, and further investigations of the importance of network topology. The fact that persistent BDMIs have overrepresentation of intronic sequences rather than CDS is surprising. These results may indicate that BDMIs that most strongly resist gene flow involve regulatory elements that affect gene expression. A possible explanation may lie in the increased connectivity of barriers. While we did not test if the candidate BDMI regions are hub-genes per se (ie we do not test for overlap with PPI networks), hub genes are known to be largely involved in regulatory roles, for example in *Caenorhabditis elegans* and yeast (Borneman et al. 2006; Lehner et al. 2006). Such involvement of regulatory regions would be in line with previous work on regulation being important for species divergence and hybrid dysfunction (Wittkopp and Kalay 2012; Mack and Nachman 2017). The pattern of candidate BDMI association with introns but not CDS may also reflect a survivorship bias. If BDMIs in CDS are more deleterious, they would be purged more quickly compared with intronic BDMIs resulting in no significant overlap between BDMIs and CDS (Halligan and Keightley 2006; Poikela et al. 2023). This is not mutually exclusive with the importance of regulation, and further work is needed to find the main drivers of these results, eg by contrasting observations across hybrid populations of varying ages. We also note here that the depletion of CDS in barrier regions is not due to filtering out SNPs in CDS regions while detecting long-term barriers (ie only neutral loci were used) because the windows used for the gIMble analysis still span over CDS.

Our results also provide potential insight into the role of TEs in long-term barrier persistence. We found that Ty1-*copia* retrotransposons are associated with BDMIs (Fig. 4a), but not in BDMI regions that overlap with long-term barriers (Fig. 4b). We suggest 2 possible explanations. The first is that while TEs could be involved in incompatibility and thus RI in this study system, their contribution over longer timescales would be less important. Rather, deeper genomic changes (eg alternative splicing) seem to play a role in long-term barrier persistence. Second, TEs may be a persistent RI barrier allowing for divergence but leave no direct signature in the form of insertions. Overall, there is much room for further investigation of the role of TEs in the wood ant system.

Why do we not see complete overlap between long-term barriers and candidate BDMI regions? We would expect any overlap to be incomplete for 2 reasons. First, long-term barriers may be associated with both prezygotic and postzygotic isolating mechanisms, while BDMIs are usually involved in the latter (Burke and Arnold 2001; Kulmuni and Westram 2017). For example, since both ant species differ both in their ecological niches and mating flight times (Véle et al. 2009; Douwes 2012; Stockan and Robinson 2016), long-term barriers might involve both habitat and temporal isolation. Second, under a scenario of divergence with gene flow, many emerging BDMIs are constantly being selected against and removed, particularly if they involve recessive alleles which are exposed to purging selection in haploid males (Nouhaud et al. 2020). This could also explain why we did not find any significant overlap between long-term barriers and the most extreme BDMI candidates, which would have been purged quickly after initial admixture in the Långholmen population (estimated around 20 to 36 generations ago [Nouhaud et al. 2022b). To date, only a few studies have quantified genetic barriers across multiple time scales. A recent meta-analysis by Frayer and Payseur (2024) compared incompatibilities and barrier loci identified in lab crosses with barrier estimates from a natural hybrid zone in the house mouse, finding no significant overlap and suggest that either incompatibilities may have already been purged from the hybrid zone studied or do not constitute strong barriers in nature. In contrast, Ebdon et al. (2025) observed a significant overlap between long-term and recent barriers in a natural hybrid zone in *Iphiclides* butterflies. Such overlap, when found, implies an overlap between current barriers and those important to species divergence. More formal overlap comparisons between barriers acting on time scales as in these 2 and the present study will be informative as to the nature of barriers and their importance to initial divergence.

While studies that map incompatibilities directly using laboratory crossing experiments in model organisms have limited resolution (Matute et al. 2010; Frayer and Payseur 2024), indirect scans for BDMIs based on population genomic data from natural hybrid populations suggest that testing for nonrandom associations between epistatically interacting alleles poses other challenges: The fact that all pairwise interactions must be examined (in this study we had 13,325,703 possible window comparisons), means that multiple testing leads to difficulties in identifying significant outliers (Ayala-López and Bank 2025). Embracing this complexity, we conducted all analyses for 23 subsets of candidate BDMIs defined using increasingly strict cutoffs corresponding to sets of BDMI pairs between regions with increasingly distorted ancestry combinations (Figure S10). The main limitation of this approach is that we could not meaningfully estimate the absolute number of BDMIs between the 2 species. Thus, an obvious avenue for future research will be to obtain quantitative predictions for both the number and complexity of BDMIs and the LD structure associated with them in samples from contemporary hybrid populations.

Another promising future direction is to intersect BDMI and barrier scans with direct estimates of the recombination map and structural variants (which is not yet available for *Formica* ants) to test to what extent long-term barriers and BDMIs accumulate in regions of low recombination and recombination modifiers such as inversions. We investigated 2 genomic features expected to have reduced recombination, the social chromosome with known inversions, and centromere regions. We found no enrichment of barriers in the social chromosome (Table S6). However, both candidate BDMIs and long-term barriers were found closer to centromeres than expected by chance (Table S7), supporting the idea that barriers accumulate in low recombination regions. The fact that we found no enrichment for long-term barriers in the social chromosome could be due to the collinearity of polygynous haplotypes within the inversion on the social chromosome in both focal species, even if these haplotypes may not be similar sequence-wise. Therefore, we would highlight the association with centromeres as conforming with expectations of finding barriers in regions of low recombination.

We have previously found repeatable sorting of ancestry in replicate hybrid populations likely driven by purging of deleterious load and selection for haplotypes with recent sweeps (Nouhaud et al. 2022b). Based on the current results, long-term barrier regions also appear to influence hybrid genome evolution. The finding that the mean variance in HI $\left(\bar{D}\right)$ in barrier regions is smaller than in nonbarrier regions is consistent with faster sorting toward 1 ancestry component in the former. The increased sorting of barriers in a contemporary hybrid population is consistent with the persistence of these long-term barrier regions over evolutionary time. A key component of this result is that the age of the hybrid population is old enough for sorting to have occurred (Nouhaud et al. 2022b). This further shows the potential importance of barriers in the predictability of the genetic outcomes of hybridization. We also note that sampling schemes impact sorting expectations. Here we expected more sorting and lower $\bar{D}$ as we sample from within a single evolving hybrid population. Barrier regions in individuals sampled from across hybrid zones as in Ebdon et al. (2025) should have a higher $\bar{D}$ indicating no introgression.

In conclusion, our overlay of long-term barriers to gene flow and BDMIs in a hybrid population in the wood ant system demonstrates that multilocus BDMIs can act as persistent barriers to gene flow over timescales relevant for species divergence. The enrichment for intronic sequences we find in persistent BDMIs supports the role of regulatory elements in the evolution of species barriers, especially those early in their divergence history. Our study opens the door to future work that should further explore how the biological interaction networks in BDMIs evolve during species divergence and further characterize the genetic function of barrier regions in systems with a range of divergence times.

## Materials and methods

We used 2 complementary methods to identify barriers to gene flow by detecting distinct signals in 2 independent whole-genome datasets: 1 method involved analyzing a set of parental genomes collected from sympatric populations to detect long-term barriers to gene flow, while the other involved sampling a large number of hybrid individuals from a single hybrid population to identify candidate BDMIs.

### Demographic modeling to detect genomic regions of reduced long-term gene flow

First, we analyzed WGS data from 4 diploid workers each of sympatric *F. aquilonia* and *F. polyctena* populations sampled in southern Finland (Fig. 1, further details on samples can be found in Supporting Information) using demographically explicit genome scan, gIMble (Laetsch et al. 2023; v1.0.3). This method identifies long-term barriers as genomic regions of reduced effective migration (*m*_e_) while accounting for demographic history and variation in effective population sizes.

For input to gIMble, BAM files and an unfiltered VCF file of the samples were obtained from Portinha et al. (2022) and were filtered and preprocessed for subsequent gIMble analyses using “gIMble preprocess” with default settings except for minimum coverage (−min_qual 1, −snpgap 2, −min_depth 5, and −max_depth 2 × mean coverage of each BAM). The annotated reference genome from a hybrid *F. aquilonia* × *F. polyctena* haploid male was used throughout (Nouhaud et al. 2022a). gIMble was first run “globally” to identify the best-fit background model of demography for *F. aquilonia* and *F. polyctena* by fitting models with and without long-term effective gene flow. The best-fit model assumed divergence with unidirectional gene flow from *F. aquilonia* to *F. polyctena* (Table S1, Figure S1), an assumption which is consistent with previous findings (Portinha et al. 2022). Then gIMble was run “locally” to identify long-term barriers to gene flow as genomic windows, where a history of reduced *m*_e_ fits better than the global *m*_e_ inferred under the best-fit background model. This was quantified by Δ_B0_ > 0, where the composite likelihood of the reduced gene flow model is higher than the composite likelihood of the global model.

Global and local analyses were limited to intergenic and intronic (noncoding) sequences, excluding repeats, to minimize the direct effects of selection. Furthermore, chromosome 3 (Scaffold 3) was excluded from the global background analysis. In *Formica* species, this chromosome is known as the social chromosome, which contains multiple inversions and genes controlling whether a colony is led by 1 (monogynous) or multiple (polygynous) queens (Brelsford et al. 2020). Recombination is strongly reduced between alleles of monogynous and polygynous colonies, leading to the maintenance of ancestral polymorphisms across *Formica* species which might bias demographic estimates. The data from noncoding and nonrepetitive regions was summarized by the blockwise site frequency spectrum for block length 256 bp with *k*_max_ values of 2. Across analyses, we assumed a constant mutation rate (μ) of 3.5 × 10^−9^ per site per generation, based on estimates available for social insects (Liu et al. 2017), and 2.5 years per generation, following Portinha et al. (2022).

In the local analysis, a fixed number of blocks (-w 2000 -s 400) were grouped into windows with a minimum span of 32 kb (median 41.5 kb, Figure S2). We investigated variation in *N*_e_s (*F. aquilonia*, *F. polyctena*, ancestral population) and *m*_e_ across the windows by searching parameter combinations in a 12 × 12 × 12 × 16 grid (Figure S14). *T* was obtained from the global analysis and fixed as time of species divergence is assumed to be a global event shared across the genome. The local support for long-term barriers was measured using Δ_B0_, where positive Δ_B0_ values indicate reduced gene flow. Uncertainty in parameter estimates was quantified using a parametric bootstrap in msprime (Baumdicker et al. 2022) to estimate the false positive rate (FPR) for Δ_B0_ at a cutoff of 0.05. Specifically, we simulated 100 window-wise datasets under a null model with a fixed *m*_e_ across windows at the background level. For these simulations, we assumed a constant recombination rate based on 1 crossover per female meiosis per chromosome, corresponding to an average map length of 33.3 cM per chromosome as is suitable for haplodiploid species (Hedrick and Parker 1997). Finally, we merged overlapping barrier windows (Δ_B0_ > 0 and FPR ≤ 0.05) and referred to these as barrier regions. Full details on the global and local analyses are available in the Supporting information.

### Imbalanced recombinant haplotype frequency analysis to detect BDMIs

Second, we identified candidate BDMIs by analyzing a second dataset of 286 haploid *F. aquilonia × F. polyctena* male hybrid genomes from a single *F. aquilonia* × *F. polyctena* hybrid population. Hybrid sexuals were collected at 8 time points between years 2004 and 2022, with 30 to 40 individuals sampled at each time point (see Table S4 for samples). To reach the required sample size we have pooled samples over a range of 18 years for the imbalanced recombinant haplotype frequency analysis. This leaves the possibility that early samples may have a signal of BDMIs which have been purged in later samples. However, the effect on the test statistic *X*(2) is that such sites would have higher average values, lowering our ability to detect them. All field samples were collected from W lineage nests during the emergence of sexuals in the Långholmen hybrid population in Hankoniemi, Southern Finland, and stored in 99.5% ETAX ethanol at −4 °C until DNA extraction. Overall, 447 samples were collected and processed for whole-genome sequencing and variant calling. However, only the 286 haploid *F. aquilonia* × *F. polyctena* males were used for the subsequent analysis.

### DNA extraction, library preparation, and sequencing

DNA extractions were performed with Qiagen DNeasy Blood & Tissue Kit (Cat. No./ID: 69504). Prior to DNA extraction, ant tissue samples stored in alcohol were dried and one half from each individual was ground in microcentrifuge tubes using liquid nitrogen and plastic pestles. The lysis step was performed overnight without the optional RNase treatment. Concentrations of the DNA extracts were quantified using Qubit DS DNA HS kit (Q32851;Thermo Fisher Scientific).

DNA libraries were prepared after sample randomization (to avoid batch effects in the temporal dataset) with 150 ng of DNA from each individual DNA extract using New England Biolabs NEBNext Ultra II FS DNA Library Prep Kit for Illumina (E7805L) according to manufacturer's instructions. Indexing of the samples was achieved using NEBNext Multiplex Oligos for Illumina Dual Index Primers Sets 1 and 2 (E7600S and E7780S, respectively). Manufacturer's protocol for use with inputs ≥100 ng DNA was followed and DNA fragmentation was performed with the supplied enzymatic reagent for 11 min at 37 °C and we aimed for a fragment size range of 200 to 450 bp; the recommended 15 min incubation turned out to be too long. AMPure XP SPRI Reagent (A63881) from Beckman Coulter Life Sciences was used for the steps requiring magnetic beads and Invitrogen magnetic stand-96 (AM10027) from Thermo Fisher Scientific was used to precipitate the magnetic beads. We aimed for the final DNA library size range of approximately 320 to 470 bp. Concentrations of the prepared DNA libraries were quantified similarly as the DNA extracts. Quality of the DNA libraries and their average sizes were analyzed with Agilent 5200 Fragment Analyzer and ProSize data analysis software.

Once the quality and the average size of each library passed QC, prepools were prepared from individual DNA library samples, with no more than 23 individual samples per prepool. Individual samples for these prepools were selected based on their average library size from fragment analysis, starting from the shortest and finishing with the longest average fragment size, so that each prepool contained samples of similar size range. These prepool samples were shipped on dry ice to Novogene Corporation Inc. for Illumina sequencing with the Illumina NovaSeq. We aimed for 2 GB of data for haploid males and 4 GB for diploid females.

### Read mapping and variant calling for haploid males in the imbalanced recombinant haplotype frequency analysis

Raw 150 paired-end Illumina reads were trimmed with trimmomatic (Bolger et al. 2014; v0.39) with a minimum length of 80 and a leading and trailing minimum quality of 20. Trimmed reads were mapped to the reference genome using bwa mem (Li 2013; v0.7.17; https://github.com/lh3/bwa) with default parameters. Overlapping reads were clipped with BamUtil (Jun et al. 2015; v1.0.15; https://github.com/statgen/bamUtil). Reads were quality checked with fastQC and visualized/summarized with multiQC (Ewels et al. 2016). Mean duplication and mapping rates across all samples were 13% and 97.6%. Mean coverages for male and female samples were 4.31× and 9.77×, respectively.

Data for 447 individuals (142 females, 305 males) were used for SNP calling with FreeBayes (Garrison and Marth 2012; v1.3.6; https://github.com/freebayes/freebayes) with −limit-coverage 50, -n 4 (limit to 4 best alleles), and −no-population-priors. We normalized reads with vt normalize (Tan et al. 2015) and decomposed variants using vcfuniq from vcflib (Garrison et al. 2022; v1.0.3; https://github.com/vcflib/vcflib). We filtered variants to include biallelic SNPs with a minimum quality of 30, minimum SNP gap of 2, and balanced reads using bcftools (Danecek et al. 2021; v 1.16). Mean depth per individual was calculated with vcftools (Danecek et al. 2011; v0.1.17) −depth and then individuals were filtered to a minimum depth equal to half the mean individual coverage and a max depth equal to 2 times the mean individual coverage with bcftools. Sites in haploid male individuals were filtered to a minimum depth of 3 and female individuals filtered to a minimum depth of 6. Only sites genotyped in at least 60% of samples were retained for subsequent analyses. It is possible that morphologically male sexuals have diploid genomes when the sex determining region of a fertilized egg is homozygous. To identify if our samples contained diploid males, we counted the number of heterozygous sites in each male sample. Altogether we identified 19 male samples whose heterozygosity clustered with diploid female samples and dropped these samples along with female samples from further analysis, resulting in 2,736,443 SNPs in 286 haploid males.

### Identifying and selecting ancestry informative markers

The imbalanced haplotype frequency analysis requires identifying alternatively fixed (or nearly fixed) SNPs which can be polarized with respect to each hybridizing species. We call these ancestry informative markers (AIMs). We filtered the haploid male vcf file with bcftools to remove sites monomorphic for reference or alternate alleles and then converted the vcf file to .geno format with parseVCF.py (https://github.com/simonhmartin/genomics_general). AIM positions were identified using previously published data from Portinha et al. 2022): 10 female *F. aquilonia* (Sample IDs: CBAQ1_1w, CBAQ3_1w, CBAQ2_2w, Lai_1w, Lai_2w, Loa_1w, CF14a_1w, CF4b_1w, CF8b_1w, Pus2_1w) and 10 female *F. polyctena* samples (Sample IDs: CBCH1, CBCH2, CBCH3, CAGa, NAZa, VDa, Att1, Jar6, Lok3) from Europe. As in Li et al. (Li et al. 2022) we defined AIMs as SNPs displaying an allele frequency difference of 95% between the parental species. We filtered the haploid male .geno file to only include positions identified as AIMs, and used a custom R script to convert genotype calls (A/A, T/T, C/C, G/G) to parental ancestry (0 for *F. polyctena* and 1 for *F. aquilonia*) and output .map and .ped files (https://www.cog-genomics.org/plink/1.9/formats) for input to the imbalanced recombinant haplotype (i.e. *X*(2)) analysis. This resulted in a total of 21,283 AIMs across the genome which were used to identify candidate BDMIs (Figure S11).

### Detecting BDMIs in a natural hybrid population

Detecting candidate BDMIs was achieved through an imbalanced recombinant haplotype frequency analysis, which detects negative epistasis between loci in hybrid populations quantified by the *X*(2) statistic from Li et al. (2022). We used biallelic SNPs of known allele ancestry (e.g. F. aquilonia as parent species 0 and F. polyctena as parent species 1). Genotype combinations at 2 loci can then be assigned to single ancestry (00 or 11) or recombinant (01 or 10). BDMIs distort these genotype frequencies into a specific ordering, where one of the recombinant haplotypes is the ancestral and the other is the BDMI so that genotype frequencies follow either g_01_ < g_11_,g_00_ < g_10_ if the 01 allele combination is the BDMI pair or g_10_ < g_11_,g_00_ < g_01_ if the 10 combination is the BDMI pair (see Li et al. 2022 and Li and Lee 2023 for full details). While we do not know the true ancestral or putative BDMI genotype for each loci pair, *X*(2) should be symmetrically distorted for either combination in the case of a true BDMI. We calculated *X*(2) and D′ statistics for all pairwise AIM combinations using the fishStat.permutation R script from Li et al. (2022) (zenodo.org/doi/10.5281/zenodo.6334595).

As it is unlikely that the SNP dataset contains the exact BDMI nucleotide pair, we opted to identify candidate BDMI regions, a reasonable expectation as the signal of selection on BDMI loci distorts linked sites up to 1 to 2Mb away, with the signal being strongest centered on the BDMI loci (Li et al. 2022; Szabo and Cutter 2024). As the identified AIMs were not evenly distributed across the genome (Figure S11), windows used a set number of consecutive AIMs to prevent AIM density affecting inferred incompatibilities. We selected 20 AIMs per window as this was the closest average window size we could get to the observed LD decay of 50Kb while maintaining a feasible number of comparisons. While there was a large variance in window sizes (Table S5), there was not a strong correlation between window size and *X*(2) (Figure S12).

For each window pair we calculated mean *X*(2) and mean D′ between the AIMs in each window, as well as the fraction of AIM comparisons with *X*(2) < −0.005 (Figure S13). With 20 AIMs per window, the number of comparisons between window pairs is 400. We performed 1,000 resamples of an equal number of *X*(2) values (400) from the complete set of AIM comparisons across the genome and calculated the fraction of AIM comparisons with *X*(2) < −0.005 to create a null distribution of how many candidate AIM pairs are expected between 2 windows by chance. We also performed 1,000 random resamples of 40 AIMs across the genome and calculated the number of sites with at least 1 comparison with *X*(2) < −0.005.

We considered a window pair to be a putative BDMI if it met all of the following criteria:

1. The average *X*(2) between the 2 windows is < −0.005.
2. The fraction of AIM comparisons between the 2 windows is greater than the 99th percentile of the bootstrapped fraction distribution.
3. The number of total candidate AIM positions in the 2 windows is greater than the 99th percentile of the bootstrapped distribution.

These criteria assume the true BDMI loci will distort alternative recombinant haplotype frequencies not only at the incompatibility locus, but due to linkage create a similar signal around the BDMI (Li et al. 2022; Szabo and Cutter 2024). By averaging the *X*(2) signal across larger regions we aim to distinguish signal between BDMIs and spurious associations, as spurious associations are unlikely to have linked signals and will average out with combined *X*(2). Meanwhile around true BDMIs a negative *X*(2) value will be maintained when combining comparisons between groups of linked SNPs. With this method we expect to capture loci as candidates which are linked to true incompatibility loci, but not necessarily causal SNPs themselves, and interpret candidate BDMI regions identified as those impacted by a BDMI present within them.

### Quantifying incompatibilities

We quantified the number of incompatibilities simply as the total number of BDMI pairs meeting the BDMI candidate criteria. Based on simulations in Li et al. (2022), the −0.005 cutoff for *X*(2) distinguished neutral and BDMI loci. However, to understand how more filtering affects our results, we present the number of candidate BDMIs pairs for 23 cutoff values (−0.0600, −0.0575, −0.0550, −0.0525, −0.0500, −0.0475, −0.0450, −0.0425, −0.0400, −0.0375, −0.0350, −0.0325, −0.0300, −0.0275, −0.0250, −0.0225, −0.0200, −0.0175, −0.0150, −0.0125, −0.0100, −0.0075, −0.0050), where each cutoff contains the BDMI pairs where the average *X*(2) between windows is less than or equal to the cutoff.

### Calculating multilocus interactions and network features

To calculate the regression of BDMI degree and *m*_e_ we calculated the number of BDMI candidates at each locus with bedtools merge using the -c = 1 and -o = “count” to count the number of BDMI regions at each locus in the BDMI bed file. We then calculated *m*_e_ for each barrier region with bedtools intersecting the BDMI and gIMble bed files with -wa and -wb. We calculated the correlation between BDMI degree and *m*_e_ using the cor.test function from the stats R library (R Core Team 2025). We repeated this procedure for each of the 23 *X*(2) cutoffs.

To check for significance of the observed slopes we bootstrapped *P* values by randomizing *m*_e_ values across the observed BDMI degrees and recalculating the correlation coefficient. We repeated this procedure 1,000 times and compared the observed slope to the bootstrapped distributions of slopes using the ecdf function from the stats R library to calculate *P* values. *P* was determined to be <1/1,000 resamples, or 0.001, when the observed value was greater or less than any observed bootstrap.

To calculate the network measures (Fig. 3e to h, Figures S8 and S9) for the observed BDMI network at each *X*(2) cutoff we used the igraph library in R (Csárdi et al. 2025; R Core Team 2025). We calculated the degree distribution of each network with the function degree and took the max value to be max degree. We calculated the average path with the mean_distance function and we calculated the average betweenness with the betweenness function.

To compare the observed network to a relevant BDMI model for speciation we chose the Orr (Orr 1995) extension of the model. To translate this to a network, we started with 1 node, equivalent to 1 locus where a substitution has occurred between 2 otherwise identical lineages. Nodes were added sequentially, and interacted with previous nodes at rate *P*. We determined *P* as the mean node degree (Eq. 1) of the BDMI network at each *X*(2) cutoff where d bar is the mean degree, and |*V*| is the number of vertices in the graph.


(1) $$P\;=\;\frac{\bar{d}}{|V|-1}$$

As *P* is stochastic, we generated 1,000 Orr networks to generate a null distribution of network measures to compare the BDMI network against. We note that the observed *P* is far higher than the estimates of the probability of interactions between loci as noted in Orr and Turelli (2001). However, our goal is to compare the network topology generated by the model, rather than the number of incompatibilities.

### Overlap between long-term barriers detected with gIMble and BDMIs identified by imbalanced haplotype frequency analysis

To test for an enrichment in overlap between long-term barriers and candidate BDMI regions, we performed a circularization bootstrap similar to Yassin et al. (2016); Nouhaud et al. (2022b) and Ebdon et al. (2025) using a custom R script and bedtools (Quinlan and Hall 2010; v2.31.0). Both gIMble and the *X*(2) analyses rely on SNP data, meaning that the first data point for each analysis does not start at the same position as the start of chromosome. To remove bias in overlap caused by functional features (eg CDS) outside of the range of data available for each analysis, we trimmed all input files to the overlap script to the innermost start and end SNP of either the gIMble or *X*(2) data as appropriate. After this procedure we used the new start and end as the chromosome length for bootstrapping. This procedure should not impact the results much, as >98% of each chromosome length remained.

For bootstrapping, features from each input bed file are merged with bedtools merge (merges region overlaps within files), and then the total observed overlap between the 2 files is calculated in base pairs with bedtools intersect. We then circularize each chromosome, shift the features of the first input bed file by a random percent of each chromosome length, and recalculate the overlap with bedtools intersect. Repeating the circularization 1,000 times creates a bootstrapped null distribution of total overlap values while preserving the distances and clustering of each input feature within chromosomes. A *P* value for each observed value versus the bootstrapped distribution is calculated with the ecdf function from the stats R library.

### Annotation features

We used the same bootstrapping procedure as above to calculate the overlap between gIMble barriers and gene and repeat annotations from Nouhaud et al. (2022a) (doi.org/10.6084/m9.figshare.c.5332442.v1). We filtered the gene annotations file to only include CDS regions. We calculated intronic regions as gene annotation regions—CDS annotation regions (ie we assumed all gene regions were either CDS or intron). The annotation of repeat regions was used as is based on the repeatmodeler2 classifications: including simple repeats, satellite DNA, snRNA, and TEs. We repeated this procedure for each BDMI *X*(2) cutoff.

We divided this analysis into 2 sets. One set including overlapping genome-wide gIMble barriers and BDMIs with CDS, introns, and repeats, and a second set including only regions where gIMble barriers and candidate BDMIs overlapped (long-term BDMIs). To calculate this second set we created a bed file by intersecting the gIMBle bed file with each BDMI *X*(2) cutoff using bedtools intersect (Quinlan and Hall 2010), and bootstrapping the overlap as above.

Since both DNA transposons and LTRs showed significant associations with candidate BDMIs (Figure S16), we retested for associations at the subfamily level for LTRs and DNA transposons with candidate BDMI regions, long-term gIMble barriers, and the gIMble-BDMI intersect regions.

We used the same annotations for identifying the genes present in the intersecting regions of long-term gIMble barriers (i.e. Δ_B0_ > 0) and candidate BDMIs, with the additional restriction of *m*_e_ = 0 for the gIMble barriers with bedtools intersect (Quinlan and Hall 2010). We filtered gene annotations in these regions with gffread (Pertea and Pertea 2020; v0.12.8) to transcripts with > 30 bases (-l 30), containing no in-frame stop codons (-V), adjusting CDS phase to prevent in-frame stop codons (-H), checked single-exon transcripts on the opposite strand (-B), discarded multi-exon mRNAs with any intron with a noncanonical splice site consensus (-N), and have a complete CDS (-J), and output the annotations as amino acid sequences. We then blasted against the NCBI gene database (Brown et al. 2015) with tblastx (Altschul et al. 1990, 1997) keeping the best hits (*e*-value < 0.00001 and >80% identity, Table S9) for each gene.

### Identifying centromeres

To characterize the positions of centromeres, we mapped a 129 bp satellite DNA marker, found in the centromere region in *Formica* ants (Lorite et al. 2004), against the reference genome using BLASTn (Camacho et al. 2009; v2.16.0). If the satellite DNA marker was not found on a chromosome, we identified the centromere regions in the chromosome level assembly of a related species, *F. rufa* (iyForRufa2.1), and then used this information to define the likely chromosome end the centromere belonged to by identifying conserved synteny between the species using RagTag (Alonge et al. 2022).

As a proxy for recombination, we tested whether BDMI and gIMble barriers are closer to centromeric regions than expected by chance. To do this, we calculated the average central position for each centromere for each scaffold which had data. We then took the absolute value of the difference of the center of each BDMI and gIMble region to the centromere. To test if this distribution of distances was smaller than expected by chance, we circularized per chromosome and shifted coordinates by a random percent of each chromosome length and recalculated the distance to centromeres. We compared the observed distribution to the distribution of 1,000 circularized resamples with the Wilcox rank sum nonparametric test. We implemented this with the wilcox.test function in R and the alternative hypothesis “lesser” to test if the observed median is significantly lower than the bootstrapped median.

### HI and barrier sorting

An important aspect of hybrid genome evolution is how the mixed genomic ancestry of an initial hybridization event sorts over time in a population. This involves compatible allele combinations being retained by selection and the purging of deleterious or incompatible combinations. As alleles of shared ancestry have been previously tested together in parent genomes, regions of single ancestry across all individuals in the population begin to emerge (Nouhaud et al. 2022b). To understand how ancestry sorted in the 286 hybrid males we calculated mean $\bar{D}$ across barrier and nonbarrier windows. Using the same 10 *F. aquilonia* and 10 *F. polyctena* samples as in the AIM identification above, we filtered for sites with a difference in ancestry of <0.2 or >0.8 resulting in 65,970 sites across the genome. To prevent an effect of window size, nonbarrier windows (mean 1.6 Mb) were split into multiple windows targeting a mean size equal to the mean of the true gIMble barrier windows (61 kb). We then calculated mean HI for each individual in each region and took the variance across individuals as $\bar{D}$ (Barton and Gale 1993). To summarize the data we took mean $\bar{D}$ across all barrier and nonbarrier windows (Fig. 4c). We compared the observed mean ${\bar{D}}_{\mathrm{barrier}}$ to mean ${\bar{D}}_{\mathrm{non}\text{-}\mathrm{barrier}}$ by bootstrapping null distributions for each using the circular resampling procedure and calculating *P* values as above.

## Supplementary Material

msag063_Supplementary_Data

## Data Availability

Hybrid genomic resequencing read data underlying the X(2) analysis is available at the European Nucleotide Archive (ENA) project PRJEB100734. Code for the gIMble analysis, and X(2) analysis, as well as the underlying data necessary to recreate the figures in this manuscript, are available in the Zenodo repository at https://doi.org/10.5281/zenodo.18035815. The previously published genomic data used in the gIMble analysis, and the reference genome assembly and annotations were obtained from Nouhaud et al. (2022a) in the Figshare repository at https://doi.org/10.6084/m9.figshare.c.5332442 and Nouhaud et al. (2022b) in the ENA project PRJEB51899.
