# Supplementary material for: Genomic incompatibilities are persistent barriers when speciation happens with gene flow in Formica ants
Source: Mol Biol Evol. 2026 Mar 11;43(5):msag063. doi: 10.1093/molbev/msag063 (PMC13143019; doi:10.1093/molbev/msag063)
Supplement: msag063_Supplementary_Data [file msag063_supplementary_data.docx]

**Supplementary Information for**

Genomic incompatibilities are persistent barriers when speciation happens with gene flow in *Formica* ants

Patrick Heidbreder^1,2^*, Noora Poikela^1^, Pierre Nouhaud^5^, Tuomas Puukko^1^, Konrad Lohse^4†^ & Jonna Kulmuni^1,2,3^*^†^

^1^ Organismal and Evolutionary Biology Research Programme, University of Helsinki, Helsinki, 00560, Finland

^2^ Tvärminne Zoological Station, University of Helsinki, Hanko, Finland

^3^ Institute for Biodiversity and Ecosystem Dynamics, University of Amsterdam, Amsterdam, 1090 GE, Netherlands

^4^ Institute of Ecology and Evolution, University of Edinburgh, Edinburgh, EH9 3FL United Kingdom

^5^ CBGP, Univ Montpellier, CIRAD, INRAE, IRD, Institut Agro Montpellier, Montpellier, France

* Corresponding authors
 ^†^ Shared last authorship

* Patrick Heidbreder, Jonna Kulmuni

**Email:** [patrick.heidbreder@helsinki.fi](mailto:patrick.heidbreder@helsinki.fi)

**This PDF file includes:**

Supplementary Figures 1 to 21

Supplementary Tables 1 to 9

**Supporting Figures**


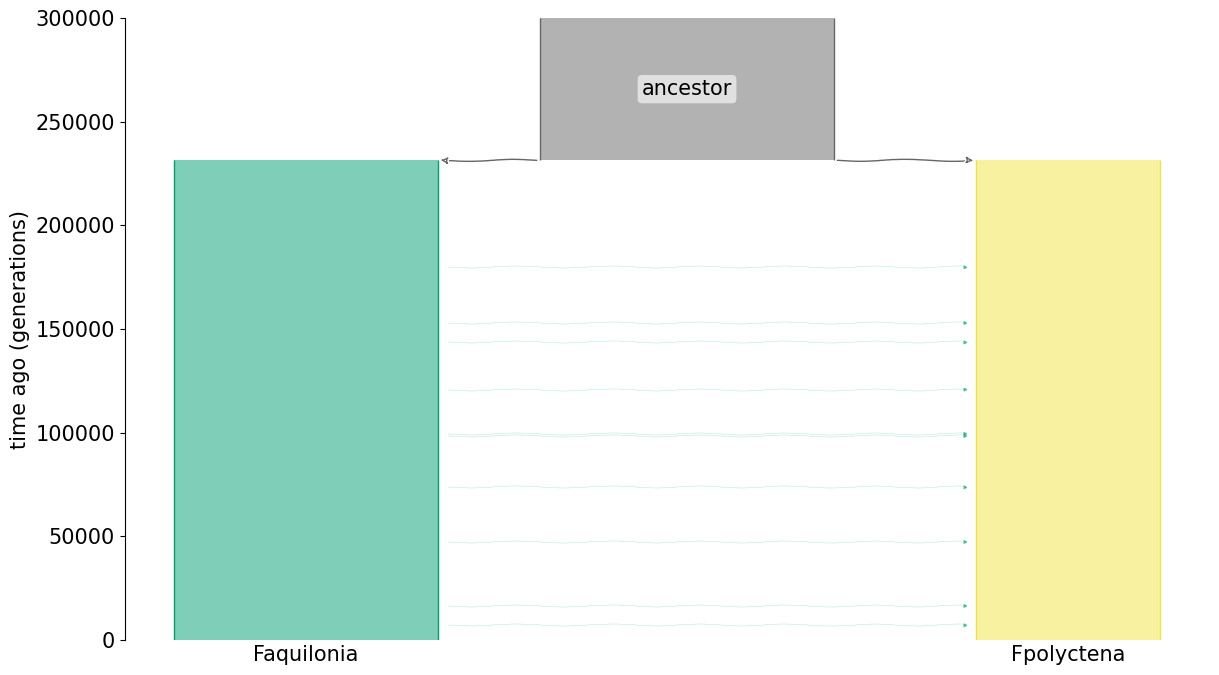


**Figure S1.** Illustration of the best fit demographic model between *F. aquilonia* and *F. polyctena*, IM*_F .aqu→F. pol_*. The block widths indicate relative effective population sizes (*N_e_*) of the ancestral population and its two descendants. Arrows indicate unidirectional gene flow at a rate of 1.5 migrants per generation from *F. aquilonia* to *F. polyctena*. The figure was produced with demesdraw (Gower et al. 2022).


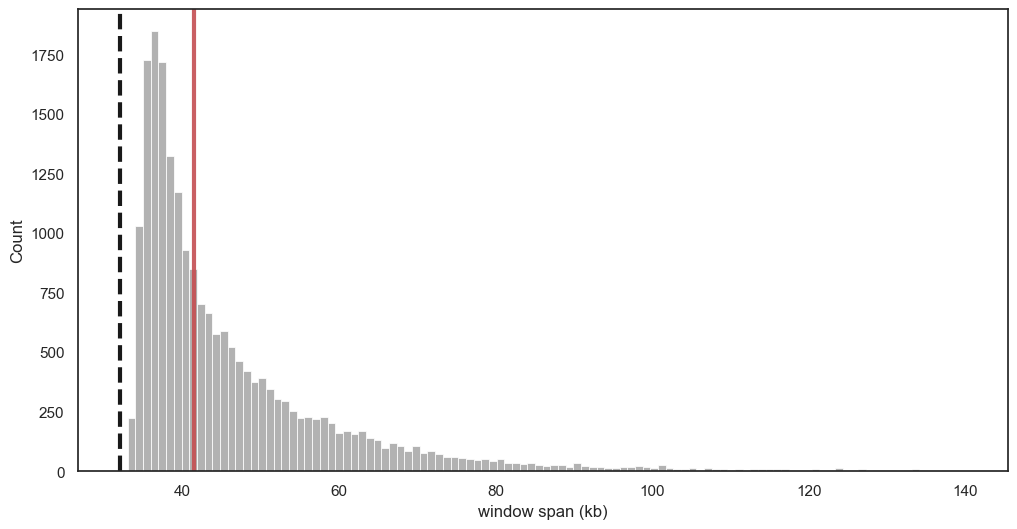


**Figure S2.** The window span distribution of the data set used in gIMble analyses. The genome was analyzed in sliding windows of non-coding sequence. We used a window span of 32 kb (dashed black line), but given that we used only non-coding sequences the window span is greater than that, varying from 33 kb to 756 kb. Median window span is shown with a solid red line (42 kb).


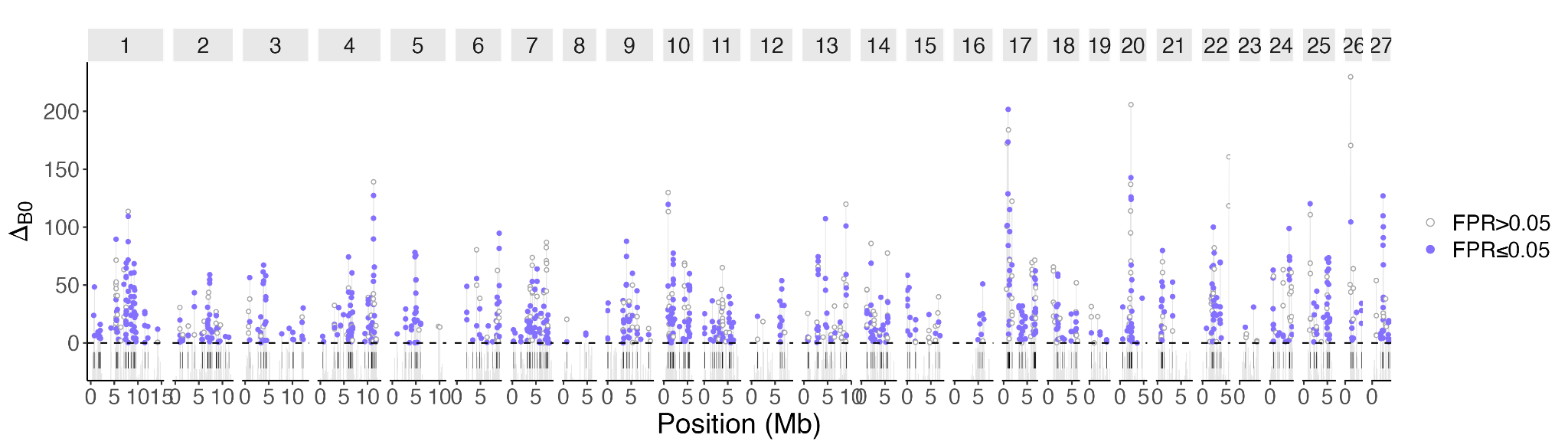


**Figure S3**. Long-term barriers to gene flow between *F. aquilonia* and *F. polyctena* identified by gIMble across all 27 chromosomes. Dots indicate windows with Δ_B0_ > 0, i.e. candidate barriers to gene flow, where a history of reduced *m*_e_ fits better than a model assuming the global estimate. Closed dots indicate significant barriers (false positive rate, FPR ≤ 0.05), and open dots false positives (FPR > 0.05). Significant barrier regions (overlapping windows with Δ_B0_ > 0 and FPR ≤ 0.05) are marked with vertical bars.

**
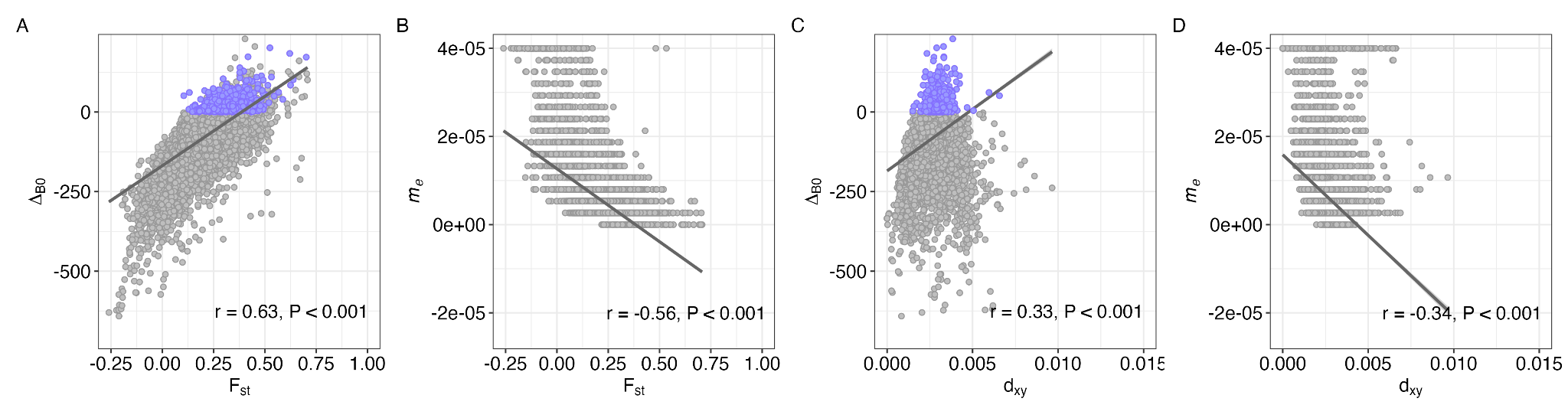
**

**Figure S4**. Window-wise Pearson correlations (r) between metrics of barrier loci (*m*_e_, Δ_B0_) and genetic divergence (*F*_ST_ and *d*_xy_). (A) *F*_ST_ and Δ_B0_, (B) *F*_ST_ and *m*_e_, (C) *d*_xy_ and Δ_B0_, and (D) *d*_xy_ and *m*_e_. Closed dots indicate barriers to gene flow (Δ_B0_ > 0 and FPR ≤ 0.05). Lines represent regression lines.


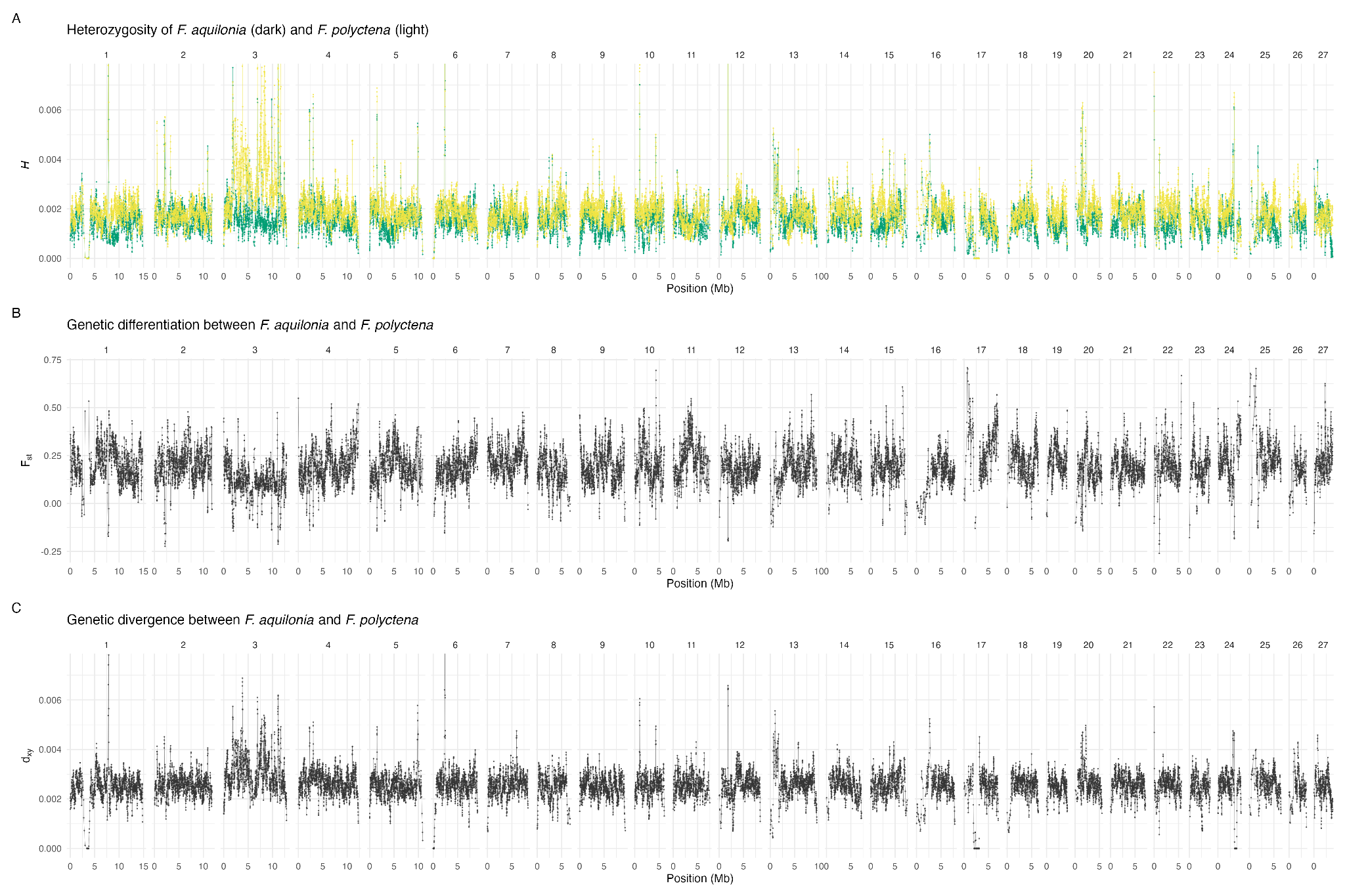


**Figure S5.** Genome-wide (A) heterozygosity (*H*) of *F. aquilonia* and *F. polyctena*, and (B) genetic divergence (*d_yx_*) and (C) genetic differentiation (*F*_ST_) between the species.


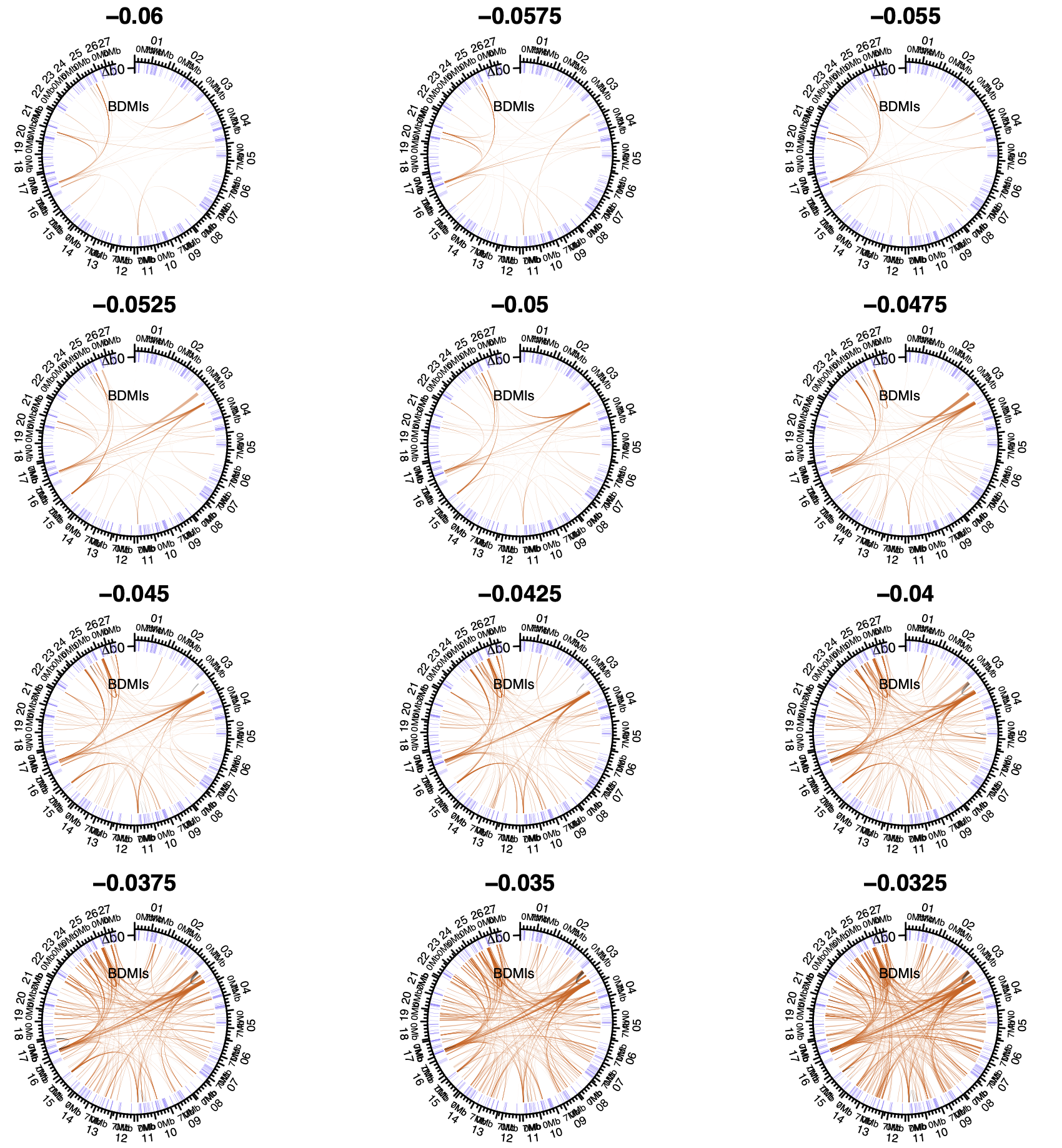


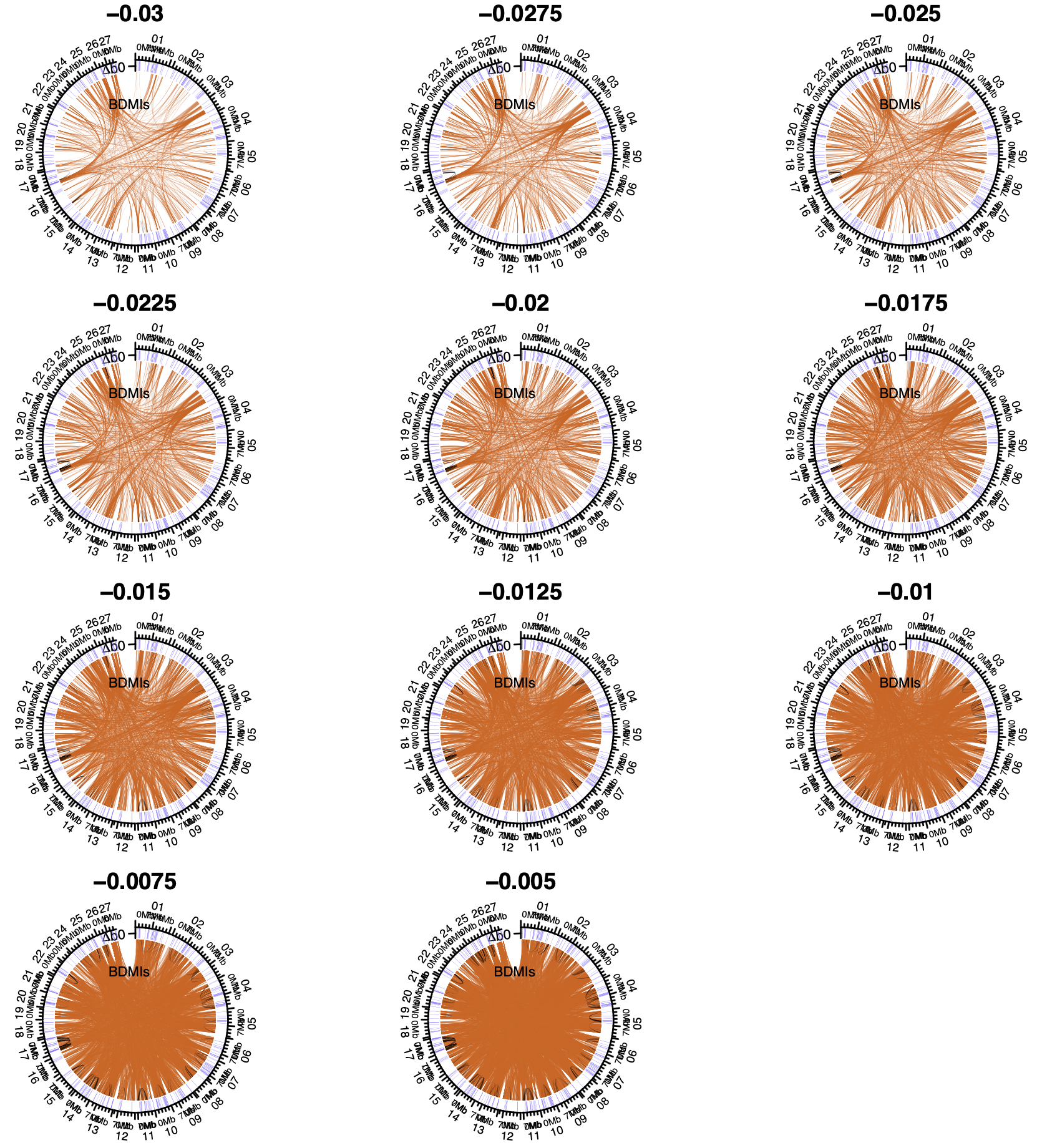


**Figure S6.** Circos plots for all 23 *X*(2) threshold values used in the imbalanced haplotype frequency analysis. Main plot titles are the corresponding *X*(2) cutoff.

**
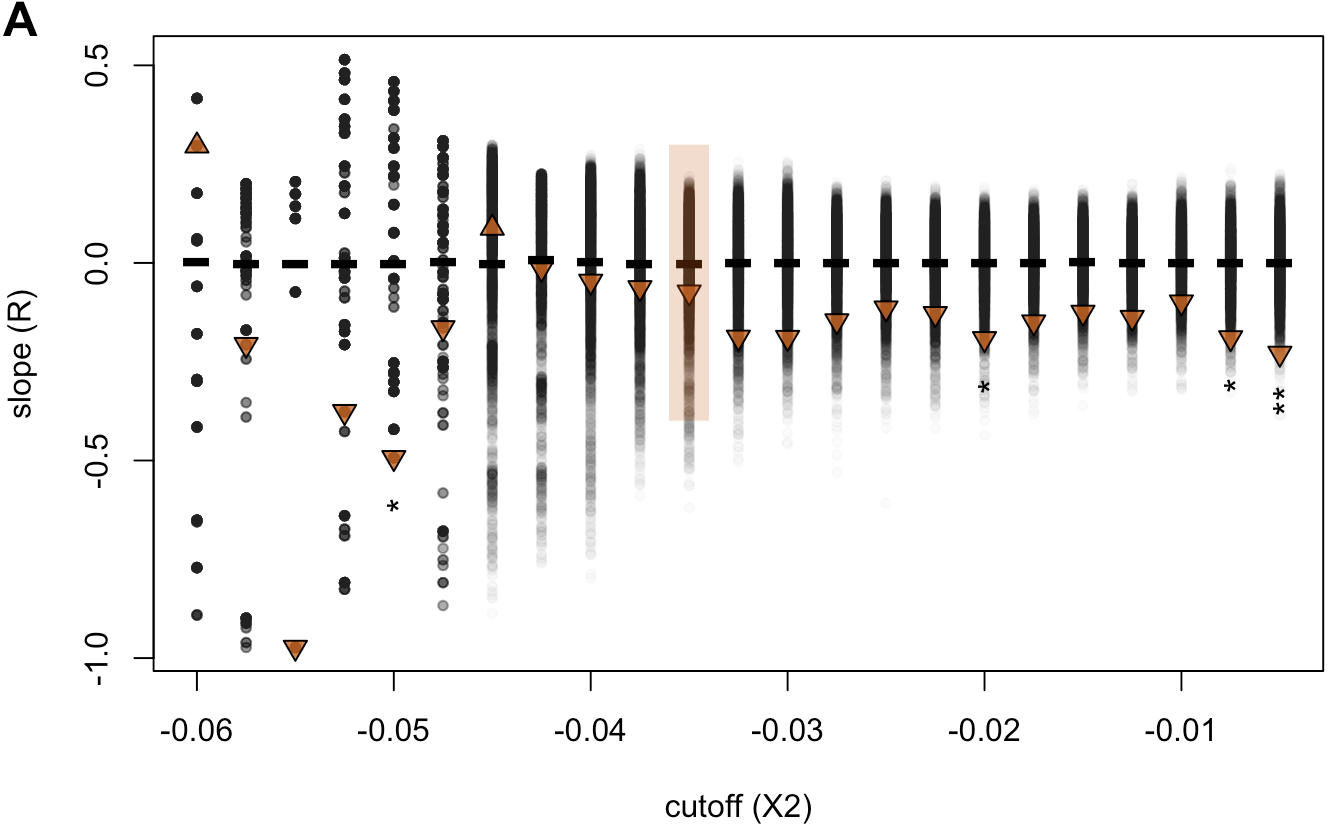
**

**Figure S7.** The effect of removing Scaffold 17 on the correlation between BDMI degree and long-term barrier strength (*m_e_*). Negative correlation remains for X(2) > -0.0425 and significant for X(2) = -0.05, -0.02, -0.0075, and -0.005.


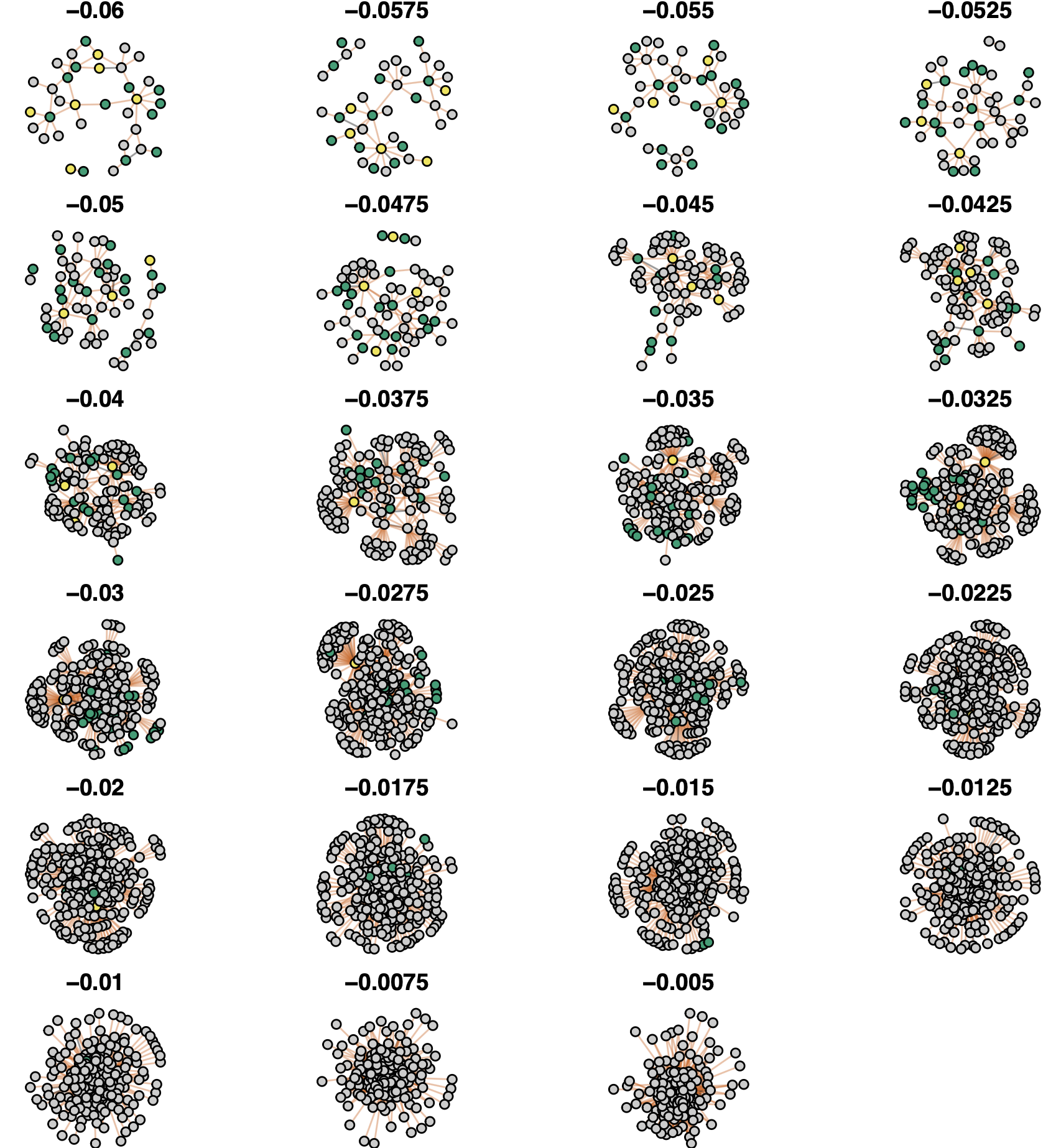


**Figure S8.** Network visualizations for all X(2) thresholds. Nodes are colored based on mean ancestry proportion within the BDMI region the node is associated with. Regions with a hybrid index of <=0.2 are colored green (primarily F. aquilonia ancestry), regions with a hybrid index >= 0.8 are colored yellow (primarily F. polyctena ancestry), regions with intermediated hybrid indices >0.2 & <0.8 are colored grey.


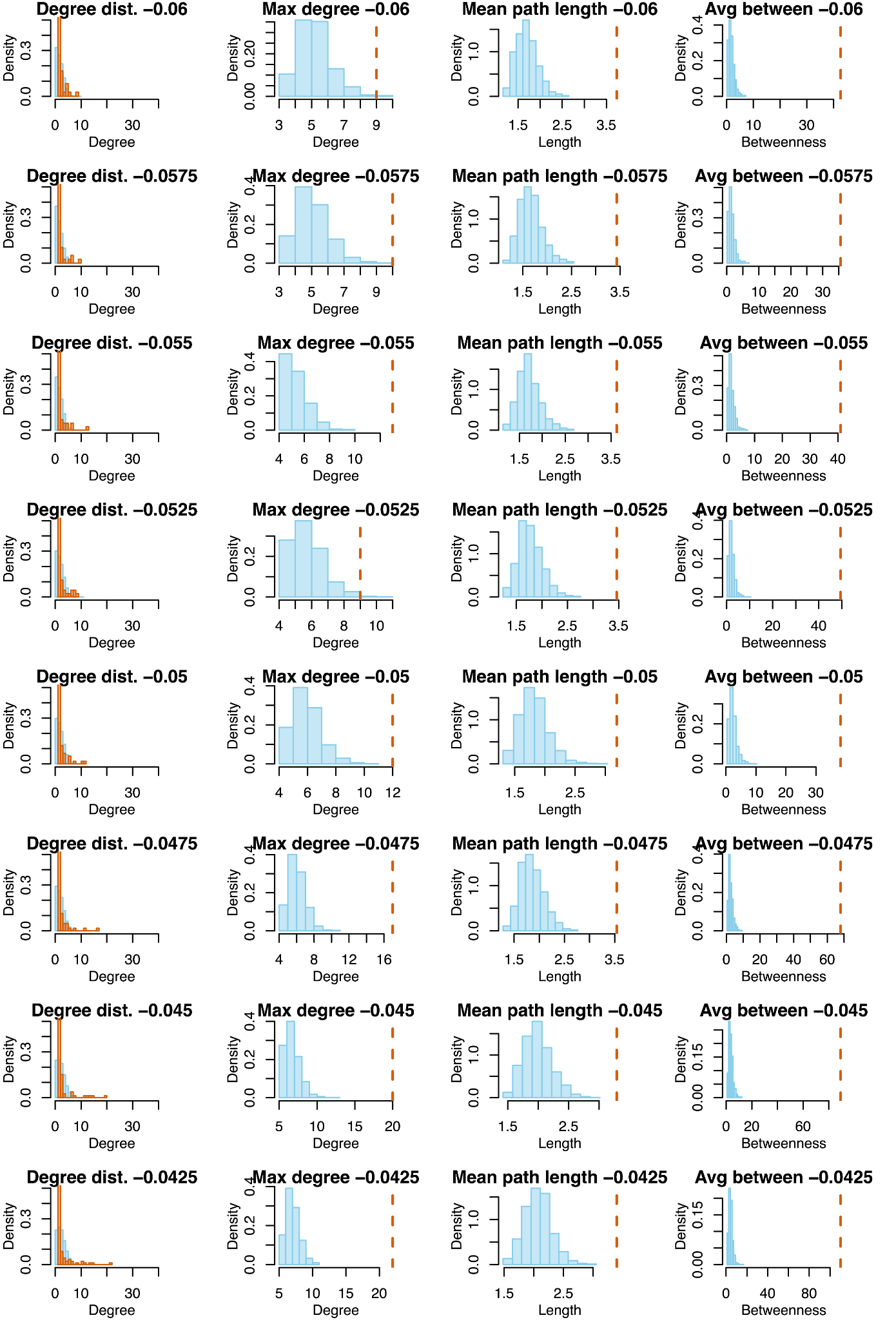


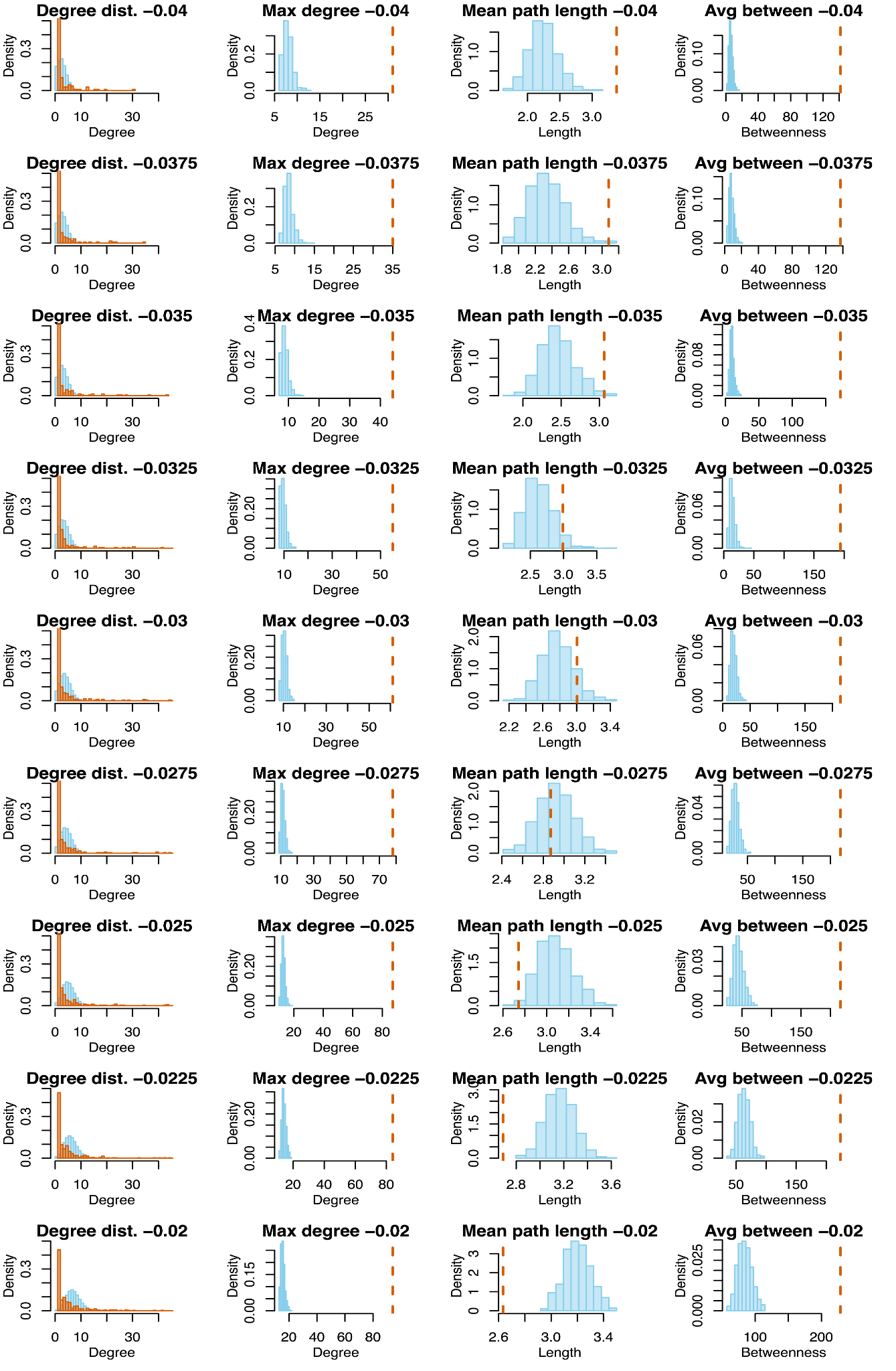


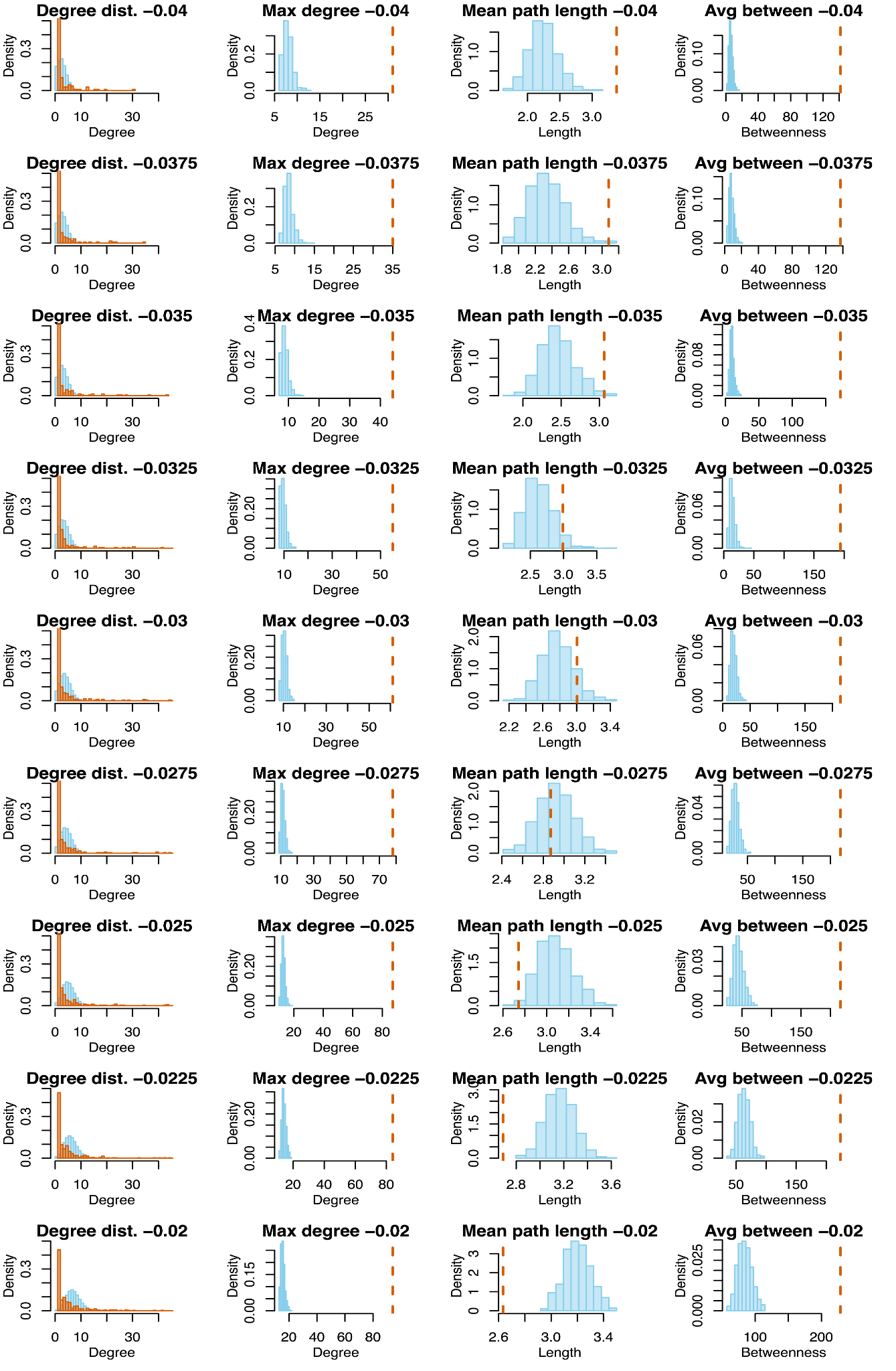


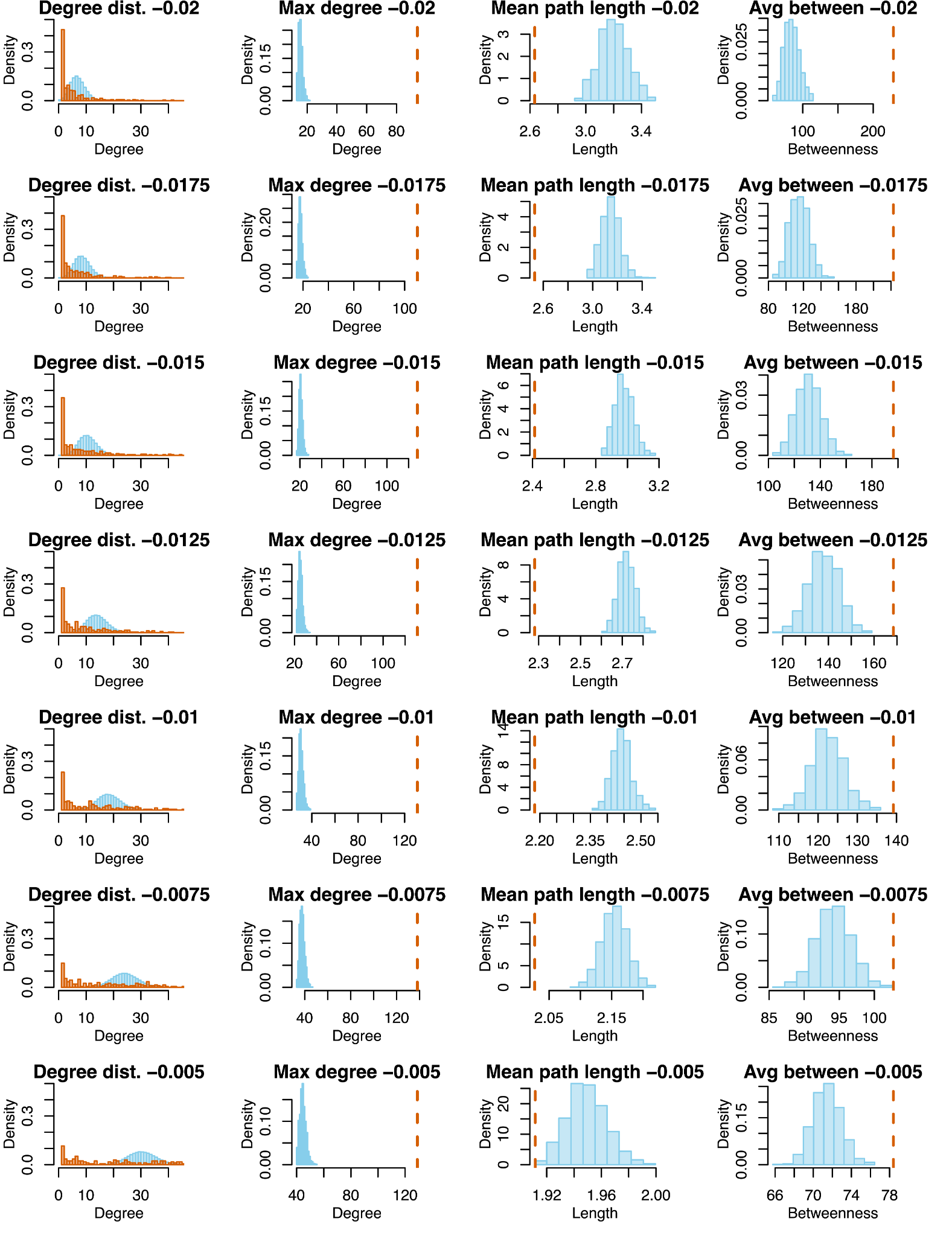


**Figure S9.** Network measure visualizations for all X(2) thresholds. Degree distribution of the observed BDMI network in orange, against the degree distribution generated by the Orr model in blue. Observed BDMI network values (orange dashed lines) versus values from 1000 bootstrapped Orr networks (blue histograms). Max degree is the highest number of edges that are connected to the same node. Mean path length is the average number of edges to travel between two random nodes. Mean betweenness is the average number of network paths through each node.


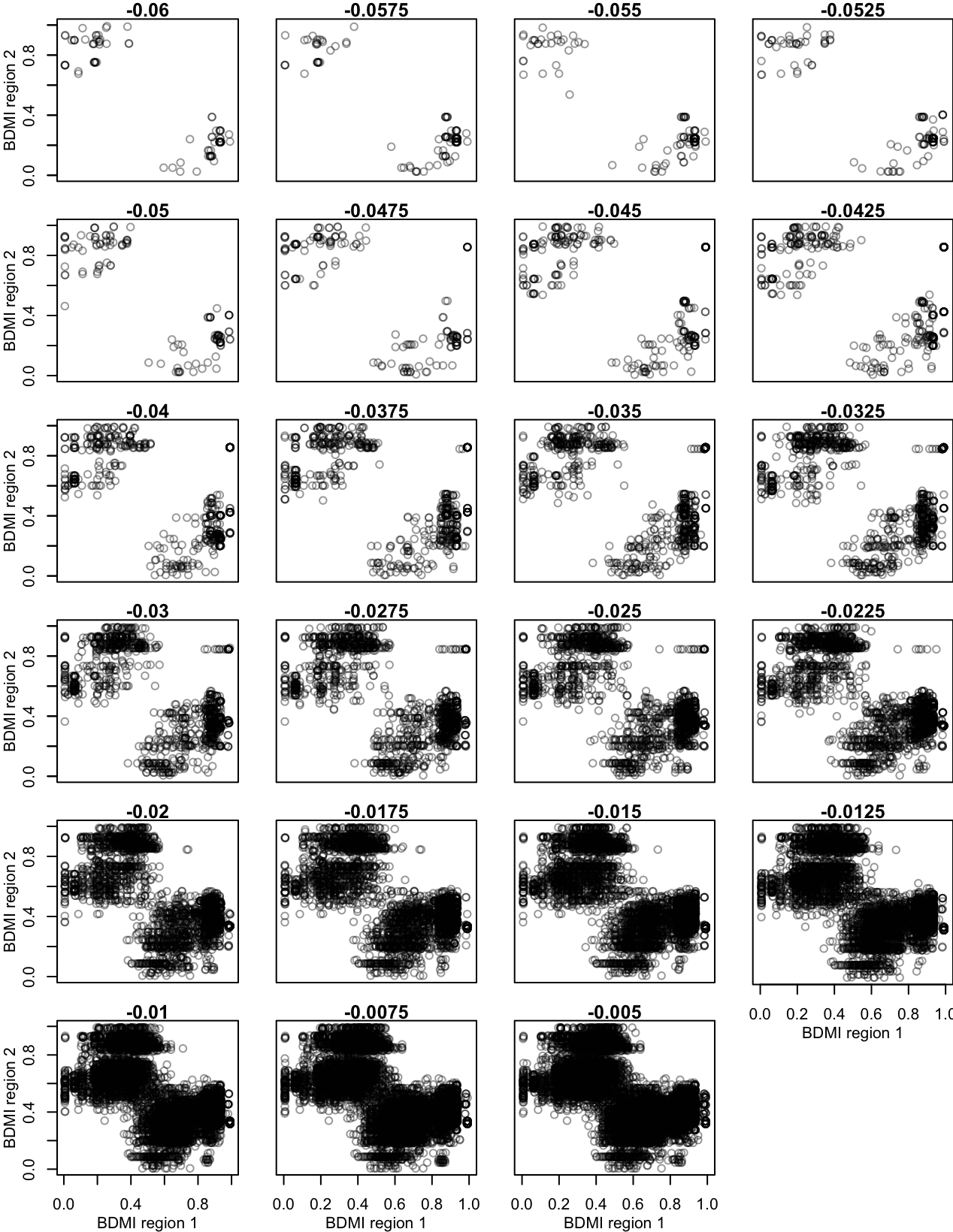


**Figure S10.** Hybrid index for candidate BDMI region pairs. Plot titles are X(2) threshold values. Stricter (more negative) thresholds require stronger ancestry distortion between candidate BDMI windows. X axis is the HI of region one of a BDMI pair and the y axis is the HI of region 2 of the BDMI pair.


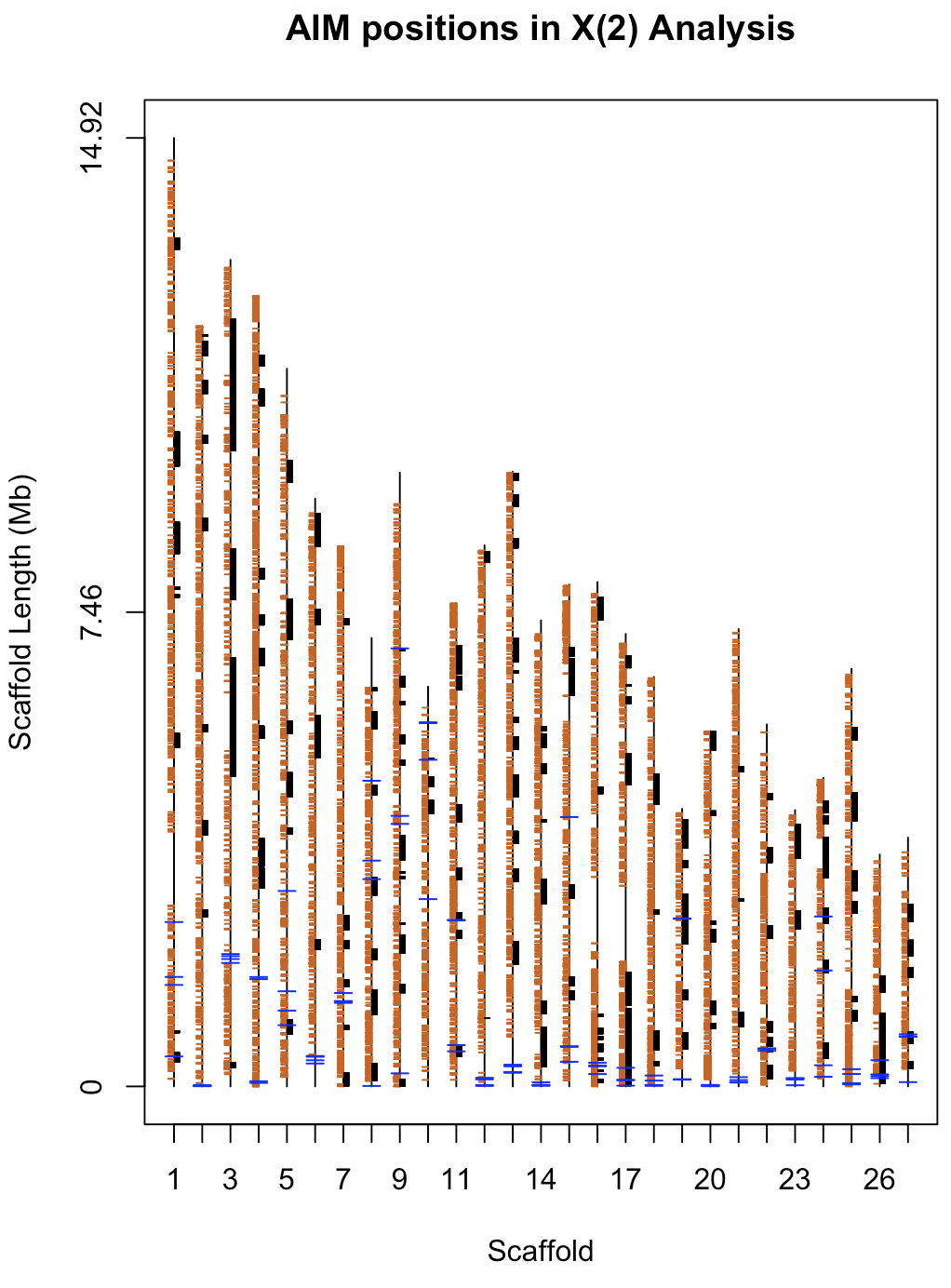


**Figure S11**. Map of 21,283 AIMs used in the unbalanced recombinant haplotype frequency analysis. Black vertical lines are scaffolds, small orange horizontal dashes are AIM positions, larger blue horizontal dashes are reference assembly gaps, black rectangles are candidate BDMI regions at the X(2) < -0.035 threshold.


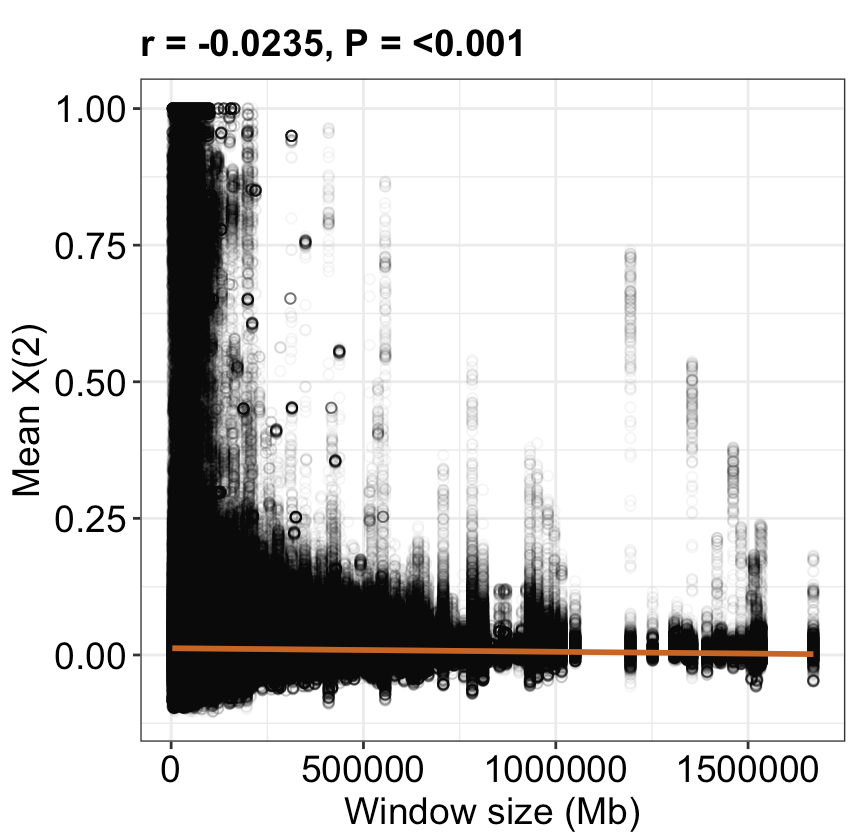


**Figure S12.** Window size effect on *X*(2) values in the *X*(2) analysis. Window sizes vary due to using an equal number of AIMs per window. There is a weak but significant correlation between window size and mean X(2) values.


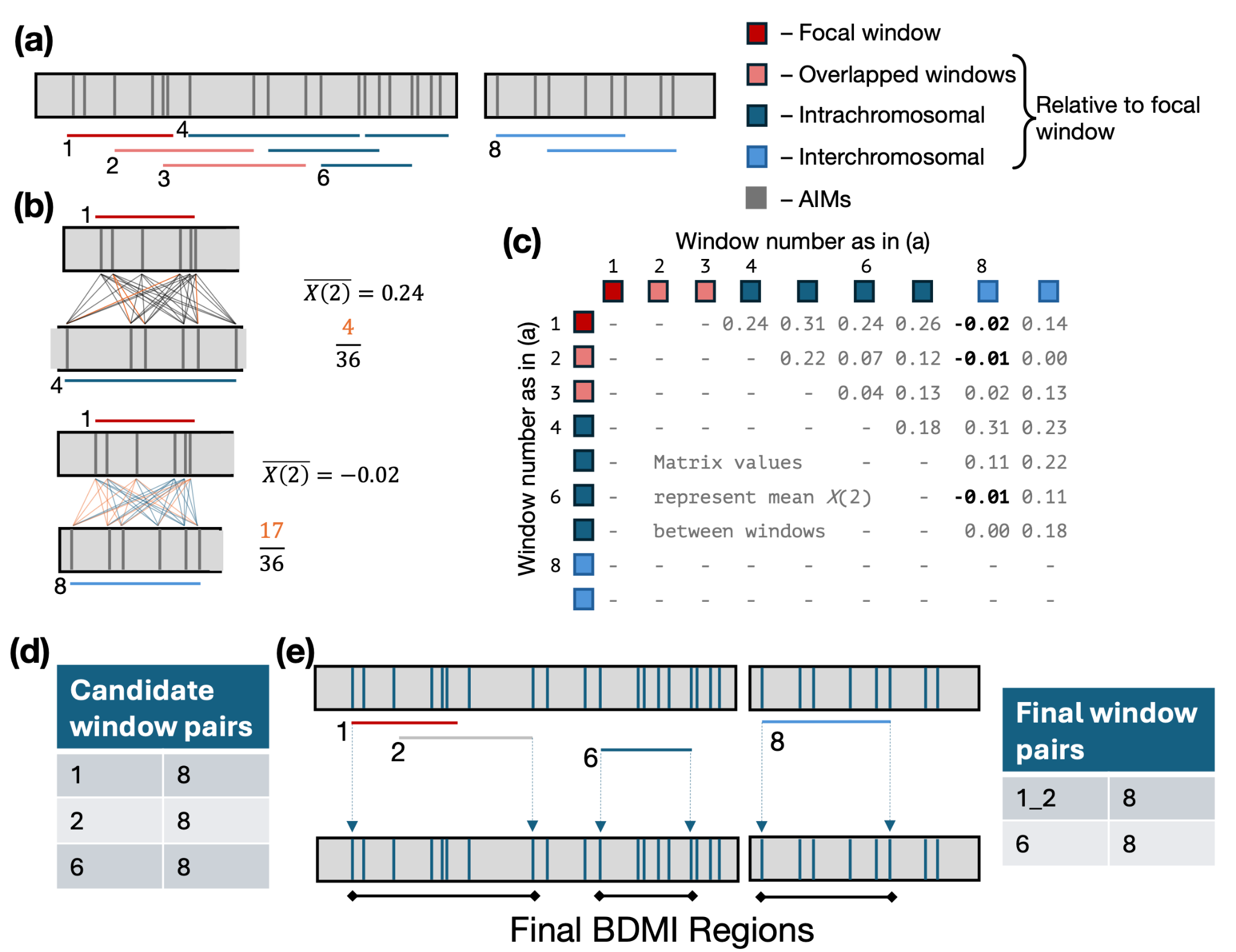


**Figure S13.** Identifying candidate BDMI Regions. (a) Windows contain equal numbers of AIMs and overlap by a step size of 5%. Real windows in the analysis contain 20 AIMs and shift in steps of 4, with the last window containing 18-22 AIMs (20 ± step size / 2). For simple representation windows in this figure contain 6 aims each. (b) Example cartoon comparison between focal window 1 (red line) and an intrachromosomal window 4 (dark blue line) and between focal window 1 (red line) and interchromosomal window 8 (light blue line). The mean X(2) between all AIMs in each window is calculated (e.g 0.24 for windows 1 vs. 4 and -0.02 for window 1 vs. 8), as well as the fraction of AIM comparisons with X(2) < -0.005 (e.g. 4 out of the 36 comparison between windows 1 and 4 have *X*(2) < -0.005, while 17/36 do between windows 1 and 8). (c) Comparisons are pairwise between all non-overlapping windows (i.e. window 1 shares AIMs with windows 2 and 3, so no comparison is made). Cartoon matrix of mean *X*(2) between window pairs. (d) Candidate window pairs are those with a signal of a BDMI as indicated by both negative mean X(2) (bolded values in (c)) and a fraction of comparisons which is greater than expected by chance. (e) Candidate window pairs which share AIMs (e.g. pairs 1 & 8 and 2 & 8) are merged to create the candidate BDMI regions.


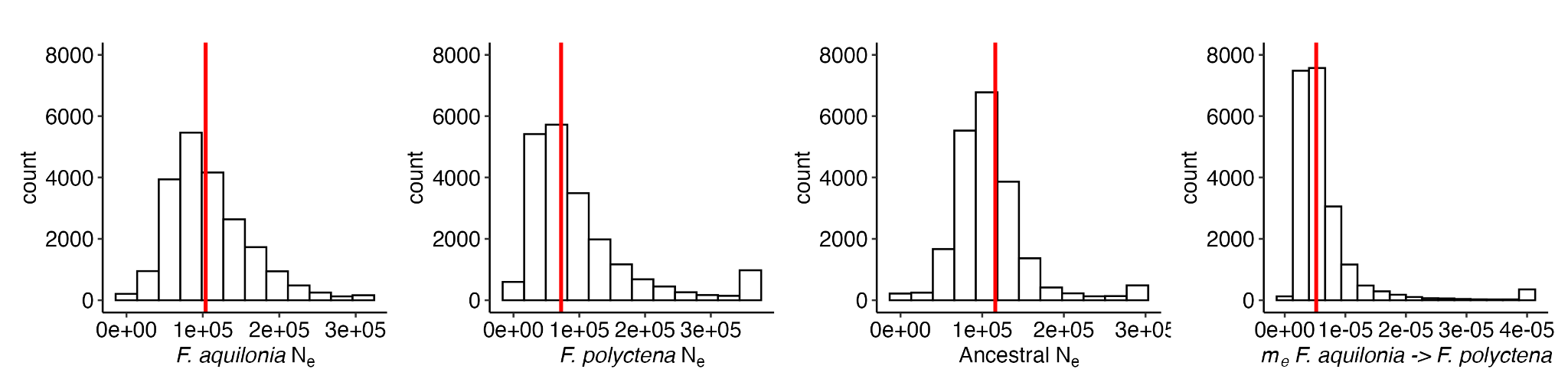


**Figure S14.** Variation in the effective population sizes (*Ne*_e_) of *F. aquilonia*, *F. polyctena* and their shared ancestral population, and variation in the migration rates (*m*_e_) from *F. aquilonia* to *F. polyctena* across sliding windows. The red vertical lines indicate estimates under the global model (Table S1). The number of bins in each distribution corresponds to points in the 12 × 12 × 12 × 16 parameter grid used for inference.


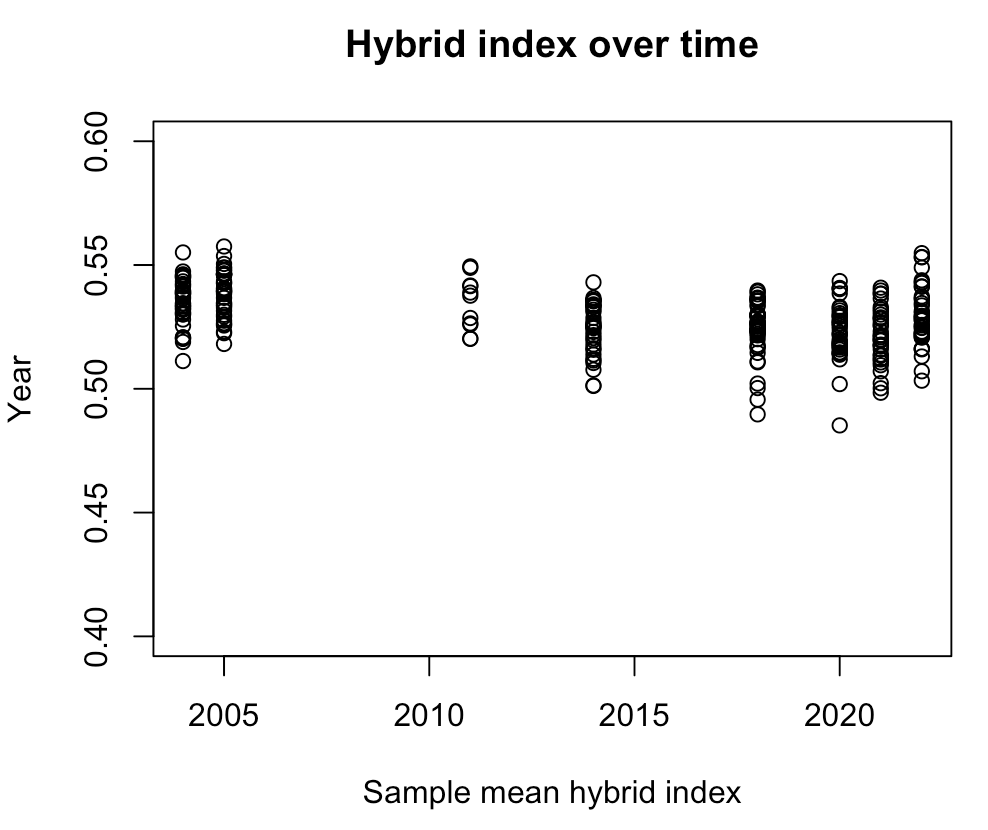
x

Figure S15. Mean hybrid index (HI) for all haploid male samples used in the *X*(2) analysis over time.


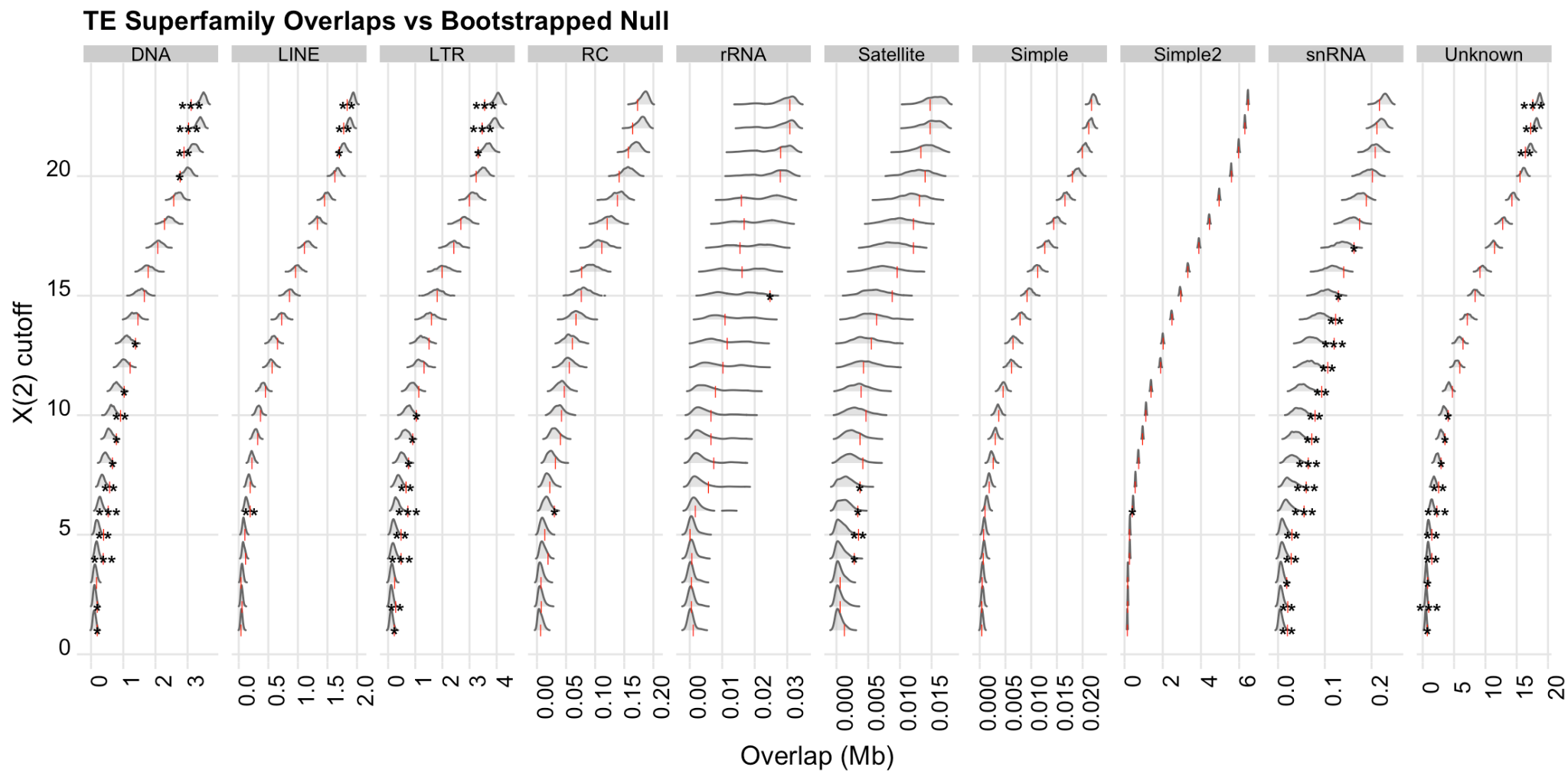


Figure S16. Overlap bootstrapping for repetitive elements and candidate BDMI regions. DNA = DNA transposons, LTR = Long terminal repeat retrotransposons, RC = Rolling circle (Helitrons), rRNA = ribosomal RNA, simple repeats, simple2 repeats includes variable number tandem repeats of the form (NN)_n_ to (NNNNNN)_n_, and Unknown TEs are TEs not classified in the Nouhaud et al. 2022 annotation.


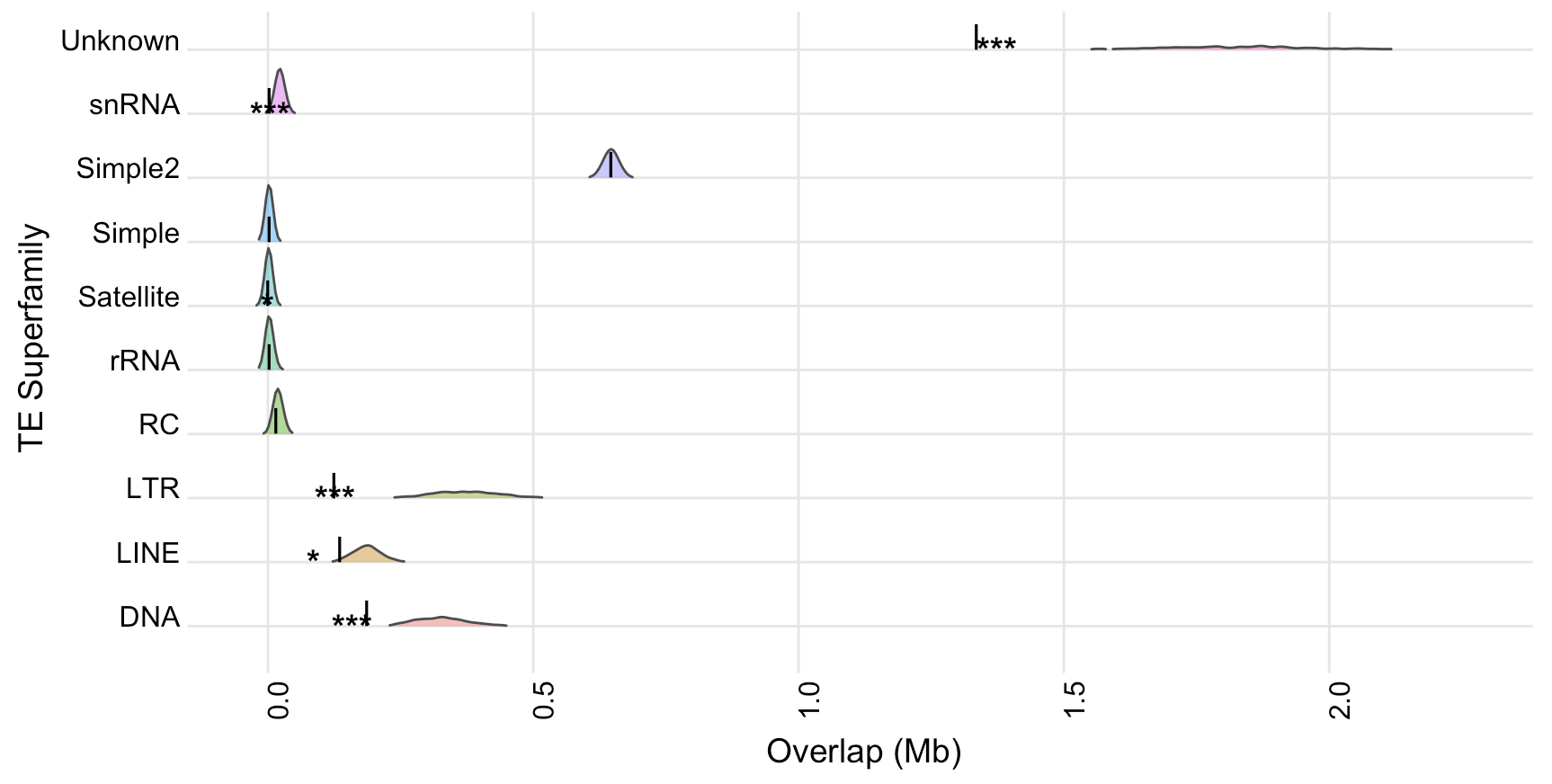


Figure S17. Overlap bootstrapping for repetitive elements and gIMble barrier regions. Figure S16. Overlap bootstrapping for repetitive elements and candidate BDMI regions. DNA = DNA transposons, LTR = Long terminal repeat retrotransposons, RC = Rolling circle (Helitrons), rRNA = ribosomal RNA, simple repeats, simple2 repeats includes variable number tandem repeats of the form (NN)_n_ to (NNNNNN)_n_, and Unknown TEs are TEs not classified in the Nouhaud et al. 2022 annotation.


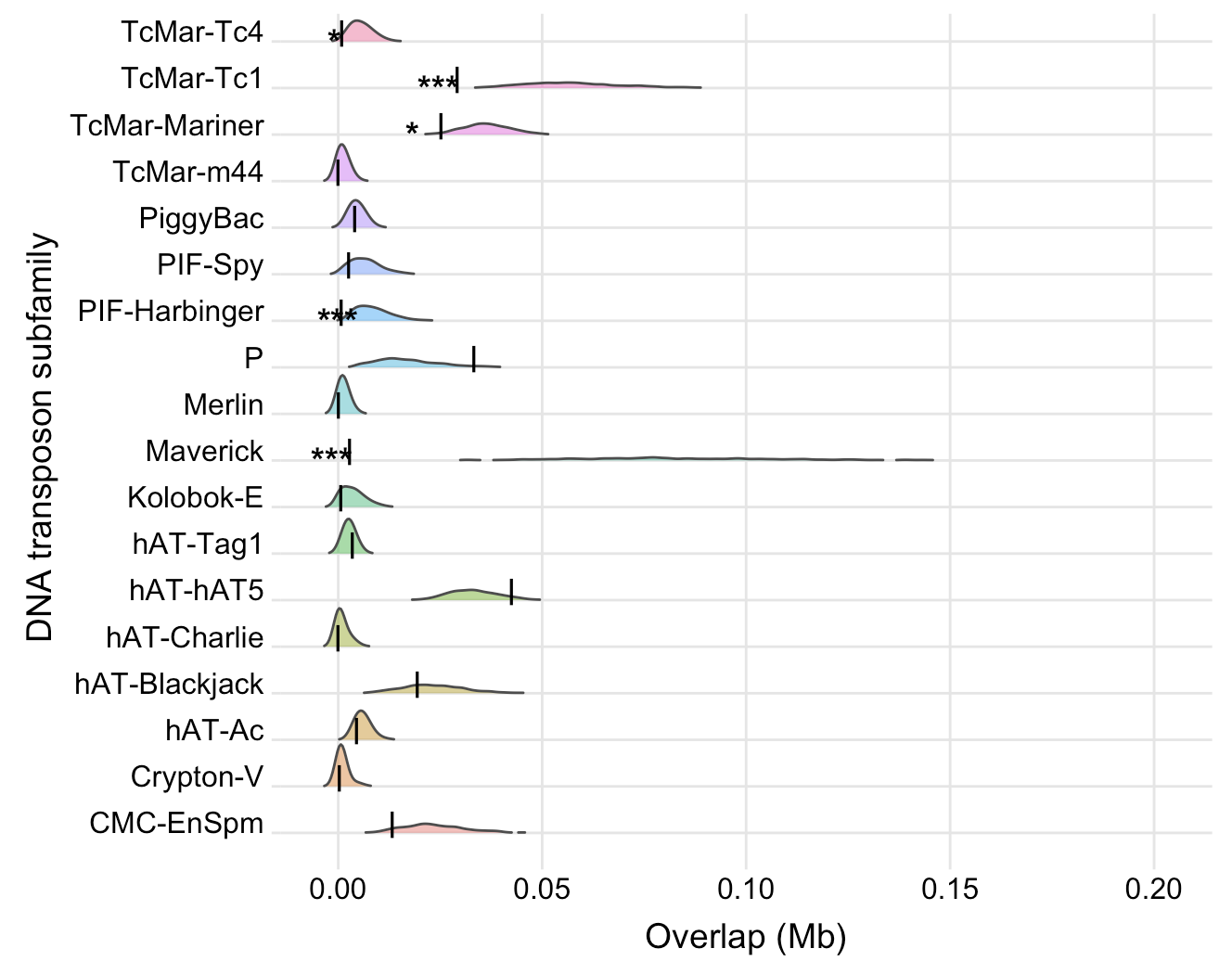


Figure S18. Overlap for gIMble barriers and DNA transposon subfamilies


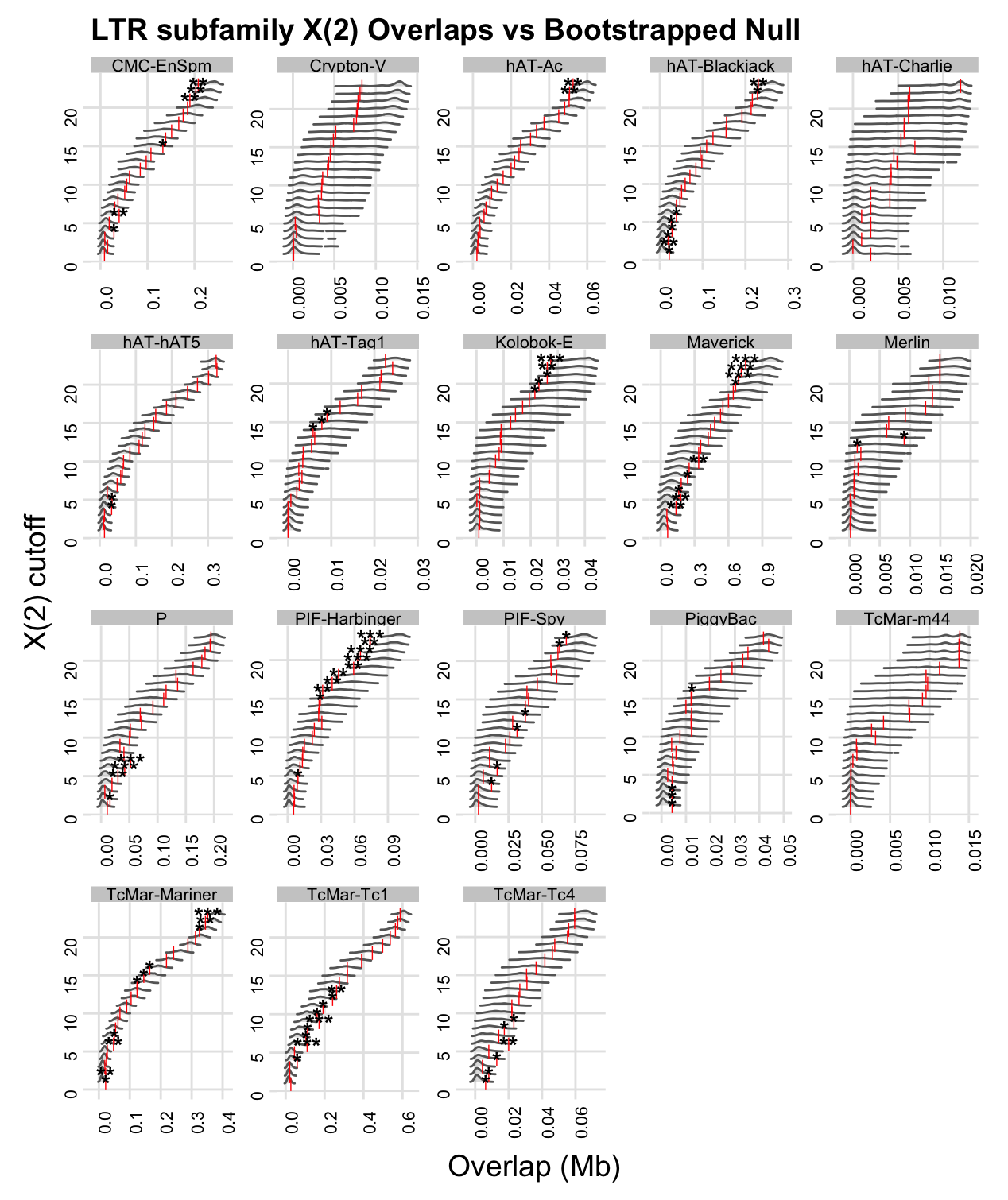


Figure S19. Overlap for candidate BDMIs and DNA transposon subfamilies


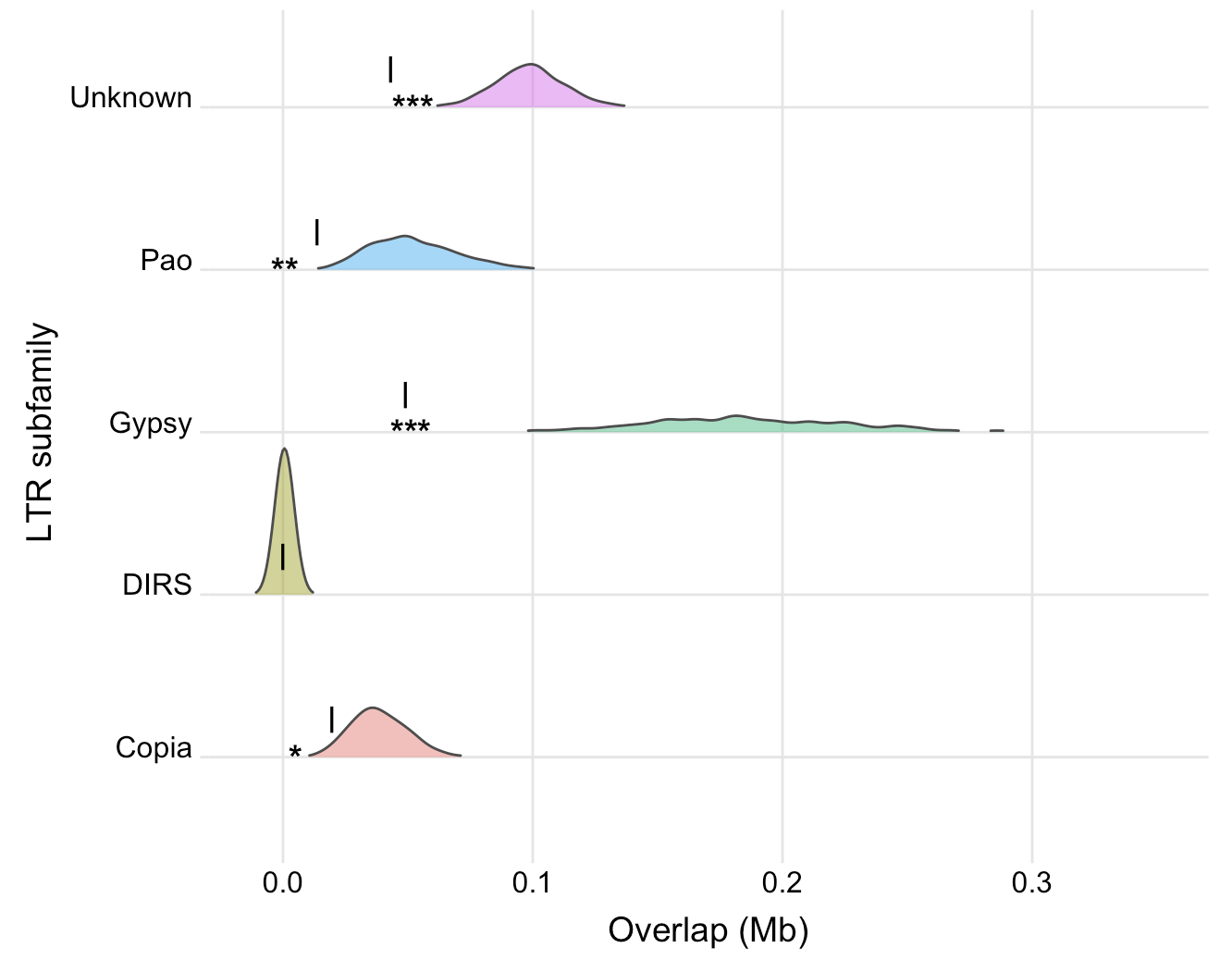


Figure S20. Overlap for gIMble barriers and LTR retrotransposon subfamilies


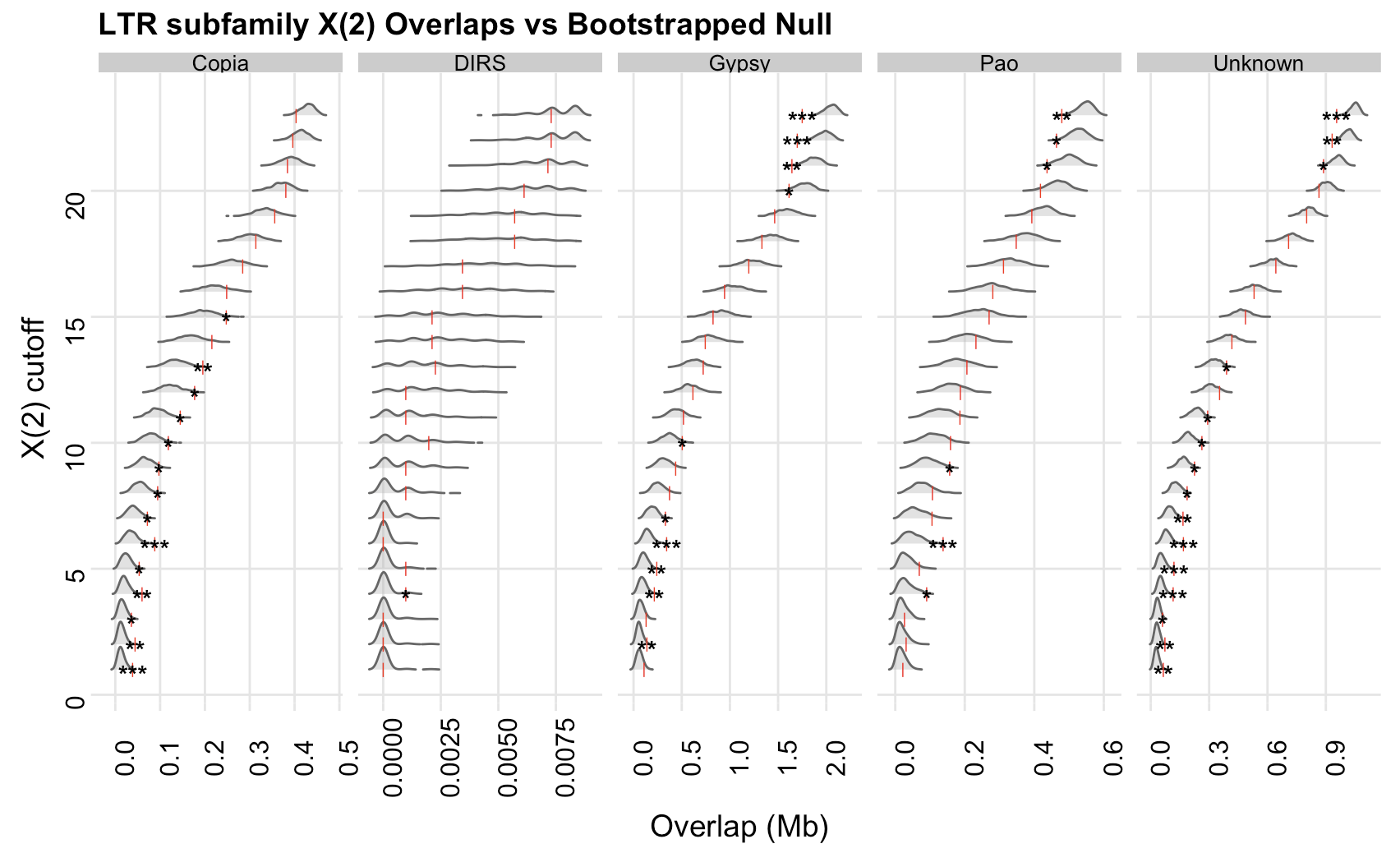


Figure S21. Overlap for candidate BDMIs and LTR retrotransposon subfamilies

**Supporting Tables**

**Table S1.** Global estimates of the demographic history of *F. aquilonia* and *F. polyctena* inferred using gIMble under models of strict divergence (DIV), migration without divergence (MIG), and isolation with migration (IM). Gray shading indicates the best-fit model. Parameter estimates are scaled in absolute units, i.e. number of individuals (*N_e_*) and years (*T;* converted from generations to years assuming 2.5 years per generation). Migration rate (*m*) estimates correspond to M (=4*N_e_m_e_*) individuals per generation (forwards in time).

| **Model** | **Ancestral *Ne*** | ***F. aqui Ne*** | ***F. pol Ne*** | ***T* (years)** | ***m* (M)** | **ΔlnCL** |
| --- | --- | --- | --- | --- | --- | --- |
| DIV | 1.36E+05 | 7.57E+04 | 1.20E+05 | 2.19E+05 | - | -17872 |
| MIG *F. aqui --> F. pol* | - | 1.07E+05 | 5.81E+04 | - | 6.93E-06 (1.61) | -12682 |
| MIG *F. pol --> F. aqui* | - | 3.25E+04 | 1.27E+05 | - | 9.16E-06 (1.19) | -24912 |
| IM *F. aqui --> F. pol* | 1.16E+05 | 1.04E+05 | 7.24E+04 | 5.78E+05 | 5.19E-06 (1.50) | 0 |
| IM *F. pol --> F. aqui* | 1.12E+05 | 4.30E+04 | 1.45E+05 | 5.36E+05 | 6.28E-06 (1.08) | -1111 |

**Table S2.** Chi-Square test results assessing whether the number of barrier windows on each chromosome matches the genome-wide occurrence rate (3.36%) or shows enrichment. The Chi-Square test statistic (X²) and corresponding p-values are listed for each chromosome. No significant enrichment of barriers was observed on any chromosome.

| **Chromosome** | **Observed barrier fraction (%)** | **X^2^** | **df** | **p-value** |
| --- | --- | --- | --- | --- |
| 1 | 5.1 | 0.69 | 1 | 0.406 |
| 2 | 3.1 | 0.00 | 1 | 1.000 |
| 3 | 2.1 | 0.44 | 1 | 0.509 |
| 4 | 4.7 | 0.35 | 1 | 0.556 |
| 5 | 2.0 | 0.37 | 1 | 0.544 |
| 6 | 2.6 | 0.03 | 1 | 0.860 |
| 7 | 5.1 | 0.32 | 1 | 0.569 |
| 8 | 0.5 | 1.52 | 1 | 0.218 |
| 9 | 3.1 | 0.00 | 1 | 1.000 |
| 10 | 7.6 | 1.59 | 1 | 0.207 |
| 11 | 4.2 | 0.00 | 1 | 0.951 |
| 12 | 1.5 | 0.59 | 1 | 0.442 |
| 13 | 3.4 | 0.00 | 1 | 1.000 |
| 14 | 4.7 | 0.07 | 1 | 0.785 |
| 15 | 2.1 | 0.08 | 1 | 0.772 |
| 16 | 1.2 | 0.43 | 1 | 0.510 |
| 17 | 9.7 | 2.75 | 1 | 0.098 |
| 18 | 3.7 | 0.00 | 1 | 1.000 |
| 19 | 1.1 | 0.35 | 1 | 0.554 |
| 20 | 4.5 | 0.00 | 1 | 0.944 |
| 21 | 2.3 | 0.06 | 1 | 0.812 |
| 22 | 4.9 | 0.05 | 1 | 0.817 |
| 23 | 0.5 | 0.77 | 1 | 0.381 |
| 24 | 4.7 | 0.01 | 1 | 0.913 |
| 25 | 6.0 | 0.31 | 1 | 0.576 |
| 26 | 4.2 | 0.00 | 1 | 1.000 |
| 27 | 6.2 | 0.23 | 1 | 0.631 |

**Table S3.** Mean genetic diversity (π) and heterozygosity (*H*) within *F. aquilonia* and *F. polyctena*, and mean genetic divergence (*d*_xy_) and genetic differentiation (*F*_ST_) between the species.

| ***F. aquilonia* π** | ***F. polyctena* π** | ***F. aquilonia* *H*** | ***F. polyctena* *H*** | ***d*_xy_** | ***F*_ST_** |
| --- | --- | --- | --- | --- | --- |
| 0.00217 | 0.00272 | 0.00201 | 0.00251 | 0.00327 | 0.18356 |

**Table S4.** Metadata for samples used in SNP calling for the X(2) analysis. Sample and ID are unique sample identifiers. Sex is the morphological identity of the sample sex. Nest is the name of the nest the sample was collected from. Dev_stage is the stage of development at time of DNA extraction (pupa, young_adult/sexual, adult). Year is the year the sample was collected. Ploidy is the ploidy level of the sample (1 = haploid, 2 = diploid). Caste is the sample social caste (m = male drone, w = female worker, q = female queen). avg_DP is the mean sequencing depth for the sample (decimal marker is ,). Map_rate is the percent of reads mapped.

| sample | ID | sex | nest | dev_stage | year | ploidy | caste | avg_DP | map_rate |
| --- | --- | --- | --- | --- | --- | --- | --- | --- | --- |
| a10 | m012-2011 | m | Katajikko | NA | 2011 | 1 | m | 4,272 | 984 |
| a100 | m001-2005 | m | FA6 | adult | 2005 | 1 | m | 3,117 | 988 |
| a101 | m025-2004 | m | Nest2 | adult | 2004 | 1 | m | 5,321 | 989 |
| a102 | m002-2005 | m | FA6 | adult | 2005 | 1 | m | 5,749 | 991 |
| a103 | m006-2014 | m | FA15 | NA | 2014 | 1 | m | 4,248 | 989 |
| a104 | m026-2004 | m | Nest2 | adult | 2004 | 1 | m | 5,250 | 989 |
| a105 | m001-2018 | m | FA18 | pupa | 2018 | 1 | m | 4,744 | 987 |
| a106 | m027-2004 | m | Nest2 | adult | 2004 | 1 | m | 3,875 | 990 |
| a107 | m007-2014 | m | FA15 | NA | 2014 | 1 | m | 3,478 | 990 |
| a108 | m002-2018 | m | FA24 | adult | 2018 | 1 | m | 5,515 | 988 |
| a109 | m028-2004 | m | Nest2 | adult | 2004 | 1 | m | 4,684 | 989 |
| a110 | m003-2005 | m | FA6 | adult | 2005 | 1 | m | 4,684 | 985 |
| a111 | m029-2004 | m | Nest2 | adult | 2004 | 1 | m | 4,429 | 987 |
| a112 | m004-2005 | m | FA10 | adult | 2005 | 1 | m | 5,017 | 856 |
| a113 | m030-2004 | m | Nest4 | adult | 2004 | 1 | m | 4,703 | 987 |
| a115 | m005-2005 | m | FA6 | adult | 2005 | 1 | m | 4,354 | 985 |
| a119 | m012-2014 | m | FA17 | NA | 2014 | 1 | m | 3,029 | 844 |
| a12 | m017-2014 | m | FA17 | NA | 2014 | 1 | m | 3,507 | 989 |
| a120 | m003-2018 | m | FA15 | pupa | 2018 | 1 | m | 4,576 | 989 |
| a121 | m004-2018 | m | FA24 | adult | 2018 | 1 | m | 5,163 | 984 |
| a122 | m031-2004 | m | Nest4 | adult | 2004 | 1 | m | 5,133 | 985 |
| a123 | m014-2014 | m | FA15 | NA | 2014 | 1 | m | 3,895 | 989 |
| a124 | m005-2018 | m | FA18 | pupa | 2018 | 1 | m | 3,963 | 984 |
| a125 | m006-2005 | m | FA6 | adult | 2005 | 1 | m | 5,958 | 993 |
| a126 | m032-2004 | m | Nest2 | adult | 2004 | 1 | m | 4,633 | 979 |
| a127 | m007-2005 | m | FA5 | adult | 2005 | 1 | m | 3,874 | 992 |
| a128 | m015-2014 | m | FA17 | NA | 2014 | 1 | m | 5,849 | 985 |
| a129 | m016-2014 | m | FA17 | NA | 2014 | 1 | m | 4,158 | 990 |
| a13 | m021-2014 | m | FA17 | NA | 2014 | 1 | m | 3,504 | 988 |
| a130 | m008-2005 | m | FA5 | adult | 2005 | 1 | m | 3,846 | 993 |
| a131 | m009-2005 | m | FA6 | adult | 2005 | 1 | m | 5,906 | 993 |
| a132 | m018-2014 | m | FA17 | NA | 2014 | 1 | m | 4,435 | 991 |
| a133 | m033-2004 | m | Nest5 | adult | 2004 | 1 | m | 5,763 | 981 |
| a135 | m006-2018 | m | FA18 | pupa | 2018 | 1 | m | 5,023 | 989 |
| a136 | m020-2014 | m | FA15 | NA | 2014 | 1 | m | 4,364 | 990 |
| a138 | m010-2005 | m | FA6 | adult | 2005 | 1 | m | 6,237 | 989 |
| a139 | m011-2005 | m | FA6 | adult | 2005 | 1 | m | 3,401 | 992 |
| a141 | m035-2004 | m | Nest5 | adult | 2004 | 1 | m | 4,303 | 988 |
| a142 | m012-2005 | m | FA6 | adult | 2005 | 1 | m | 5,557 | 992 |
| a145 | m023-2014 | m | FA17 | NA | 2014 | 1 | m | 5,176 | 982 |
| a147 | m013-2005 | m | FA6 | adult | 2005 | 1 | m | 3,122 | 991 |
| a148 | m009-2018 | m | FA39 | pupa | 2018 | 1 | m | 4,412 | 990 |
| a149 | m037-2004 | m | Nest3 | adult | 2004 | 1 | m | 5,029 | 989 |
| a15 | m016-2018 | m | FA15 | pupa | 2018 | 1 | m | 4,039 | 983 |
| a150 | m038-2004 | m | Nest5 | adult | 2004 | 1 | m | 5,575 | 984 |
| a151 | m039-2004 | m | Nest5 | adult | 2004 | 1 | m | 5,173 | 989 |
| a152 | m024-2014 | m | FA15 | NA | 2014 | 1 | m | 4,226 | 988 |
| a153 | m025-2014 | m | FA15 | NA | 2014 | 1 | m | 5,279 | 986 |
| a155 | m012-2018 | m | FA38 | pupa | 2018 | 1 | m | 5,879 | 983 |
| a157 | m014-2018 | m | FA39 | pupa | 2018 | 1 | m | 4,420 | 991 |
| a158 | m040-2004 | m | Nest4 | adult | 2004 | 1 | m | 5,243 | 980 |
| a159 | m015-2018 | m | FA38 | pupa | 2018 | 1 | m | 4,658 | 985 |
| a16 | m019-2018 | m | FA24 | adult | 2018 | 1 | m | 4,432 | 987 |
| a160 | m014-2005 | m | FA10 | adult | 2005 | 1 | m | 4,335 | 989 |
| a161 | m015-2005 | m | FA6 | adult | 2005 | 1 | m | 3,694 | 983 |
| a162 | m016-2005 | m | FA5 | adult | 2005 | 1 | m | 3,258 | 987 |
| a163 | m026-2014 | m | FA17 | NA | 2014 | 1 | m | 4,352 | 989 |
| a164 | m017-2018 | m | FA38 | pupa | 2018 | 1 | m | 5,244 | 987 |
| a165 | m041-2004 | m | Nest4 | adult | 2004 | 1 | m | 5,179 | 989 |
| a167 | m018-2018 | m | FA24 | adult | 2018 | 1 | m | 4,941 | 977 |
| a168 | m042-2004 | m | Nest2 | adult | 2004 | 1 | m | 4,533 | 992 |
| a169 | m043-2004 | m | Nest2 | adult | 2004 | 1 | m | 6,200 | 986 |
| a17 | m032-2014 | m | FA15 | NA | 2014 | 1 | m | 3,430 | 989 |
| a172 | m021-2018 | m | FA39 | pupa | 2018 | 1 | m | 6,113 | 987 |
| a173 | m017-2005 | m | FA6 | adult | 2005 | 1 | m | 3,578 | 992 |
| a175 | m022-2018 | m | FA39 | pupa | 2018 | 1 | m | 3,924 | 987 |
| a176 | m030-2014 | m | FA17 | NA | 2014 | 1 | m | 4,718 | 978 |
| a177 | m044-2004 | m | Nest4 | adult | 2004 | 1 | m | 4,800 | 970 |
| a179 | m031-2014 | m | FA15 | NA | 2014 | 1 | m | 4,753 | 986 |
| a18 | m048-2004 | m | Nest4 | adult | 2004 | 1 | m | 4,655 | 987 |
| a180 | m018-2005 | m | FA5 | adult | 2005 | 1 | m | 4,718 | 989 |
| a181 | m046-2004 | m | Nest3 | adult | 2004 | 1 | m | 4,756 | 985 |
| a182 | m033-2014 | m | FA17 | NA | 2014 | 1 | m | 3,480 | 987 |
| a184 | m034-2014 | m | FA17 | NA | 2014 | 1 | m | 4,698 | 934 |
| a186 | m019-2005 | m | FA5 | adult | 2005 | 1 | m | 3,849 | 989 |
| a187 | m020-2005 | m | FA5 | adult | 2005 | 1 | m | 4,547 | 987 |
| a188 | m024-2018 | m | FA24 | adult | 2018 | 1 | m | 5,282 | 987 |
| a189 | m021-2005 | m | FA6 | adult | 2005 | 1 | m | 5,571 | 991 |
| a19 | m035-2014 | m | FA17 | NA | 2014 | 1 | m | 3,451 | 964 |
| a190 | m049-2004 | m | Nest4 | adult | 2004 | 1 | m | 5,830 | 986 |
| a191 | m022-2005 | m | FA6 | adult | 2005 | 1 | m | 4,788 | 991 |
| a192 | m023-2005 | m | FA6 | adult | 2005 | 1 | m | 3,482 | 991 |
| a20 | m025-2018 | m | FA18 | pupa | 2018 | 1 | m | 4,647 | 993 |
| a21 | m051-2004 | m | Nest2 | adult | 2004 | 1 | m | 4,249 | 988 |
| a22 | m026-2018 | m | FA39 | pupa | 2018 | 1 | m | 4,250 | 987 |
| a24 | m024-2005 | m | FA5 | adult | 2005 | 1 | m | 3,907 | 991 |
| a25 | m036-2014 | m | FA17 | NA | 2014 | 1 | m | 4,532 | 988 |
| a28 | m028-2018 | m | FA24 | adult | 2018 | 1 | m | 3,531 | 977 |
| a29 | m025-2005 | m | FA10 | adult | 2005 | 1 | m | 3,776 | 989 |
| a3 | m059-2004 | m | Nest3 | adult | 2004 | 1 | m | 3,552 | 990 |
| a30 | m029-2018 | m | FA38 | pupa | 2018 | 1 | m | 3,986 | 987 |
| a32 | m030-2018 | m | FA38 | pupa | 2018 | 1 | m | 4,800 | 986 |
| a33 | m037-2014 | m | FA15 | NA | 2014 | 1 | m | 3,729 | 988 |
| a35 | m026-2005 | m | FA10 | adult | 2005 | 1 | m | 4,088 | 985 |
| a36 | m027-2005 | m | FA6 | adult | 2005 | 1 | m | 3,466 | 991 |
| a37 | m057-2004 | m | Nest4 | adult | 2004 | 1 | m | 3,985 | 984 |
| a39 | m028-2005 | m | FA10 | adult | 2005 | 1 | m | 3,392 | 989 |
| a4 | m065-2004 | m | Nest5 | adult | 2004 | 1 | m | 6,171 | 985 |
| a40 | m060-2004 | m | Nest5 | adult | 2004 | 1 | m | 6,223 | 984 |
| a41 | m038-2014 | m | FA15 | NA | 2014 | 1 | m | 4,363 | 990 |
| a44 | m031-2018 | m | FA18 | pupa | 2018 | 1 | m | 4,393 | 991 |
| a45 | m063-2004 | m | Nest2 | adult | 2004 | 1 | m | 4,315 | 990 |
| a46 | m039-2014 | m | FA17 | NA | 2014 | 1 | m | 3,899 | 844 |
| a47 | m040-2014 | m | FA17 | NA | 2014 | 1 | m | 5,190 | 985 |
| a48 | m064-2004 | m | Nest3 | adult | 2004 | 1 | m | 3,866 | 986 |
| a49 | m029-2005 | m | FA6 | adult | 2005 | 1 | m | 4,328 | 988 |
| a5 | m032-2005 | m | FA5 | adult | 2005 | 1 | m | 4,010 | 990 |
| a51 | m030-2005 | m | FA10 | adult | 2005 | 1 | m | 3,701 | 988 |
| a52 | m066-2004 | m | Nest4 | adult | 2004 | 1 | m | 4,737 | 984 |
| a53 | m031-2005 | m | FA6 | adult | 2005 | 1 | m | 3,394 | 985 |
| a55 | m032-2018 | m | FA18 | pupa | 2018 | 1 | m | 4,929 | 985 |
| a56 | m033-2018 | m | FA38 | pupa | 2018 | 1 | m | 5,163 | 986 |
| a57 | m068-2004 | m | Nest2 | adult | 2004 | 1 | m | 5,023 | 988 |
| a58 | m034-2018 | m | FA15 | pupa | 2018 | 1 | m | 5,189 | 989 |
| a59 | m042-2014 | m | FA17 | NA | 2014 | 1 | m | 3,560 | 983 |
| a6 | m035-2005 | m | FA10 | adult | 2005 | 1 | m | 3,552 | 991 |
| a60 | m033-2005 | m | FA10 | adult | 2005 | 1 | m | 4,691 | 987 |
| a61 | m035-2018 | m | FA25 | pupa | 2018 | 1 | m | 4,296 | 988 |
| a62 | m069-2004 | m | Nest3 | adult | 2004 | 1 | m | 4,979 | 986 |
| a63 | m070-2004 | m | Nest2 | adult | 2004 | 1 | m | 3,974 | 989 |
| a64 | m043-2014 | m | FA17 | NA | 2014 | 1 | m | 4,614 | 901 |
| a66 | m036-2018 | m | FA24 | adult | 2018 | 1 | m | 4,670 | 980 |
| a67 | m045-2014 | m | FA15 | NA | 2014 | 1 | m | 3,438 | 991 |
| a68 | m034-2005 | m | FA6 | adult | 2005 | 1 | m | 4,119 | 989 |
| a69 | m046-2014 | m | FA15 | NA | 2014 | 1 | m | 4,003 | 990 |
| a70 | m047-2014 | m | FA17 | NA | 2014 | 1 | m | 3,940 | 987 |
| a71 | m037-2018 | m | FA24 | adult | 2018 | 1 | m | 3,958 | 979 |
| a72 | m071-2004 | m | Nest5 | adult | 2004 | 1 | m | 4,194 | 983 |
| a73 | m038-2018 | m | FA15 | pupa | 2018 | 1 | m | 4,292 | 989 |
| a74 | m072-2004 | m | Nest4 | adult | 2004 | 1 | m | 6,465 | 986 |
| a75 | m073-2004 | m | Nest2 | adult | 2004 | 1 | m | 5,091 | 990 |
| a76 | m036-2005 | m | FA5 | adult | 2005 | 1 | m | 5,296 | 987 |
| a81 | m039-2018 | m | FA25 | pupa | 2018 | 1 | m | 4,050 | 991 |
| a82 | m040-2018 | m | FA25 | pupa | 2018 | 1 | m | 4,672 | 989 |
| a83 | m076-2004 | m | Nest4 | adult | 2004 | 1 | m | 5,180 | 983 |
| a84 | m077-2004 | m | Nest4 | adult | 2004 | 1 | m | 4,734 | 986 |
| a85 | m078-2004 | m | Nest5 | adult | 2004 | 1 | m | 4,671 | 979 |
| a86 | m050-2014 | m | FA17 | NA | 2014 | 1 | m | 4,756 | 811 |
| a87 | m037-2005 | m | FA6 | adult | 2005 | 1 | m | 4,009 | 993 |
| a88 | m079-2004 | m | Nest4 | adult | 2004 | 1 | m | 4,838 | 988 |
| a89 | m080-2004 | m | Nest4 | adult | 2004 | 1 | m | 4,628 | 988 |
| a9 | m014-2011 | m | FA12 | NA | 2011 | 1 | m | 4,293 | 984 |
| a90 | m041-2018 | m | FA24 | adult | 2018 | 1 | m | 5,961 | 987 |
| a91 | m038-2005 | m | FA5 | adult | 2005 | 1 | m | 3,214 | 986 |
| a92 | m051-2014 | m | FA17 | NA | 2014 | 1 | m | 4,034 | 977 |
| a94 | m053-2014 | m | FA15 | NA | 2014 | 1 | m | 4,310 | 990 |
| a96 | m039-2005 | m | FA5 | adult | 2005 | 1 | m | 4,414 | 987 |
| a98 | m040-2005 | m | FA5 | adult | 2005 | 1 | m | 4,580 | 989 |
| a99 | m081-2004 | m | Nest2 | adult | 2004 | 1 | m | 5,703 | 990 |
| FA04_3m | m045-2018 | m | FA04 | adult | 2018 | 1 | m | 4,064 | 977 |
| FA07_1m | m051-2018 | m | FA07 | adult | 2018 | 1 | m | 4,009 | 984 |
| FA12_1m | m060-2018 | m | FA12 | adult | 2018 | 1 | m | 4,735 | 981 |
| FA12_2m | m061-2018 | m | FA12 | adult | 2018 | 1 | m | 4,316 | 973 |
| FA12_3m | m062-2018 | m | FA12 | adult | 2018 | 1 | m | 4,340 | 981 |
| FA12_4m | m063-2018 | m | FA12 | adult | 2018 | 1 | m | 5,307 | 979 |
| FA12_5m | m064-2018 | m | FA12 | adult | 2018 | 1 | m | 4,492 | 981 |
| FA12_6m | m065-2018 | m | FA12 | adult | 2018 | 1 | m | 5,372 | 984 |
| FA12_7m | m066-2018 | m | FA12 | adult | 2018 | 1 | m | 5,225 | 983 |
| FA15_1m | m069-2018 | m | FA15 | adult | 2018 | 1 | m | 5,328 | 985 |
| FA15_3m | m070-2018 | m | FA15 | adult | 2018 | 1 | m | 5,273 | 980 |
| FA16_1m | m073-2018 | m | FA16 | adult | 2018 | 1 | m | 4,262 | 985 |
| FA16_2m | m074-2018 | m | FA16 | adult | 2018 | 1 | m | 4,231 | 983 |
| FA16_3m | m075-2018 | m | FA16 | adult | 2018 | 1 | m | 4,535 | 984 |
| FAu14_1m | m079-2018 | m | FAu14 | adult | 2018 | 1 | m | 3,631 | 983 |
| s193 | m020-2021 | m | FA15 | adult_sexual | 2021 | 1 | m | 3,787 | 982 |
| s194 | m021-2021 | m | FA15 | adult_sexual | 2021 | 1 | m | 3,965 | 980 |
| s196 | m022-2020 | m | FA2014_2 | young_adult | 2020 | 1 | m | 4,156 | 984 |
| s197 | m023-2020 | m | FA39 | young_adult | 2020 | 1 | m | 4,601 | 982 |
| s198 | m024-2020 | m | FA2014_2 | young_adult | 2020 | 1 | m | 4,755 | 974 |
| s201 | m025-2020 | m | FA39 | young_adult | 2020 | 1 | m | 5,082 | 982 |
| s205 | m001-2011 | m | Katajikko | adult | 2011 | 1 | m | 3,587 | 949 |
| s209 | m027-2020 | m | FA2014_2 | young_adult | 2020 | 1 | m | 4,839 | 984 |
| s212 | m002-2011 | m | Katajikko | adult | 2011 | 1 | m | 3,907 | 937 |
| s213 | m028-2020 | m | FA2020 | young_adult | 2020 | 1 | m | 4,233 | 984 |
| s214 | m022-2021 | m | FA15 | adult_sexual | 2021 | 1 | m | 3,800 | 981 |
| s215 | m023-2021 | m | FA38 | adult_sexual | 2021 | 1 | m | 4,375 | 988 |
| s216 | m024-2021 | m | FA20 | adult_sexual | 2021 | 1 | m | 4,099 | 977 |
| s217 | m025-2021 | m | FAuus2014A2 | adult_sexual | 2021 | 1 | m | 2,773 | 994 |
| s222 | m026-2021 | m | FA38 | adult_sexual | 2021 | 1 | m | 3,943 | 982 |
| s223 | m029-2020 | m | FA39 | young_adult | 2020 | 1 | m | 4,332 | 986 |
| s224 | m030-2020 | m | FA2020 | young_adult | 2020 | 1 | m | 4,451 | 984 |
| s226 | m027-2021 | m | FA15 | adult_sexual | 2021 | 1 | m | 4,413 | 979 |
| s227 | m028-2021 | m | FA13 | adult_sexual | 2021 | 1 | m | 4,157 | 974 |
| s228 | m031-2020 | m | FA2014_2 | young_adult | 2020 | 1 | m | 4,210 | 979 |
| s232 | m029-2021 | m | FA20 | adult_sexual | 2021 | 1 | m | 3,443 | 986 |
| s233 | m030-2021 | m | FA15 | adult_sexual | 2021 | 1 | m | 4,303 | 986 |
| s234 | m031-2021 | m | FA15 | adult_sexual | 2021 | 1 | m | 4,237 | 980 |
| s235 | m003-2011 | m | Katajikko | adult | 2011 | 1 | m | 3,904 | 982 |
| s240 | m033-2021 | m | FA38 | adult_sexual | 2021 | 1 | m | 4,326 | 986 |
| s244 | m033-2020 | m | FA33 | young_adult | 2020 | 1 | m | 4,084 | 980 |
| s248 | m005-2011 | m | Katajikko | adult | 2011 | 1 | m | 3,221 | 986 |
| s249 | m034-2020 | m | FA2014_2 | young_adult | 2020 | 1 | m | 4,624 | 981 |
| s250 | m035-2020 | m | FA2020 | young_adult | 2020 | 1 | m | 4,261 | 981 |
| s251 | m036-2020 | m | FA39 | young_adult | 2020 | 1 | m | 4,770 | 981 |
| s252 | m035-2021 | m | FA38 | adult_sexual | 2021 | 1 | m | 3,077 | 972 |
| s253 | m036-2021 | m | FA38 | adult_sexual | 2021 | 1 | m | 3,783 | 983 |
| s255 | m037-2020 | m | FA2020 | young_adult | 2020 | 1 | m | 4,323 | 984 |
| s257 | m039-2020 | m | FA2020 | young_adult | 2020 | 1 | m | 4,630 | 983 |
| s259 | m040-2020 | m | FA33 | young_adult | 2020 | 1 | m | 4,108 | 984 |
| s261 | m006-2011 | m | Pohjrannw | NA | 2011 | 1 | m | 2,416 | 985 |
| s266 | m038-2021 | m | FA20 | adult_sexual | 2021 | 1 | m | 4,690 | 976 |
| s267 | m007-2011 | m | FA12 | adult | 2011 | 1 | m | 4,702 | 984 |
| s272 | m041-2020 | m | FA2020 | young_adult | 2020 | 1 | m | 5,002 | 984 |
| s273 | m040-2021 | m | FAuus2014A2 | adult_sexual | 2021 | 1 | m | 5,027 | 981 |
| s275 | m041-2021 | m | FA38 | adult_sexual | 2021 | 1 | m | 4,698 | 985 |
| s277 | m042-2021 | m | FAuus2014A2 | adult_sexual | 2021 | 1 | m | 3,591 | 952 |
| s278 | m043-2021 | m | FA38 | adult_sexual | 2021 | 1 | m | 3,883 | 946 |
| s279 | m044-2021 | m | FA38 | adult_sexual | 2021 | 1 | m | 4,186 | 980 |
| s281 | m042-2020 | m | FA33 | young_adult | 2020 | 1 | m | 4,669 | 981 |
| s284 | m008-2011 | m | FA12 | adult | 2011 | 1 | m | 3,636 | 983 |
| s285 | m043-2020 | m | FA2020 | young_adult | 2020 | 1 | m | 4,269 | 980 |
| s288 | m045-2021 | m | FA15 | adult_sexual | 2021 | 1 | m | 3,327 | 781 |
| s289 | m044-2020 | m | FA2014_2 | young_adult | 2020 | 1 | m | 4,804 | 979 |
| s291 | m013-2021 | m | FAuus2014A2 | adult_sexual | 2021 | 1 | m | 9,576 | 953 |
| s294 | m046-2021 | m | FA15 | adult_sexual | 2021 | 1 | m | 3,676 | 979 |
| s297 | m045-2020 | m | FA39 | young_adult | 2020 | 1 | m | 5,281 | 975 |
| s300 | m046-2020 | m | FA39 | young_adult | 2020 | 1 | m | 5,264 | 975 |
| s301 | m047-2021 | m | FAuus2014A2 | adult_sexual | 2021 | 1 | m | 4,774 | 988 |
| s302 | m047-2020 | m | FA39 | young_adult | 2020 | 1 | m | 3,786 | 613 |
| s304 | m048-2020 | m | FA2014_2 | young_adult | 2020 | 1 | m | 4,662 | 981 |
| s305 | m009-2011 | m | Katajikko | adult | 2011 | 1 | m | 3,847 | 985 |
| s307 | m049-2020 | m | FA2014_2 | young_adult | 2020 | 1 | m | 3,951 | 811 |
| s309 | m050-2020 | m | FA2020 | young_adult | 2020 | 1 | m | 4,414 | 983 |
| s312 | m051-2020 | m | FA39 | young_adult | 2020 | 1 | m | 4,562 | 977 |
| s315 | m010-2011 | m | Katajikko | adult | 2011 | 1 | m | 4,397 | 983 |
| s316 | m052-2020 | m | FA39 | young_adult | 2020 | 1 | m | 5,070 | 979 |
| s318 | m053-2020 | m | FA39 | young_adult | 2020 | 1 | m | 4,753 | 980 |
| s319 | m048-2021 | m | FAuus2014A2 | adult_sexual | 2021 | 1 | m | 4,582 | 977 |
| s321 | m049-2021 | m | FA20 | adult_sexual | 2021 | 1 | m | 3,970 | 974 |
| s322 | m050-2021 | m | FA38 | adult_sexual | 2021 | 1 | m | 4,059 | 981 |
| s325 | m051-2021 | m | FA20 | adult_sexual | 2021 | 1 | m | 4,234 | 985 |
| s327 | m052-2021 | m | FA15 | adult_sexual | 2021 | 1 | m | 4,247 | 981 |
| s330 | m053-2021 | m | FA20 | adult_sexual | 2021 | 1 | m | 4,717 | 987 |
| s335 | m054-2020 | m | FA39 | young_adult | 2020 | 1 | m | 4,640 | 973 |
| s337 | m055-2021 | m | FA38 | adult_sexual | 2021 | 1 | m | 4,317 | 985 |
| s339 | m056-2021 | m | FA38 | adult_sexual | 2021 | 1 | m | 4,142 | 987 |
| s341 | m055-2020 | m | FA2020 | young_adult | 2020 | 1 | m | 4,635 | 985 |
| s342 | m057-2021 | m | FA20 | adult_sexual | 2021 | 1 | m | 3,089 | 983 |
| s344 | m058-2021 | m | FA20 | adult_sexual | 2021 | 1 | m | 3,310 | 985 |
| s346 | m056-2020 | m | FA2014_2 | young_adult | 2020 | 1 | m | 4,489 | 982 |
| s347 | m057-2020 | m | FA2020 | young_adult | 2020 | 1 | m | 4,573 | 983 |
| s348 | m059-2021 | m | FAuus2014A2 | adult_sexual | 2021 | 1 | m | 2,970 | 953 |
| s349 | m058-2020 | m | FA2020 | young_adult | 2020 | 1 | m | 4,773 | 985 |
| s350 | m059-2020 | m | FA2014_2 | young_adult | 2020 | 1 | m | 4,891 | 982 |
| s351 | m060-2020 | m | FA2020 | young_adult | 2020 | 1 | m | 3,939 | 970 |
| s352 | m061-2020 | m | FA2014_2 | young_adult | 2020 | 1 | m | 4,445 | 978 |
| RN356 | FA33_09m | m | FA33 | adult | 2022 | 1 | m | 4,880 | 973 |
| RN359 | Bermuda21_10m | m | Bermuda21 | adult | 2022 | 1 | m | 4,597 | 975 |
| RN360 | FA33_04m | m | FA33 | adult | 2022 | 1 | m | 4,437 | 980 |
| RN362 | FA23_10m | m | FA23 | adult | 2022 | 1 | m | 4,618 | 976 |
| RN363 | FA12_05m | m | FA12 | adult | 2022 | 1 | m | 4,054 | 978 |
| RN364 | FA33_07m | m | FA33 | adult | 2022 | 1 | m | 3,340 | 981 |
| RN365 | FA23_04m | m | FA23 | adult | 2022 | 1 | m | 2,736 | 964 |
| RN367 | Bermuda21_01m | m | Bermuda21 | adult | 2022 | 1 | m | 2,897 | 974 |
| RN368 | FA12_04m | m | FA12 | adult | 2022 | 1 | m | 3,078 | 982 |
| RN369 | FA23_01m | m | FA23 | adult | 2022 | 1 | m | 2,799 | 973 |
| RN370 | FA12_03m | m | FA12 | adult | 2022 | 1 | m | 2,703 | 983 |
| RN372 | FA12_10m | m | FA12 | adult | 2022 | 1 | m | 2,434 | 980 |
| RN375 | FA23_03m | m | FA23 | adult | 2022 | 1 | m | 3,803 | 970 |
| RN376 | FA12_01m | m | FA12 | adult | 2022 | 1 | m | 3,797 | 981 |
| RN378 | FA33_03m | m | FA33 | adult | 2022 | 1 | m | 2,383 | 982 |
| RN379 | Bermuda21_06m | m | Bermuda21 | adult | 2022 | 1 | m | 5,227 | 966 |
| RN381 | Bermuda21_04m | m | Bermuda21 | adult | 2022 | 1 | m | 4,373 | 975 |
| RN382 | FA33_01m | m | FA33 | adult | 2022 | 1 | m | 4,413 | 978 |
| RN384 | FA33_08m | m | FA33 | adult | 2022 | 1 | m | 4,524 | 976 |
| RN385 | Bermuda21_05m | m | Bermuda21 | adult | 2022 | 1 | m | 4,197 | 978 |
| RN390 | FA23_09m | m | FA23 | adult | 2022 | 1 | m | 4,040 | 976 |
| RN391 | FA12_08m | m | FA12 | adult | 2022 | 1 | m | 4,361 | 976 |
| RN393 | FA12_07m | m | FA12 | adult | 2022 | 1 | m | 3,978 | 980 |
| RN396 | Bermuda21_08m | m | Bermuda21 | adult | 2022 | 1 | m | 3,709 | 974 |
| RN397 | Bermuda21_09m | m | Bermuda21 | adult | 2022 | 1 | m | 3,412 | 979 |
| RN398 | FA12_02m | m | FA12 | adult | 2022 | 1 | m | 3,429 | 977 |
| RN399 | FA33_10m | m | FA33 | adult | 2022 | 1 | m | 3,751 | 977 |
| RN400 | FA23_08m | m | FA23 | adult | 2022 | 1 | m | 3,836 | 977 |
| RN401 | FA33_02m | m | FA33 | adult | 2022 | 1 | m | 3,455 | 978 |
| RN403 | FA23_06m | m | FA23 | adult | 2022 | 1 | m | 3,672 | 968 |
| RN404 | FA23_07m | m | FA23 | adult | 2022 | 1 | m | 2,455 | 966 |
| RN405 | FA12_06m | m | FA12 | adult | 2022 | 1 | m | 2,761 | 964 |
| RN406 | FA23_02m | m | FA23 | adult | 2022 | 1 | m | 2,263 | 967 |
| RN407 | FA12_09m | m | FA12 | adult | 2022 | 1 | m | 2,729 | 979 |
| RN408 | FA23_05m | m | FA23 | adult | 2022 | 1 | m | 2,716 | 971 |
| RN409 | FA33_05m | m | FA33 | adult | 2022 | 1 | m | 2,478 | 981 |
| RN411 | Bermuda21_07m | m | Bermuda21 | adult | 2022 | 1 | m | 3,990 | 972 |
| RN412 | FA33_06m | m | FA33 | adult | 2022 | 1 | m | 3,472 | 976 |
| RN413 | Bermuda21_02m | m | Bermuda21 | adult | 2022 | 1 | m | 3,363 | 975 |
| RN414 | Bermuda21_03m | m | Bermuda21 | adult | 2022 | 1 | m | 2,896 | 974 |

**Table S5**. BDMI count and coverage data for the X(2) analysis.

| **threshold** | **total_dmis** | **intra_pairs** | **intra_perc** | **inter_pairs** | **inter_perc** | **total_bp** | **total_frac_genome** | **mean_region_size** | **min_region_size** | **max_region_size** |
| --- | --- | --- | --- | --- | --- | --- | --- | --- | --- | --- |
| -0.06 | 87 | 3 | 0.034 | 84 | 0.966 | 4250006 | 0.02 | 77972 | 3722 | 418576 |
| -0.0575 | 118 | 3 | 0.025 | 115 | 0.975 | 4974634 | 0.023 | 66804 | 3722 | 782744 |
| -0.055 | 122 | 2 | 0.016 | 120 | 0.984 | 5046008 | 0.024 | 63783 | 3722 | 707440 |
| -0.0525 | 118 | 4 | 0.034 | 114 | 0.966 | 7989334 | 0.038 | 100861 | 4485 | 1520567 |
| -0.05 | 121 | 4 | 0.033 | 117 | 0.967 | 8190308 | 0.038 | 104576 | 3722 | 932927 |
| -0.0475 | 158 | 7 | 0.044 | 151 | 0.956 | 12905987 | 0.061 | 120513 | 3722 | 1669642 |
| -0.045 | 251 | 7 | 0.028 | 244 | 0.972 | 16504482 | 0.078 | 126685 | 3722 | 1103575 |
| -0.0425 | 344 | 9 | 0.026 | 335 | 0.974 | 21029743 | 0.099 | 126604 | 3722 | 1103575 |
| -0.04 | 491 | 34 | 0.069 | 457 | 0.931 | 27425109 | 0.129 | 125085 | 3722 | 1587079 |
| -0.0375 | 600 | 51 | 0.085 | 549 | 0.915 | 32469870 | 0.152 | 127435 | 3722 | 1523317 |
| -0.035 | 714 | 29 | 0.041 | 685 | 0.959 | 40139759 | 0.188 | 129158 | 3722 | 1523317 |
| -0.0325 | 896 | 28 | 0.031 | 868 | 0.969 | 54618153 | 0.256 | 127508 | 3722 | 1523317 |
| -0.03 | 1095 | 27 | 0.025 | 1068 | 0.975 | 58169634 | 0.273 | 124053 | 3722 | 1520567 |
| -0.0275 | 1424 | 50 | 0.035 | 1374 | 0.965 | 71572328 | 0.336 | 129890 | 3722 | 1523317 |
| -0.025 | 1755 | 84 | 0.048 | 1671 | 0.952 | 84622362 | 0.397 | 129186 | 3722 | 1523317 |
| -0.0225 | 2197 | 122 | 0.056 | 2075 | 0.944 | 96539767 | 0.453 | 129974 | 3722 | 1669642 |
| -0.02 | 2712 | 167 | 0.062 | 2545 | 0.938 | 113286288 | 0.532 | 130550 | 3124 | 1669642 |
| -0.0175 | 3432 | 172 | 0.05 | 3260 | 0.95 | 129062034 | 0.606 | 138471 | 3722 | 1669642 |
| -0.015 | 4453 | 195 | 0.044 | 4258 | 0.956 | 144158359 | 0.677 | 139470 | 3177 | 1669642 |
| -0.0125 | 5760 | 250 | 0.043 | 5510 | 0.957 | 162572516 | 0.763 | 143102 | 3124 | 1669642 |
| -0.01 | 7267 | 310 | 0.043 | 6957 | 0.957 | 174258822 | 0.818 | 146984 | 3124 | 1669642 |
| -0.0075 | 9265 | 361 | 0.039 | 8904 | 0.961 | 183906587 | 0.864 | 150587 | 3177 | 1669642 |
| -0.005 | 11486 | 483 | 0.042 | 11003 | 0.958 | 188494233 | 0.885 | 153650 | 3177 | 1669642 |

**Table S6.** chi squared results for number BDMIs per scaffold. *X*(2) is *X*(2) analysis threshold. Obs. and Exp. are observed and expected BDMIs. Adjusted p values are Bonferroni corrected for multiple testing.

| **chrom** | **X(2)** | **Obs. BDMI** | **Exp. BDMI** | **chi^2^** | **p (adj.)** |
| --- | --- | --- | --- | --- | --- |
| Scaffold03 | -0.06 | 88 | 146 | 14 | 4.83e-03 |
| Scaffold03 | -0.0575 | 103 | 165 | 14 | 5.02e-03 |
| Scaffold03 | -0.055 | 124 | 187 | 12 | 1.18e-02 |
| Scaffold03 | -0.0525 | 149 | 216 | 12 | 1.48e-02 |
| Scaffold03 | -0.05 | 169 | 248 | 15 | 3.57e-03 |
| Scaffold03 | -0.0475 | 206 | 284 | 12 | 1.35e-02 |
| Scaffold03 | -0.045 | 259 | 326 | 7 | 1.70e-01 |
| Scaffold03 | -0.0425 | 318 | 381 | 6 | 5.10e-01 |
| Scaffold03 | -0.04 | 383 | 445 | 5 | 9.11e-01 |
| Scaffold03 | -0.0375 | 465 | 522 | 3 | 1.00e+00 |
| Scaffold03 | -0.035 | 559 | 614 | 2 | 1.00e+00 |
| Scaffold03 | -0.0325 | 698 | 732 | 1 | 1.00e+00 |
| Scaffold03 | -0.03 | 830 | 885 | 2 | 1.00e+00 |
| Scaffold03 | -0.0275 | 972 | 1073 | 5 | 7.14e-01 |
| Scaffold03 | -0.025 | 1169 | 1310 | 8 | 1.28e-01 |
| Scaffold03 | -0.0225 | 1392 | 1604 | 15 | 2.87e-03 |
| Scaffold03 | -0.02 | 1670 | 1974 | 26 | 1.18e-05 |
| Scaffold03 | -0.0175 | 1995 | 2436 | 44 | 7.26e-10 |
| Scaffold03 | -0.015 | 2454 | 3050 | 66 | 1.49e-14 |
| Scaffold03 | -0.0125 | 3027 | 3894 | 111 | 1.53e-24 |
| Scaffold03 | -0.01 | 3746 | 5122 | 220 | 2.31e-48 |
| Scaffold03 | -0.0075 | 4840 | 6932 | 388 | 7.56e-85 |
| Scaffold03 | -0.005 | 6637 | 9766 | 633 | 2.65e-138 |
| Scaffold17 | -0.06 | 1413 | 264 | 788 | 4.36e-172 |
| Scaffold17 | -0.0575 | 1642 | 297 | 935 | 5.88e-204 |
| Scaffold17 | -0.055 | 1902 | 337 | 1097 | 3.43e-239 |
| Scaffold17 | -0.0525 | 2218 | 389 | 1288 | 1.03e-280 |
| Scaffold17 | -0.05 | 2535 | 446 | 1471 | 2.00e-320 |
| Scaffold17 | -0.0475 | 2871 | 512 | 1655 | 0.00e+00 |
| Scaffold17 | -0.045 | 3212 | 588 | 1824 | 0.00e+00 |
| Scaffold17 | -0.0425 | 3637 | 687 | 2028 | 0.00e+00 |
| Scaffold17 | -0.04 | 4102 | 802 | 2241 | 0.00e+00 |
| Scaffold17 | -0.0375 | 4594 | 941 | 2436 | 0.00e+00 |
| Scaffold17 | -0.035 | 5126 | 1106 | 2624 | 0.00e+00 |
| Scaffold17 | -0.0325 | 5712 | 1320 | 2780 | 0.00e+00 |
| Scaffold17 | -0.03 | 6519 | 1595 | 3035 | 0.00e+00 |
| Scaffold17 | -0.0275 | 7403 | 1934 | 3262 | 0.00e+00 |
| Scaffold17 | -0.025 | 8473 | 2362 | 3520 | 0.00e+00 |
| Scaffold17 | -0.0225 | 9866 | 2891 | 3910 | 0.00e+00 |
| Scaffold17 | -0.02 | 11646 | 3558 | 4434 | 0.00e+00 |
| Scaffold17 | -0.0175 | 13790 | 4393 | 5035 | 0.00e+00 |
| Scaffold17 | -0.015 | 16529 | 5499 | 5771 | 0.00e+00 |
| Scaffold17 | -0.0125 | 20247 | 7021 | 6777 | 0.00e+00 |
| Scaffold17 | -0.01 | 25494 | 9234 | 8169 | 0.00e+00 |
| Scaffold17 | -0.0075 | 31628 | 12497 | 9080 | 0.00e+00 |
| Scaffold17 | -0.005 | 39133 | 17606 | 9190 | 0.00e+00 |

**Table S7.** Wilcoxon rank sum test results for distance to centromeres. P values are corrected for multiple testing (Bonferroni correction) within each analysis.

| **Analysis** | **Obs. median** | **Boot. median** | **W** | **p** | **p (adj.)** |
| --- | --- | --- | --- | --- | --- |
| gIMble | 2418184 | 42287 | 12383464 | 2.48e-12 | 2.48e-12 |
| X(2) -0.06 | 2809237 | 4220426 | 1104844 | 1.12e-07 | 2.57e-06 |
| X(2) -0.0575 | 2311584 | 4360588 | 942392 | 3.37e-09 | 7.75e-08 |
| X(2) -0.055 | 2316322 | 4192145.5 | 856228 | 7.58e-08 | 1.74e-06 |
| X(2) -0.0525 | 2878366 | 4459107.5 | 1556164 | 6.31e-08 | 1.45e-06 |
| X(2) -0.05 | 2609456 | 4617692.5 | 4104332 | 5.86e-15 | 1.35e-13 |
| X(2) -0.0475 | 2311584 | 4466590.5 | 9066680 | 2.59e-23 | 5.96e-22 |
| X(2) -0.045 | 1044832 | 4163179 | 17316882 | 2.10e-47 | 4.83e-46 |
| X(2) -0.0425 | 688747 | 4018955 | 35227564 | 7.02e-79 | 1.61e-77 |
| X(2) -0.04 | 793272 | 3918297.5 | 71252232 | 9.07e-88 | 2.09e-86 |
| X(2) -0.0375 | 1071584 | 4031945 | 88890126 | 1.69e-102 | 3.88e-101 |
| X(2) -0.035 | 1833192 | 4027233.5 | 152834358 | 5.46e-98 | 1.26e-96 |
| X(2) -0.0325 | 2294802 | 4215404 | 259332734 | 5.53e-98 | 1.27e-96 |
| X(2) -0.03 | 2621358 | 4154846.5 | 356180290 | 4.33e-92 | 9.97e-91 |
| X(2) -0.0275 | 2579866 | 4209004.5 | 652446318 | 1.07e-132 | 2.45e-131 |
| X(2) -0.025 | 2579866 | 4239258 | 1012827258 | 1.02e-165 | 2.35e-164 |
| X(2) -0.0225 | 2579866 | 4210440.5 | 1931505090 | 5.18e-212 | 1.19e-210 |
| X(2) -0.02 | 2345526 | 4241318.5 | 2914818130 | 2.04e-293 | 4.68e-292 |
| X(2) -0.0175 | 2379454 | 4249533 | 4674881678 | 0.00e+00 | 0.00e+00 |
| X(2) -0.015 | 2463600 | 4323157.5 | 7932668552 | 0.00e+00 | 0.00e+00 |
| X(2) -0.0125 | 2291478 | 4335050 | 1,3015E+10 | 0.00e+00 | 0.00e+00 |
| X(2) -0.01 | 2394696 | 4350845.5 | 2,2989E+10 | 0.00e+00 | 0.00e+00 |
| X(2) -0.0075 | 2290832 | 4400961 | 3,9541E+10 | 0.00e+00 | 0.00e+00 |
| X(2) -0.005 | 2294905 | 4409323 | 6,3385E+10 | 0.00e+00 | 0.00e+00 |

**Table S8.** Coverage and overlap between long-term gIMble barriers and candidate BDMIs.

| **x2_threshold** | **AD_total_cov** | **AD_gim_over** | **gim_total_cov** | **bp_dmi_only** | **bp_gim_only** | **perc_dmi_only** | **perc_gim_only** | **perc_both** |
| --- | --- | --- | --- | --- | --- | --- | --- | --- |
| -0.06 | 4250 | 535 | 17442 | 3715 | 16908 | 0.18 | 0.8 | 0.03 |
| -0.0575 | 4975 | 598 | 17442 | 4377 | 16845 | 0.2 | 0.77 | 0.03 |
| -0.055 | 5046 | 561 | 17442 | 4485 | 16881 | 0.2 | 0.77 | 0.03 |
| -0.0525 | 7989 | 671 | 17442 | 7318 | 16772 | 0.3 | 0.68 | 0.03 |
| -0.05 | 8190 | 755 | 17442 | 7436 | 16688 | 0.3 | 0.67 | 0.03 |
| -0.0475 | 12906 | 963 | 17442 | 11943 | 16479 | 0.41 | 0.56 | 0.03 |
| -0.045 | 16504 | 1240 | 17442 | 15265 | 16203 | 0.47 | 0.5 | 0.04 |
| -0.0425 | 21030 | 1678 | 17442 | 19352 | 15764 | 0.53 | 0.43 | 0.05 |
| -0.04 | 27425 | 1907 | 17442 | 25518 | 15535 | 0.59 | 0.36 | 0.04 |
| -0.0375 | 32470 | 2793 | 17442 | 29677 | 14649 | 0.63 | 0.31 | 0.06 |
| -0.035 | 40140 | 4073 | 17442 | 36067 | 13369 | 0.67 | 0.25 | 0.08 |
| -0.0325 | 54618 | 4952 | 17442 | 49666 | 12491 | 0.74 | 0.19 | 0.07 |
| -0.03 | 58170 | 5467 | 17442 | 52702 | 11975 | 0.75 | 0.17 | 0.08 |
| -0.0275 | 71572 | 7016 | 17442 | 64556 | 10426 | 0.79 | 0.13 | 0.09 |
| -0.025 | 84622 | 7708 | 17442 | 76914 | 9735 | 0.82 | 0.1 | 0.08 |
| -0.0225 | 96540 | 8757 | 17442 | 87783 | 8686 | 0.83 | 0.08 | 0.08 |
| -0.02 | 113286 | 10121 | 17442 | 103165 | 7321 | 0.86 | 0.06 | 0.08 |
| -0.0175 | 129062 | 11901 | 17442 | 117162 | 5542 | 0.87 | 0.04 | 0.09 |
| -0.015 | 144158 | 12792 | 17442 | 131366 | 4650 | 0.88 | 0.03 | 0.09 |
| -0.0125 | 162573 | 14441 | 17442 | 148132 | 3002 | 0.89 | 0.02 | 0.09 |
| -0.01 | 174259 | 15371 | 17442 | 158887 | 2071 | 0.9 | 0.01 | 0.09 |
| -0.0075 | 183907 | 15869 | 17442 | 168037 | 1573 | 0.91 | 0.01 | 0.09 |
| -0.005 | 188494 | 16235 | 17442 | 172260 | 1208 | 0.91 | 0.01 | 0.09 |

**Table S9**. List of 9 genes identified in regions with persistent BDMIs exhibiting no gene flow between *F. aquilonia* and *F. polyctena*.

| **Gene ID** | **Best BLAST hit** | **E-value** | **Identity (%)** | **Prediction** | **Gene description / function** |
| --- | --- | --- | --- | --- | --- |
| jg20385.t1 | [XM_029804083.1](https://www.ncbi.nlm.nih.gov/gene/?term=XM_029804083.1) | 8.17E-22 | 100.00 | *Formica exsecta* proteoglycan 4-like (LOC115233581), transcript variant X2 | *proteoglycan 4-like / DNA polymerase III subunit gamma/tau* |
| jg20385.t1 | [XM_029804081.1](https://www.ncbi.nlm.nih.gov/gene/?term=XM_029804081) | 8.17E-22 | 100.00 | *Formica exsecta* proteoglycan 4-like (LOC115233581), transcript variant X1 | *proteoglycan 4-like / DNA polymerase III subunit gamma/tau* |
| jg20386.t1 | [XM_029804080](https://www.ncbi.nlm.nih.gov/gene/?term=XM_029804080).1 | 0.00E+00 | 99.26 | *Formica exsecta* ras-related protein Rab-34 (LOC115233580) | *RAS oncogene family member RabX5 / enables GTP binding* Rab34 is required for the successive fusion of preciliary vesicles to generate ciliary vesicles and for the migration of the mother centriole from perinuclear region to plasma membrane. Silencing Rab34 by shRNA inhibits cell migration, invasion, and adhesion of breast cancer cells. |
| jg20387.t1 | [XM_029804088.1](https://www.ncbi.nlm.nih.gov/gene/?term=XM_029804088) | 5.82E-116 | 100.00 | *Formica exsecta* 60S ribosomal protein L23a (LOC115233585) | *ribosomal protein L23A / RNA binding, structure* The 60S ribosomal protein L23a (RPL23a) is a component of the large ribosomal subunit, which is responsible for protein synthesis in cells. It's involved in many cellular processes |
| jg20388.t1 | [XM_029804043.1](https://www.ncbi.nlm.nih.gov/gene/?term=XM_029804043) | 0 | 99.17 | *Formica exsecta* WASH complex subunit 4 (LOC115233553), transcript variant X3 | *trumpellin and WASH-interacting protein / Involved in endosomal transport/organization* |
| jg20388.t1 | [XM_029804042.1](https://www.ncbi.nlm.nih.gov/gene/?term=XM_029804042) | 0 | 99.17 | *Formica exsecta* WASH complex subunit 4 (LOC115233553), transcript variant X2 | *trumpellin and WASH-interacting protein / Involved in endosomal transport/organization* |
| jg20388.t1 | [XM_029804041.1](https://www.ncbi.nlm.nih.gov/gene/?term=XM_029804041) | 0 | 99.17 | *Formica exsecta* WASH complex subunit 4 (LOC115233553), transcript variant X1 | *strumpellin and WASH-interacting protein / Involved in endosomal transport/organization* |
| jg20389.t1 | [XM_029804044.1](https://www.ncbi.nlm.nih.gov/gene/?term=XM_029804044) | 1.34E-163 | 99.57 | *Formica exsecta* CUE domain-containing protein 1 (LOC115233554), transcript variant X1 | *CUE domain-containing protein 1 / enables ubiquitin binding* CUE domain-containing protein 1 (CUEDC1) is a protein that may be involved in breast cancer, lung cancer, and acute myeloid leukemia. |
| jg20390.t1 | [XM_050601544.1](https://www.ncbi.nlm.nih.gov/gene/?term=XM_050601544) | 0 | 97.95 | *Cataglyphis hispanica* ethanolaminephosphotransferase 1-like (LOC126854617) | *enables ethanolaminephosphotransferase 1-like / ethanolaminephosphotransferase activity* Ethanolaminephosphotransferase 1 (EPT1) is an enzyme that helps synthesize phospholipids and is important for neural development. |
| jg20391.t1 | [XM_029804033.1](https://www.ncbi.nlm.nih.gov/gene/?term=XM_029804033) | 1.94E-65 | 99.00 | *Formica exsecta* coiled-coil-helix-coiled-coil-helix domain-containing protein 7 (LOC115233546) | *coiled-coil-helix-coiled-coil-helix domain-containing protein 7* Coiled-coil-helix-coiled-coil-helix (CHCH) domain-containing protein 7 (CHCHD7) is primarily used for regulating mitochondrial function, specifically by maintaining copper homeostasis within the mitochondria; it plays a crucial role in the assembly and stability of the electron transport chain complexes, contributing to proper mitochondrial respiration |
| jg20392.t1 | [XM_029804032.1](https://www.ncbi.nlm.nih.gov/gene/?term=XM_029804032) | 5.49E-162 | 99.59 | *Formica exsecta* exosome complex component RRP40 (LOC115233545) | *exosome complex component Rrp40 / enables RNA binding* |
| jg20945.t1 | XM_029821704.1 | 2.21E-59 | 92.31 | *Formica exsecta* uncharacterized LOC115244228 (LOC115244228), transcript variant X1 | *Unknown* |
